# Selectivity and Potency of Microcystin Congeners against OATP1B1 and OATP1B3 Expressing Cancer Cells

**DOI:** 10.1371/journal.pone.0091476

**Published:** 2014-03-10

**Authors:** Timo H. J. Niedermeyer, Abigail Daily, Monika Swiatecka-Hagenbruch, Jeffrey A. Moscow

**Affiliations:** 1 Cyano Biotech GmbH, Berlin, Germany; 2 Interfaculty Institute for Microbiology and Infection Medicine, University of Tübingen, Tübingen, Germany; 3 Department of Pediatrics, University of Kentucky, Lexington, Kentucky, United States of America; University of Navarra School of Medicine and Center for Applied Medical Research (CIMA), Spain

## Abstract

Microcystins are potent phosphatase inhibitors and cellular toxins. They require active transport by OATP1B1 and OATP1B3 transporters for uptake into human cells, and the high expression of these transporters in the liver accounts for their selective hepatic toxicity. Several human tumors have been shown to have high levels of expression of OATP1B3 but not OATP1B1, the main transporter in liver cells. We hypothesized that microcystin variants could be isolated that are transported preferentially by OATP1B3 relative to OATP1B1 to advance as anticancer agents with clinically tolerable hepatic toxicity. Microcystin variants have been isolated and tested for cytotoxicity in cancer cells stably transfected with OATP1B1 and OATP1B3 transporters. Microcystin variants with cytotoxic OATP1B1/OATP1B3 IC_50_ ratios that ranged between 0.2 and 32 were found, representing a 150-fold range in transporter selectivity. As microcystin structure has a significant impact on transporter selectivity, it is potentially possible to develop analogs with even more pronounced OATP1B3 selectivity and thus enable their development as anticancer drugs.

## Introduction

**Figure 1:**
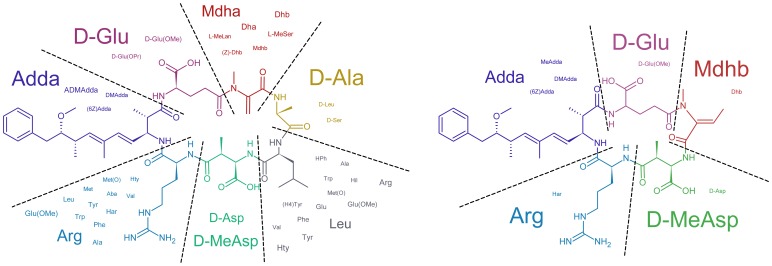
General structures of microcystins and nodularins. Prevalence of residues found within microcystins (left) and nodularins (right) is proportional to the font size of the respective residue. Data used to generate this figure is deposited at http://dx.doi.org/10.6084/m9.figshare.880756.

Microcystins (MCs) are cyclic heptapeptides produced by several cyanobacterial genera such as *Microcystis*, *Oscillatoria*, *Planktothrix*, *Nostoc*, and *Anabaena*. They can be considered to be among the best studied cyanobacterial secondary metabolites [1]–[4]. The common structural feature of microcystins is a polyketide synthase derived amino acid with the acronym “Adda”, (2S,3S,8S,9S)-3-amino-9-methoxy-2,6,8-trimethyl-10-phenyldeca-4,6-dienoic acid, which is also one of the two mandatory substructures for the potent protein serine/threonine phosphatase (PP) 1 and PP2A inhibition observed for the microcystins [5]–[9]. Not only is the biosynthesis of these compounds by polyketide synthases and non-ribosomal peptide synthetases remarkably well understood [10]–[12], but also more than 90 individual members of this chemically highly diverse compound family have been described in the scientific literature to date (Fig. 1) [13]–[56]. Especially prone to variation are the l-amino acids situated at positions 2 and 4 of the MC backbone. Related compounds are the nodularins, found in *Nodularia* sp., which also contain Adda but are cyclic pentapeptides instead of heptapeptides (Fig. 1) [57]–[60].

Being potent protein serine/threonine phosphatase inhibitors, microcystins and nodularins have a profound effect on cell signaling and cytoskeleton maintenance, leading to the death of affected cells [61], [62]. However, the relatively large and amphiphilic MCs are unable to cross cell membranes by passive diffusion. Instead, they rely on active uptake by cells. Three members of the organic anion transporting polypeptides (OATP) family are able to mediate this uptake of MCs, namely OATP1B1, 1B3 and 1A2 [63], [64]. OATP1B1 and OATP1B3 are the most efficient microcystin transporters, and as in healthy humans both transporters are exclusively found to be expressed in liver tissue [64], [65], microcystins and nodularins are known to cause extensive liver damage [66]–[68]. Thus microcystins became infamous as hepatotoxins causing harm to humans and cattle when these compounds accumulated in sources of drinking water during algal water bloom times [69], [70]. Inhibitors of these OATP transporters ameliorate the hepatotoxicity of microcystins and nodularins [68], [71], [72]. In contrast to OATP1B1, which is expressed in hepatocytes throughout the liver lobe, OATP1B3 localization is restricted around the central vein [64].

OATPs are currently in discussion as targets for cancer therapy [73]–[76]. Most interestingly, OATP1B3, but not OATP1B1, has been found to be functionally expressed in a number of cancer tissues, especially colon tumors, but also breast tumors, lung tumors, pancreatic and hepatocellular tumors [75], [77]–[79]. As differential toxicity of natural microcystin variants on cell lines expressing either OATP1B1 or OATP1B3 has been observed [78], [80], these findings raised the question whether microcystins might be suitable as leads for drug substances against these cancer types, and if there are microcystins among the more than 90 known variants that are selectively transported by OATP1B3 relative to OATP1B1. Selectivity that favors OATP1B3 over OATP1B1 should lead to a decreased hepatic clearance and increased uptake of MCs in OATP1B3-expressing tumors, creating a therapeutic window of the respective compound by decreasing the hepatic clearance rate and toxicity. MCs are interesting as novel lead structures because they have a mode of action not yet used but currently discussed for cancer therapy (phosphatase inhibition) [81]–[83], and in contrast to the majority of currently available anticancer drugs, they need active transport into cells and thus spare all tissues not expressing the mentioned OATPs.

We have isolated various microcystin congeners from cyanobacteria of the genera *Microcystis*, *Planktothrix*, and *Nodularia* to test the hypothesis whether different transporter selectivities might be attainable. As tools for selectivity testing, cervical cancer HeLa cells and colon cancer RKO cells stably transfected with expression vectors for OATP1B1 and 1B3 have been used. In the present manuscript, the results of the testing of isolated MCs on these cell lines are described. The determined IC_50_ values as well as the observed selectivity differences clearly show that small structural differences of the tested MCs indeed have a significant impact on transporter selectivity and cytotoxic potency.

## Results and Discussion

The amino acid (AA) compositions of the tested MCs are summarized in Table 1

**Table 1:** Structures of the tested MC congeners originating from ^a^ *Microcystis aeruginosa*, ^b^ *Planktothrix rubescens*, and ^c^ *Nodularia* sp.

	MC Congener	AA 1	AA 2	AA 3	AA 4	AA 5	AA 6	AA 7
**1** ^a^	MC-LR	Ala	Leu	MeAsp	Arg	Adda	Glu	Mdha
**2** ^a^	[d-Asp^3^]MC-LR	Ala	Leu	Asp	Arg	Adda	Glu	Mdha
**3** ^a^	[d-Asp^3^]MC-HilR	Ala	Hil	Asp	Arg	Adda	Glu	Mdha
**4** ^b^	[d-Asp^3^,(*E*)-Dhb^7^]MC-HilR	Ala	Hil	Asp	Arg	Adda	Glu	Dhb
**5** ^a^	MC-LY	Ala	Leu	MeAsp	Tyr	Adda	Glu	Mdha
**6** ^b^	[d-Asp^3^,(*E*)-Dhb^7^]MC-LY	Ala	Leu	Asp	Tyr	Adda	Glu	Dhb
**7** ^a^	LF	Ala	Leu	MeAsp	Phe	Adda	Glu	Mdha
**8** ^a^	LW	Ala	Leu	MeAsp	Trp	Adda	Glu	Mdha
**9** ^b^	[d-Asp^3^,(*E*)-Dhb^7^]-MC-LW	Ala	Leu	Asp	Trp	Adda	Glu	Dhb
**10** ^a^	RR	Ala	Arg	MeAsp	Arg	Adda	Glu	Mdha
**11** ^b^	[d-Asp^3^,(*E*)-Dhb^7^]MC-RR	Ala	Arg	Asp	Arg	Adda	Glu	Dhb
**12** ^a^	MC-RF	Ala	Arg	MeAsp	Phe	Adda	Glu	Mdha
**13** ^a^	YR	Ala	Tyr	MeAsp	Arg	Adda	Glu	Mdha
**14** ^a^	[d-Asp^3^]MC-YR	Ala	Tyr	Asp	Arg	Adda	Glu	Mdha
**15** ^b^	[d-Asp^3^,(*E*)-Dhb^7^]MC-YR	Ala	Tyr	Asp	Arg	Adda	Glu	Dhb
**16** ^a^	MC-RY	Ala	Arg	MeAsp	Tyr	Adda	Glu	Mdha
**17** ^a^	MC-YY	Ala	Tyr	MeAsp	Tyr	Adda	Glu	Mdha
**18** ^b^	[d-Asp^3^,(*E*)-Dhb^7^]MC-HtyY	Ala	Hty	Asp	Tyr	Adda	Glu	Dhb
**19** ^b^	[d-Asp^3^,(*E*)-Dhb^7^]MC-HtyHty	Ala	Hty	Asp	Hty	Adda	Glu	Dhb
**20** ^b^	[d-Asp^3^,(*E*)-Dhb^7^]MC-HtyW	Ala	Hty	Asp	Trp	Adda	Glu	Dhb
**21** ^a^	[d-Asp^3^,MSer^7^]MC-YHar	Ala	Tyr	Asp	Har	Adda	Glu	Mser
**22** ^a^	[d-Glu(OMe)^6^]MC-YR	Ala	Tyr	MeAsp	Arg	Adda	Glu(OMe)	Mdha
**23** ^c^	Nodularin			MeAsp	Arg	Adda	Glu	Mdhb

**Hil** homoisoleucine, **Hty** homotyrosine, **MeAsp** β-methylaspartic acid, **Har** homoarginine, **Mdha** *N*-methyl dehydroalanine, **Dhb** dehydrobutyric acid, **Mser** *N*-methyl serine, **Mdhb** *N*-methyl dehydrobutyric acid.

The structures of the microcystin congeners have been determined based on tandem HRMS [51], [52], [84], supported by ^1^H- and COSY-NMR spectroscopy. All MCs contain the characteristic Adda moiety at position 5 and d-Ala at position 1 of the molecule. Only one of the isolated congeners features an *O*-methylated d-iso-Glu instead of d-iso-Glu at position 6 (**22**). d-iso-β-MeAsp and d-iso-Asp in position 3 are almost equally distributed, as are Mdha and Dhb in position 7. The l amino acids in position 2 comprise Leu, Tyr, Arg, Hty, Hil (descending order of count), in position 4 Arg, Tyr, Trp, Phe, Hty, and Har are found. In addition to 22 MC variants, nodularin (**23**) as a cyclic Adda containing pentapeptide as well as okadaic acid (**24**) as a structurally not MC-related PP inhibitor have been tested.

The IC50 values of all isolated MCs against OATP1B1- and OATP1B3-expressing HeLa or RKO cell lines have been determined. The results are shown in Table 2.

**Table 2:** IC_50_ values of MC congeners (1-23) and okadaic acid (24) against stably transfected HeLa or RKO cell lines expressing either OATP1B1 or OATP1B3.

	IC50 (nM)						Ratio IC50 B1/B3	
	HeLa Control	HeLa B1	HeLa B3	RKO control	RKO B1	RKO B3	HeLa	RKO
1	>1000	1	5.1	>1000	8.2	26.9	0.2	0.3
2				>1000	7.3	31.3		0.2
3				>1000	57.05	142.4		0.4
4				993	56.9	3.8		15.0
5	>10	0.16	0.21				0.8	
6	>10	0.47	0.18				2.6	
7^a^	>1000	0.4	0.9				0.4	
8	>1000	0.22	0.16	>1000	0.4	0.29	1.4	1.4
9				>1000	11	7.7		1.4
10^a^	>10000	3800	580				6.6	
11				>100	>100	>100		Na
12				>1000	58.3	3.38		17.2
13^a^	>1000	90	45				2.0	
14				>1000	148.8	48		3.1
15				>1000	107.7	27.1		4.0
16	>1000	77	2.5				30.8	
17	259	1.5	0.8				1.9	
18	>10	0.515	0.078				6.6	
19	>10	0.39	0.18				2.2	
20				>100	4.9	2.57		1.9
21				>1000	625	385		1.6
22				>1000	595	227		2.6
23	>100	8.4	>100	>100	10	>100	< 0.1	< 0.1
24^a^	7.8	2.2	3				0.7	

a Transiently transfected cell lines; results were reported previously [78].

Interestingly, marked differences for both potency and selectivity of the individual MC congeners could be observed.

### Selectivity

While substitution of d-iso-β-MeAsp^3^ for d-iso-Asp^3^ (e.g. compounds **1**/**2** and **13**/**14**) seems not to have a significant influence on neither potency nor selectivity, the influence of the presence of either Mdha^7^ or (*E*)-Dhb^7^ (e.g. **3**/**4**, **5**/**6**, **8**/**9**, and **14**/**15**) is ambiguous: While in the case of **3**/**4** and **5**/**6** substitution of Mdha^7^ for (*E*)-Dhb^7^ lead to a profound increase in selectivity for OATP1B3 over OATP1B1, this effect was not observed for **8**/**9** and **14**/**15**. As the six least OATP1B3 selective compounds (**1**, **2**, **3**, **5**, **7**, **23**) all feature Mdha^7^ or Mdhb^7^ while (*E*)-Dhb^7^ is found in 3 of the 6 most OATP1B3 selective compounds (**4**, **10**, **12**, **15**, **16**, **18**), it is likely that (*E*)-Dhb^7^ has an influence in selectivity but is not the only structural feature conferring selectivity.

Indeed, especially the amino acid residues at positions 2 and 4 of the MC core structure seem to be important for both potency and selectivity. While 80% of the most OATP1B1 selective compounds feature Leu at position 2, this amino acid is completely absent in the most OATP1B3 selective compounds. However, Leu is also found in position 2 of several congeners that are weakly OATP1B3 selective (**6**, **8**, **9**), thus Leu alone does not make a compound OATP1B1 selective. Arg in position 2 seems to induce OATP1B3 selectivity, while Arg in position 4 does not have any influence on selectivity. This is obvious with compounds **13** and **16**, where exchange of the amino acids in positions 2 and 4 has a huge impact on selectivity. Arg in combination with the aromatic amino acids Phe, Tyr, and Hty are prominent monomers found in OATP1B3 selective compounds. Especially combinations of Arg^2^ and Phe^4^ (**12**) or Arg^2^ and Tyr^4^ (**16**) seem to confer OATP1B3 selectivity. But again, presence of Arg in position 2 seems not to be mandatory for OATP1B3 selectivity (**4**).

Interestingly, the pentapeptide Nodularin (**23**) is the most OATP1B1 selective among the Adda containing compounds examined.

### Potency

Potency of MC induced cell death in the test system used for this study has two facets. On one hand, toxicity of MC congeners depends on the extent of their PP inhibition [85]. On the other hand, potency is depending on transporting capacity of the respective OATP. These two effects have not been distinguished in the present study. While some previous studies suggest that *in vitro* and *in vivo* toxicity are related to enzyme inhibition rather than transport [78], [85], other reports show comparable PP inhibition of different microcystin congeners, implying that differences in transport contribute to *in vivo* toxicity [80], [86].

Not surprisingly, **22**, featuring *O*-methylated d-iso-Glu^6^, is one of the least potent compounds. Modifications of the free carboxylic acid of Glu are likely to lead to a loss of efficiency due to loss of interaction with a positively charged arginine in the active center of the protein phosphatases [7], and Glu(OMe) containing congeners have been found to inhibit PP1 to a much lesser extent than analogous Glu containing MCs [87] and have lower *in vivo* toxicity [26]. Interestingly, also **10**, **11** and **21** displayed weaker toxicity than most other congeners. While **10** inhibits PP2A about twenty times weaker compared with **1** [78], [80], [85], [86], its toxicity in the present assay is about 500 times lower. This indicates that in the case of **10** (and also its analog **11**), it is probable that toxicity is not only a matter of PP inhibition efficiency, but also of OATP transporting capability, as MC-RR is a dication under physiological conditions and thus less likely to be transported across cell membranes by anion transporters. In general, cytotoxicity in our assay does not correlate with protein phosphatase inhibition potency as described in the literature for compounds **1**, **2**, **6**, **7**, **8**, **9**, **10**, **13**, **18**, **19**, **20**, **23** [56], [78], [80], [85], [86]. Our data thus indicate that differing transport efficiencies have a higher impact on *in vivo* toxicity than differing protein phosphatase inhibition potency.

Interestingly, the most potent MCs, with IC_50_ values in the sub-nanomolar range (**5**, **6**, **7**, **8**, **17**, **18**, **19**), all feature combinations of either Leu^2^ and Tyr/Phe/Trp^4^ or Tyr/Hty^2^ and Tyr/Hty^4^. Arg is absent from the most potent congeners. The least potent congeners with IC_50_ values > 100 nM (**10**, **11**, **21**, **22**) all feature either Tyr^2^ and Arg/Har^4^ or Arg^2^ and Arg^4^. Presence of Arg in general lowers the potency, again supporting the hypothesis that the positively charged Arg residues hamper transport efficiency in addition to reducing PP2A inhibition, and thus lower *in vivo* toxicity.

## Summary and Conclusions

In our screening of 23 natural MC variants, we have found several MCs with an IC_50_ in the low nanomolar range and with transporter selectivity that favors OATP1B3 over OATP1B1 by a factor of up to thirty. While the presence of Arg in general lowers the potency of MC cytotoxicity, its presence in position 2 of the MC core structure also seems to be important for OATP1B3 selectivity, implying that this transporter might be more tolerant to the cationic nature of Arg under physiological conditions than OATP1B1. Furthermore, the presence of the slightly acidic aromatic amino acids Tyr or Hty in position 4 in addition to Arg in position 2 seems beneficial for OATP1B3 selectivity.

Further studies are needed to discriminate whether the observed differences in potency are due to differing PP inhibition or different transport capability of the OATP. However, earlier findings suggest that *in vivo* toxicity is mainly depending on OATP transport kinetics than on differences in PP inhibition [80], [86].

Although the deduced structure activity relationships are ambiguous in parts, the structural features observed for OATP1B3 selectivity can be condensed to the at present most likely selective general structure cyclo(-d-Ala^1^-l-Arg^2^-d-(Me)Asp^3^-l-aromatic amino acid^4^-Adda^5^-d-Glu^6^-(*E*)-Dhb^7^). This general structure can be used as a starting point for generating novel compounds e.g. by precursor feeding or biocombinatorial strategies.

A challenge of using MCs as drug leads still remains: Even with selective MC variants, liver toxicity still might be a significant challenge, making it necessary to generate MC variants that could be metabolically detoxified by healthy liver cells and/or efficiently effluxed into the bile, and thus take advantage of hepatic detoxification and clearance mechanisms that would not be found in tumors.

## Materials and Methods

### Cyanobacterial Material

Several *Microcystis aeruginosa* and *Planktothrix rubescens* strains as well as a *Nodularia* strain have been used to produce the studied microcystin congeners: **2** and **3**, *Microcystis aeruginosa* CBT 265; **4** and **15**, *Planktothrix rubescens* CBT 310; **5**, **16**, and **17**, *Microcystis aeruginosa* CBT 850, **6**, **9**, **18**, **19**, and **20**, *Planktothrix rubescens* CBT 862, **11**, *Planktothrix rubescens* CBT 329, **12**, *Microcystis aeruginosa* CBT 861, **14**, **21**, and **22**, *Microcystis aeruginosa* CBT 480, **23**, *Nodularia* sp. CBT 786. The strains were classified on the basis of PCR analysis and sequencing of various marker genes as well as their morphology, and have been deposited in the Cyano Biotech (CBT) culture collection under the accession numbers indicated above (Cyano Biotech, Berlin, Germany). The strains were cultivated in BG11 medium [88] at 20°C under continuous light (60-80 µmol m^-2^ s^-1^) in 20 L scale photobioreactors and harvested semi-continuously over a period of several weeks.

### Isolation of microcystins

Cyanobacteria strains were screened by HPLC-DAD/IT-TOF-MS (Kinetex C_18_, 2.6 µm, 100×3 mm column, phenomenex, Torrance, USA) using a linear scouting gradient of aqueous CH_3_CN (5 to 80% within 25 min at 0.6 ml/ min; 0.025% v/v TFA) to confirm MC presence by their characteristic UV spectrum as well as the characteristic tandem MS fragment ion at *m/z* 375 [52], [89]. Selected positive strains have been cultivated in BG11 medium [88] at 20°C under continuous light (60-80 µmol m^-2^ s^-1^) in 20 L scale photo bioreactors. After biomass harvest and freeze-drying, the biomasses were resuspended in 50% MeOH (v/v), treated with an ultrasonication rod (Bandelin, Berlin, Germany) and extracted on a shaker for 30 min. After centrifugation the biomasses were subsequently extracted using 80% MeOH (v/v). The 50% MeOH and 80% MeOH extracts of the respective biomasses were combined and dried *in vacuo*. The crude extracts were fractionated using water-methanol step gradients on C_18_ cartridges on a VersaFlash system (supelco, Bellefonte, USA), and the fractions containing MCs (monitored by HPLC) were dried *in vacuo*. After reconstitution, these fractions were subjected to semi-preparative HPLC. For a detailed description of the isolation procedures see File S1. Microcystins RR, LW, and LF and okadaic acid were obtained from Axxora, LLC (San Diego, CA), and microcystins LR and YR were obtained from Sigma-Aldrich (St. Louis, MO).

### Structure elucidation

The structures of the isolated MCs have been determined by high-resolution tandem mass spectrometry [51], [52], [84], and confirmed by one- and two-dimensional NMR spectroscopy. Samples for NMR spectroscopy were dissolved in 600 µL d_6_-DMSO. NMR spectra were recorded at 600 MHz (^1^H frequency) on a Bruker AV-III spectrometer using cryogenically cooled 5 mm TCI-triple resonance probes equipped with one-axis self-shielded gradients. DQF-COSY spectra [90] were recorded using 2048×512 complex data points using 8 scans. Spectra were referenced indirectly to tetramethylsilane via the residual signals of d_6_-DMSO (2.5 ppm for ^1^H). Tandem MS data have been acquired using an HPLC coupled to an IT-TOF mass spectrometer (Shimadzu Europe GmbH, Duisburg, Germany) with electrospray ionization in positive mode and were evaluated using the vendor’s software LCMSSolution version 3.60.361 with Formula Predictor version 1.13. For the calculation of sum formulae, the monoisotopic mass averaged from at least three scans has been used. The compounds were separated on a Kinetex C_18_ column (2.6 µm, 100×3 mm, phenomenex, Torrance, USA) using a gradient ranging from 5 to 80% CH_3_CN in water over 25 min (0.1% formic acid added as modifier). Precursor ions corresponding to [M+H]^+^ were isolated in the ion trap, fragmented by collision induced dissociation (CID) using argon as collision gas (collision energy set to 150%, collision gas to 100%, and q(Frequency) to 45.0 kHz), and separated in the TOF analyzer. MS/MS scans were averaged and converted to the mzXML format using the vendor’s software and evaluated using the software mMass (calculated fragment ions: M, a, b, c, and z; modifications: -H_2_O, -NH_3_, +CO, defined, combinations; matching with a tolerance of 0.01 Da) [91]. Annotated tandem HRMS and ^1^H-NMR spectra of all isolated compounds can be found in File S2.

### Cell lines and cytotoxicity assays

Primary hepatocytes are not suitable for studying microcystin toxicity and transport, as OATP transporters are downregulated within hours upon placing cells in culture [92]. Thus HeLa and RKO cell lines were obtained from ATCC and transfected with expression vectors for OATP1B1 and OATP1B3. Individual clones were isolated by a limiting dilution, and isolated clones showing high levels of expression by PCR were further characterized as previously described [72], [93]. Individual HeLa and RKO clones were selected for further study by demonstration of increased uptake [^3^H]-BQ123, a substrate for both transporters. Further, the OATP1B3 clones demonstrated increased uptake of [^3^H]-CCK-8, a specific substrate for OATP1B3 but not OATP1B1, while the OATP1B1 clones did not show uptake of [^3^H]-CCK-8. In addition, RKO and HeLa cells stably transfected with an empty expression vector were used as controls. For cytotoxicity assays, the cells were plated in triplicate in 96-well microtiter plates in a medium containing 5% fetal bovine serum at densities of 1000 cells/well. After 24 hours, medium containing MC structural variants was added to the cells. After another 3 days, cell survival was determined with a sulforhodamine-based assay as we have previously described [93]–[95]. As the dose response curves for the apoptosis causing microcystins are steep and go down to 0 within 72 hours, the sulforhodamine assay is indicative of cell death [72], [93]. The IC_50_ was calculated from the dose response curve as the concentration of drug that produced a 50% decrease in the mean absorbance compared to the untreated wells and reported as the average of at least three independent determinations performed in triplicate using Prism software.

## Supporting Information

File S1 Details on the Isolation of the Microcystin Congeners.
(PDF)

File S2 Annotated tandem HRMS and ^1^H-NMR spectra of all isolated compounds. Raw NMR and MS data of these compounds are available free of charge via the Internet at http://dx.doi.org/10.6084/m9.figshare.880755.
(PDF)
