# Supplementary material for: Selectivity and Potency of Microcystin Congeners against OATP1B1 and OATP1B3 Expressing Cancer Cells
Source: PLoS One. 2014 Mar 10;9(3):e91476. doi: 10.1371/journal.pone.0091476 (PMC3948918; doi:10.1371/journal.pone.0091476)
Supplement: File S1 — Details on the Isolation of the Microcystin Congeners. (PDF) [file pone.0091476.s001.pdf]

Supporting Information S1
Details on the Isolation of the Microcystin Congeners

Flash chromatography (FC) was done using a step gradient of 20%, 40%, 60%, 80%, and 100% MeOH in water (v/v). The fraction that has been used for subsequent isolation of the respective microcystin is indicated in the table below.

The following HPLC conditions were used to isolate the compounds that have not been obtained from commercial sources:

	FC	Column	flow rate [ml/min]	Gradient	t_r [min]
**2**	60%	Chromolith	5.0	1	12.2
**3**	60%	Chromolith	5.0	1	16.0
**4**	60%	Luna	4.5	2	35.5
**5**	60%	SymShield	2.5	3	58.8
**6**	60%	SymShield	2.5	4	39.9
**9**	60%	SymShield	2.5	4	46.1
**11**	60%	SymShield	2.5	5	33.8
**12**	60%	SymShield	2.5	6	57.4
**14**	60%	Luna	4.0	7	72.0
**15**	40%	Luna	4.5	2	38.6
**16**	60%	SymShield	2.5	3	53.0
**17**	60%	SymShield	2.5	3	58.2
**18**	60%	SymShield	2.5	4	39.3
**19**	60%	SymShield	2.5	4	38.5
**20**	60%	SymShield	2.5	4	44.6
**21**	60%	Luna	4.0	7	70.5
**22**	60%	Luna	4.0	7	86.5
**23**	60%	SymShield	2.5	8	28.3

Column	Column details
Chromolith	Chromolith SemiPrep RP-18e, 10×100 mm column (Merck, Darmstadt, Germany)
Luna	Luna C18(2), 5 µm, 10×250 mm column (phenomenex, Torrance, USA)
SymShield	SymmetryShield RP18, 5 µm, 10×250 mm column (Waters, Milford, USA)

Gradient	**Gradient details (**All mobile phases with 0.025% v/v TFA)
1	linear gradient of aqueous CH_3CN, starting with 45%, increasing to 60% in 22.5 min
2	linear gradient of aqueous MeOH, starting with 48%, increasing to 63% in 36 min
3	linear gradient of aqueous CH_3CN, starting with 29%, isocratic for 27.5 min, increasing to 40% in 7.5 min, isocratic at 40% for 7.5 min, increasing to 52.5% in 12.5 min and finally to 55% in 5 min
4	linear gradient of aqueous CH_3CN, starting with 20%, increasing to 32.5% in 20 min, stepping to 42.5% and increasing to 55% within 25 min
5	isocratic at 25% CH_3CN for 15 min, stepping to 28.5%, isocratic for 25 min
6	isocratic at 25% CH_3CN for 10 min, increasing to 30% in 34 min, increasing to 37.5% in 1.5 min, increasing to 47.5% in 11.5 min
7	linear gradient of aqueous CH_3CN, starting with 26%, increasing to 27.5% in 25 min, increasing to 47% in 25 min, increasing to 60% in 49 min
8	isocratic at 25% CH_3CN for 10 min, increasing to 50% in 30 min
