# Supplementary material for: Selectivity and Potency of Microcystin Congeners against OATP1B1 and OATP1B3 Expressing Cancer Cells
Source: PLoS One. 2014 Mar 10;9(3):e91476. doi: 10.1371/journal.pone.0091476 (PMC3948918; doi:10.1371/journal.pone.0091476)
Supplement: File S2 — Annotated tandem HRMS and 1H-NMR spectra of all isolated compounds. Raw NMR and MS data of these compounds are available free of charge via the Internet at http://dx.doi.org/10.6084/m9.figshare.880755. (PDF) [file pone.0091476.s002.pdf]

Supporting Information S2
Annotated tandem HRMS and ^{1}H-NMR spectra of all isolated compounds

Raw NMR and MS data of all compounds are available free of charge via the Internet at http://dx.doi.org/10.6084/m9.figshare.880755.

Analytical Data of the Microcystin Congeners ....

[D-Asp^3]MC-LR (2) 2
[D-Asp^3]MC-HilR (3) 5
[D-Asp^3,(*E*)-Dhb^7]MC-HilR (4) 8
MC-LY (5) 11
[D-Asp^3,(*E*)-Dhb^7]MC-LY (6) 14
[D-Asp^3,(*E*)-Dhb^7]-MC-LW (9) 16
[D-Asp^3,(*E*)-Dhb^7]MC-RR (11) 18
MC-RF (12) 21
[D-Asp^3]MC-YR (14) 24
[D-Asp^3,(*E*)-Dhb^7]MC-YR (15) 27
MC-RY (16) 30
MC-YY (17) 33
[D-Asp^3,(*E*)-Dhb^7]MC-HtyY (18) 36
[D-Asp^3,(*E*)-Dhb^7]MC-HtyHty (19) 39
[D-Asp^3,(*E*)-Dhb^7]MC-HtyW (20) 41
[D-Asp^3,MSer^7]MC-YHar (21) 44
[D-Glu(OMe)^6]MC-YR (22) 47
Nodularin (23) 50

[D-Asp[3]]MC-LR (2)

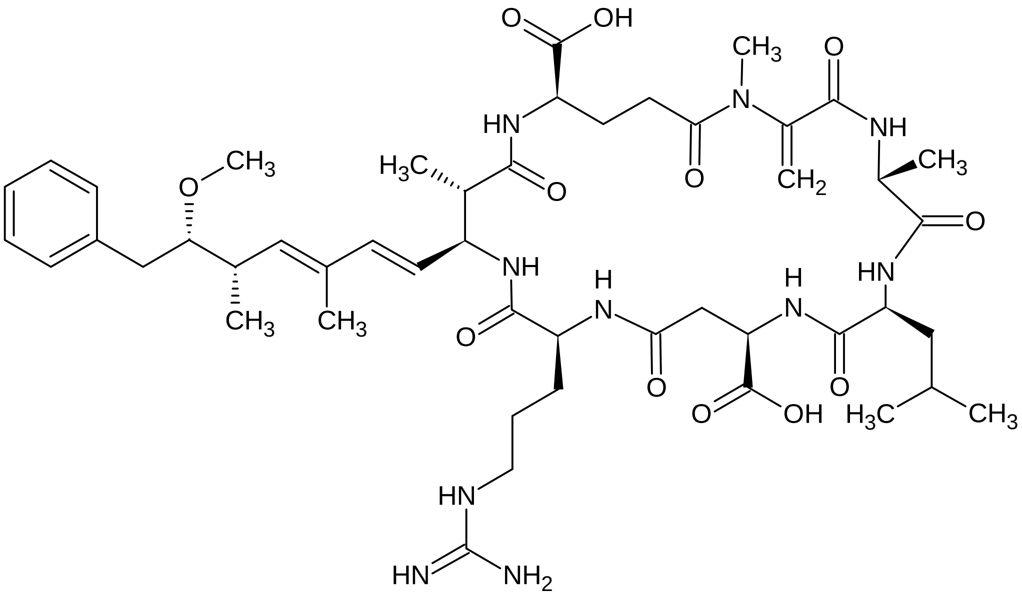

D-Ala | Leu | D-Asp | Arg | Adda | D-Glu | Mdha

Formula (M)	Ion	Meas. m/z	Pred. m/z	Diff (mDa)	Diff (ppm)	Iso Score
C48 H72 N10 O12	[M+H]+	981.5409	981.5404	0.5	0.51	99.51

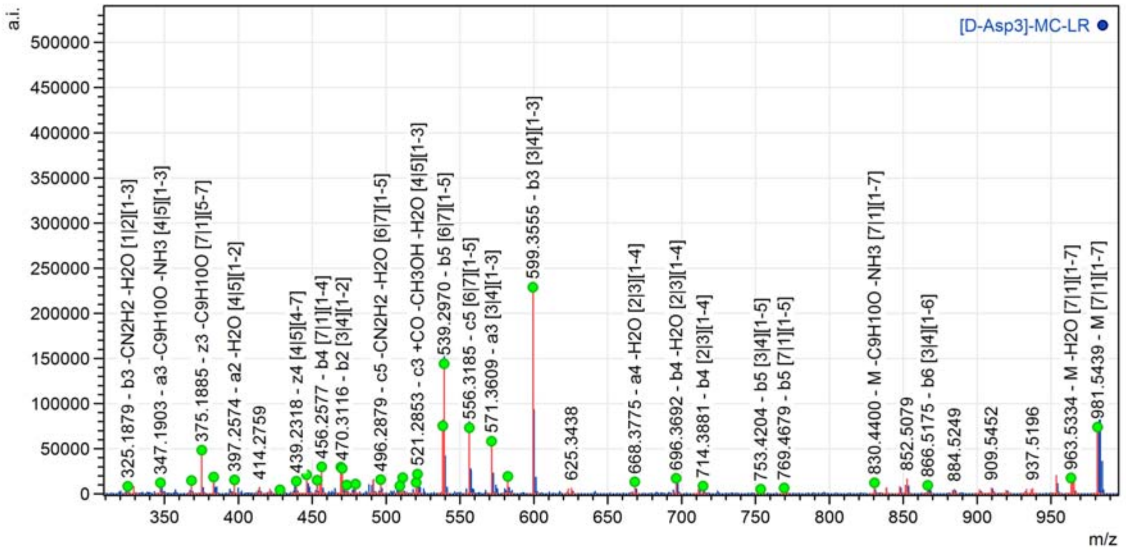

**Length**	**Mo. Mass**	**Av. Mass**	**Coverage**	**Matched Int.**
7 (Cyclic)	980.5331	981.1473	100.0 %	89 %

**Meas. m/z**	**Calc. m/z**	**δ (Da)**	**δ (ppm)**	**Rel. Int. (%)**	**Annotation**	**Formula**
325.1879	325.1870	0.0009	2.8	3.62	b3 -CN2H2 -H2O [1|2][1-3]	C15H24N4O4
347.1903	347.1965	-0.0062	-18.0	5.38	a3 -C9H10O -NH3 [4|5][1-3]	C19H26N2O4
368.1889	368.1928	-0.0040	-10.8	6.46	z3 [4|5][5-7]	C16H25N5O5
368.1889	368.1928	-0.0040	-10.8	6.46	b3 -NH3 [1|2][1-3]	C16H25N5O5
375.1885	375.1955	-0.0069	-18.5	21.06	z2 -CHN3H6 -NH3 [5|6][6-7]	C25H26O3
375.1885	375.1914	-0.0029	-7.8	21.06	z3 -C9H10O [7|1][5-7]	C20H26N2O5
375.1885	375.1914	-0.0029	-7.8	21.06	b3 -C9H10O -NH3 [4|5][1-3]	C20H26N2O5
375.1885	375.1914	-0.0029	-7.8	21.06	z3 -C9H10O -CH5N3 [5|6][5-7]	C20H26N2O5
383.1905	383.1925	-0.0020	-5.2	8.11	b4 [6|7][1-4]	C17H26N4O6
397.2574	397.2486	0.0088	22.2	6.75	a2 -H2O [4|5][1-2]	C24H32N2O3
428.2095	428.2180	-0.0085	-19.9	1.83	z4 -C9H10O -H2O [1|2][4-7]	C23H29N3O5
428.2095	428.2140	-0.0045	-10.5	1.83	c4 +CO [6|7][1-4]	C18H29N5O7
439.2318	439.2300	0.0019	4.3	6.03	z4 [4|5][4-7]	C19H30N6O6
439.2318	439.2300	0.0019	4.3	6.03	b4 -NH3 [7|1][1-4]	C19H30N6O6
446.2260	446.2286	-0.0025	-5.7	9.14	z4 -C9H10O [1|2][4-7]	C23H31N3O6
446.2260	446.2286	-0.0025	-5.7	9.14	b4 -C9H10O -NH3 [4|5][1-4]	C23H31N3O6
453.2806	453.2860	-0.0054	-12.0	6.42	b2 -NH3 [3|4][1-2]	C26H36N4O3
453.2806	453.2860	-0.0054	-12.0	6.42	z2 [5|6][6-7]	C26H36N4O3
456.2577	456.2565	0.0012	2.7	13.00	b4 [7|1][1-4]	C19H33N7O6
469.3261	469.3286	-0.0024	-5.2	12.87	c2 -H2O [3|4][1-2]	C26H40N6O2
470.3116	470.3126	-0.0010	-2.1	12.25	b2 [3|4][1-2]	C26H39N5O3
473.2171	473.2157	0.0015	3.1	4.09	b4 +CO -C9H11O -NH3 [4|5][1-4]	C24H30N3O7
479.2563	479.2613	-0.0050	-10.3	4.59	c5 -CH5N3 -H2O [6|7][1-5]	C22H34N6O6
479.2563	479.2613	-0.0050	-10.3	4.59	b3 +CO -C9H10O [2|3][1-3]	C22H34N6O6
479.2563	479.2613	-0.0050	-10.3	4.59	c3 +CO -C9H10O -NH3 [2|3][1-3]	C22H34N6O6
479.2563	479.2613	-0.0050	-10.3	4.59	b5 -CN2H2 -H2O [6|7][1-5]	C22H34N6O6
479.2563	479.2540	0.0023	4.7	4.59	a3 -CHN3H6 -NH3 [2|3][1-3]	C28H34N2O5
496.2879	496.2878	0.0001	0.2	6.82	c5 -CN2H2 -H2O [6|7][1-5]	C22H37N7O6
496.2879	496.2878	0.0001	0.2	6.82	c3 +CO -C9H10O [2|3][1-3]	C22H37N7O6
496.2879	496.2806	0.0073	14.7	6.82	a3 -CHN3H6 [2|3][1-3]	C28H37N3O5
509.2586	509.2646	-0.0060	-11.7	3.75	z3 -CN2H2 -NH3 [5|6][5-7]	C29H36N2O6
509.2586	509.2646	-0.0060	-11.7	3.75	b3 -CH5N3 -NH3 [2|3][1-3]	C29H36N2O6
509.2586	509.2646	-0.0060	-11.7	3.75	b3 -NH3 [4|5][1-3]	C29H36N2O6
509.2586	509.2646	-0.0060	-11.7	3.75	z3 [7|1][5-7]	C29H36N2O6
509.2586	509.2646	-0.0060	-11.7	3.75	z3 -CH5N3 [5|6][5-7]	C29H36N2O6
511.2990	511.2987	0.0003	0.7	7.74	a5 [6|7][1-5]	C22H38N8O6
520.3009	520.2990	0.0019	3.6	5.45	c5 -H2O -H2O [6|7][1-5]	C23H37N9O5
521.2853	521.2831	0.0022	4.3	9.45	b5 -H2O [6|7][1-5]	C23H36N8O6
521.2853	521.2758	0.0095	18.1	9.45	c3 +CO -CH3OH -H2O [4|5][1-3]	C29H36N4O5
538.3088	538.3096	-0.0008	-1.5	32.82	c5 -H2O [6|7][1-5]	C23H39N9O6
539.2970	539.2936	0.0034	6.3	62.85	b5 [6|7][1-5]	C23H38N8O7
556.3185	556.3202	-0.0017	-3.1	31.97	c5 [6|7][1-5]	C23H41N9O7
571.3609	571.3602	0.0006	1.1	25.40	a3 [3|4][1-3]	C30H46N6O5
582.3259	582.3286	-0.0027	-4.7	8.37	b3 -NH3 [3|4][1-3]	C31H43N5O6

582.3259	582.3286	-0.0027	-4.7	8.37	z3 [6|7][5-7]	C31H43N5O6
599.3555	599.3552	0.0003	0.5	100.00	b3 [3|4][1-3]	C31H46N6O6
668.3775	668.3766	0.0009	1.4	5.74	a4 -H2O [2|3][1-4]	C34H49N7O7
696.3692	696.3715	-0.0024	-3.4	7.46	b4 -H2O [2|3][1-4]	C35H49N7O8
714.3881	714.3821	0.0060	8.4	3.81	b4 [2|3][1-4]	C35H51N7O9
753.4204	753.4294	-0.0089	-11.9	2.29	b5 [3|4][1-5]	C38H56N8O8
769.4679	769.4607	0.0072	9.3	2.89	b5 [7|1][1-5]	C39H60N8O8
830.4400	830.4407	-0.0007	-0.8	5.25	M -C9H10O -NH3 [7|1][1-7]	C39H59N9O11
866.5175	866.5135	0.0041	4.7	4.10	b6 [3|4][1-6]	C44H67N9O9
963.5334	963.5298	0.0036	3.7	7.62	M -H2O [7|1][1-7]	C48H70N10O11
981.5439	981.5404	0.0035	3.5	32.15	M [7|1][1-7]	C48H72N10O12

NMR

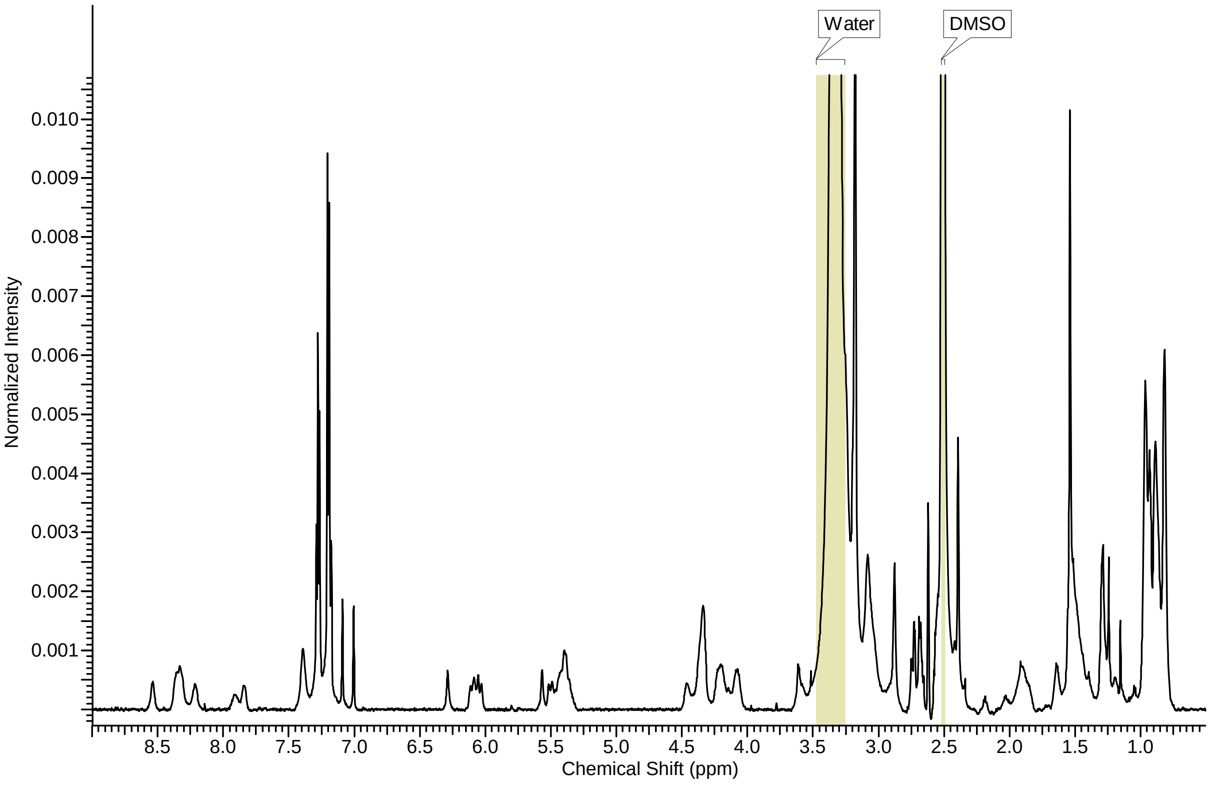

^{1}H-NMR spectrum agrees with the postulated structure.

[D-Asp^{3}]MC-HilR (3)

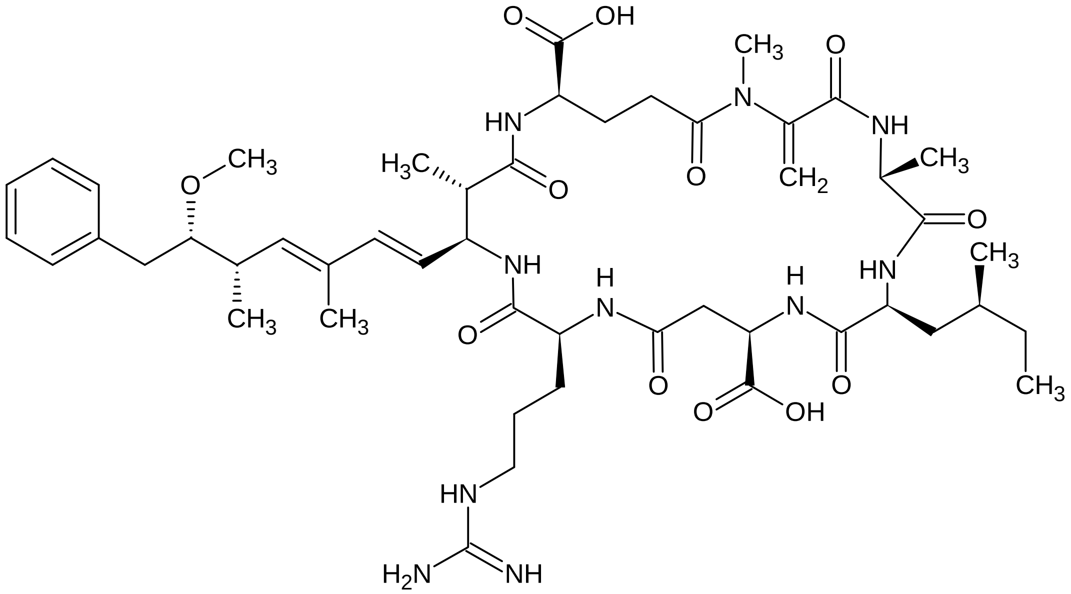

D-Ala | Hil | D-Asp | Arg | Adda | D-Glu | Mdha

Formula (M)	Ion	Meas. m/z	Pred. m/z	Diff (mDa)	Diff (ppm)	Iso Score
C49 H74 N10 O12	[M+H]+	995.5576	995.5560	1.6	1.61	90.24

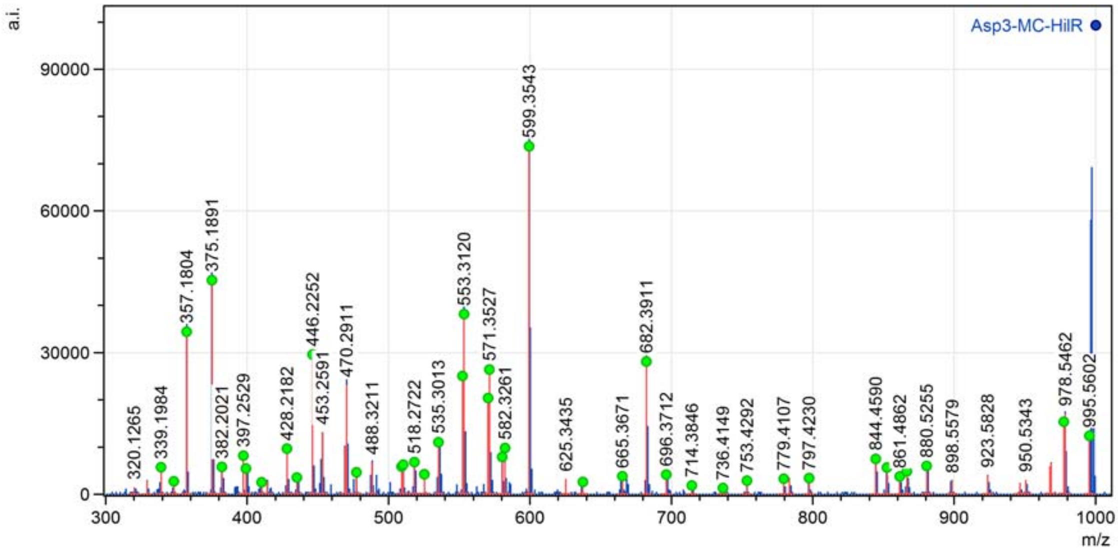

Length	Mo. Mass	Av. Mass	Coverage	Matched Int.
7 (Cyclic)	994.5488	995.1740	100.0 %	82.7 %

Meas. m/z	Calc. m/z	δ (Da)	δ (ppm)	Rel. Int. (%)	Annotation	Formula
339.1984	339.2027	-0.0043	-12.6	7.67	b3 -CN2H2 -H2O [1|2][1-3]	C16H26N4O4
348.1879	348.1918	-0.0039	-11.2	3.60	a4 -H2O -NH3 [5|6][1-4]	C18H25N3O4
357.1804	357.1809	-0.0004	-1.2	46.68	z3 -C9H10O -H2O [7|1][5-7]	C20H24N2O4
375.1891	375.1914	-0.0024	-6.3	61.48	z3 -C9H10O [7|1][5-7]	C20H26N2O5

382.2021	382.2085	-0.0064	-16.8	7.68	z3 [4|5][5-7]	C17H27N5O5
382.2021	382.2085	-0.0064	-16.8	7.68	b3 -NH3 [1|2][1-3]	C17H27N5O5
397.2529	397.2486	0.0043	10.8	10.91	a2 -H2O [4|5][1-2]	C24H32N2O3
399.2349	399.2350	-0.0001	-0.3	7.21	b3 [1|2][1-3]	C17H30N6O5
410.2330	410.2398	-0.0068	-16.5	3.24	c4 -H2O [5|6][1-4]	C19H31N5O5
410.2330	410.2398	-0.0068	-16.5	3.24	b4 -CN2H2 -H2O [7|1][1-4]	C19H31N5O5
428.2182	428.2180	0.0002	0.6	12.86	z4 -C9H10O -H2O [1|2][4-7]	C23H29N3O5
435.2423	435.2350	0.0073	16.7	4.59	z4 -H2O [4|5][4-7]	C20H30N6O5
435.2423	435.2350	0.0073	16.7	4.59	b4 -H2O -NH3 [7|1][1-4]	C20H30N6O5
446.2252	446.2286	-0.0034	-7.6	39.97	b4 -C9H10O -NH3 [4|5][1-4]	C23H31N3O6
446.2252	446.2286	-0.0034	-7.6	39.97	z4 -C9H10O [1|2][4-7]	C23H31N3O6
477.2363	477.2384	-0.0021	-4.3	5.99	b3 -CH3OH -NH3 [4|5][1-3]	C28H32N2O5
477.2363	477.2384	-0.0021	-4.3	5.99	z3 -CH3OH [7|1][5-7]	C28H32N2O5
477.2363	477.2344	0.0019	4.1	5.99	z5 -CH5N3 [4|5][3-7]	C23H32N4O7
477.2363	477.2344	0.0019	4.1	5.99	b5 -CH5N3 -NH3 [6|7][1-5]	C23H32N4O7
477.2363	477.2344	0.0019	4.1	5.99	z5 -CN2H2 -NH3 [4|5][3-7]	C23H32N4O7
509.2641	509.2646	-0.0006	-1.1	7.68	b3 -NH3 [4|5][1-3]	C29H36N2O6
509.2641	509.2646	-0.0006	-1.1	7.68	z3 [7|1][5-7]	C29H36N2O6
510.2981	510.2962	0.0018	3.6	8.24	a3 -CHN3H6 [3|4][1-3]	C29H39N3O5
510.2981	510.2922	0.0059	11.5	8.24	a4 -C9H10O -CN2H2 [2|3][1-4]	C24H39N5O7
518.2722	518.2762	-0.0040	-7.6	9.00	z3 -CH3OH -H2O [5|6][5-7]	C29H35N5O4
518.2722	518.2722	0.0001	0.1	9.00	z5 -H2O [4|5][3-7]	C24H35N7O6
518.2722	518.2722	0.0001	0.1	9.00	b5 -H2O -NH3 [6|7][1-5]	C24H35N7O6
525.3119	525.3184	-0.0065	-12.4	5.52	a3 -CH3OH [2|3][1-3]	C28H40N6O4
525.3119	525.3144	-0.0025	-4.7	5.52	a5 [6|7][1-5]	C23H40N8O6
525.3119	525.3071	0.0047	9.0	5.52	b3 -CN2H2 -H2O [2|3][1-3]	C29H40N4O5
525.3119	525.3071	0.0047	9.0	5.52	b3 -CH3OH -CN2H2 [3|4][1-3]	C29H40N4O5
525.3119	525.3071	0.0047	9.0	5.52	c3 -H2O [4|5][1-3]	C29H40N4O5
535.3013	535.3027	-0.0015	-2.7	14.77	b3 -CH3OH -H2O [2|3][1-3]	C29H38N6O4
535.3013	535.2987	0.0026	4.8	14.77	b5 -H2O [6|7][1-5]	C24H38N8O6
552.3241	552.3293	-0.0052	-9.4	33.88	c3 -CH3OH -H2O [2|3][1-3]	C29H41N7O4
552.3241	552.3253	-0.0012	-2.1	33.88	c5 -H2O [6|7][1-5]	C24H41N9O6
553.3120	553.3093	0.0027	4.9	51.64	b5 [6|7][1-5]	C24H40N8O7
570.3343	570.3398	-0.0055	-9.7	27.52	c3 -CH3OH [2|3][1-3]	C29H43N7O5
570.3343	570.3358	-0.0015	-2.6	27.52	c5 [6|7][1-5]	C24H43N9O7
571.3527	571.3602	-0.0075	-13.1	35.73	a3 [3|4][1-3]	C30H46N6O5
580.2999	580.3089	-0.0090	-15.6	10.50	b4 -C9H10O [2|3][1-4]	C26H41N7O8
580.2999	580.3017	-0.0018	-3.2	10.50	z4 [1|2][4-7]	C32H41N3O7
580.2999	580.3017	-0.0018	-3.2	10.50	b4 -NH3 [4|5][1-4]	C32H41N3O7
582.3261	582.3286	-0.0025	-4.3	13.09	b3 -NH3 [3|4][1-3]	C31H43N5O6
582.3261	582.3286	-0.0025	-4.3	13.09	z3 [6|7][5-7]	C31H43N5O6
599.3543	599.3552	-0.0009	-1.5	100.00	b3 [3|4][1-3]	C31H46N6O6
637.3342	637.3304	0.0038	6.0	3.39	a6 -NH3 [5|6][1-6]	C28H44N8O9
665.3671	665.3657	0.0014	2.1	5.06	b4 -NH3 [3|4][1-4]	C35H48N6O7
665.3671	665.3657	0.0014	2.1	5.06	z4 [7|1][4-7]	C35H48N6O7
682.3911	682.3923	-0.0012	-1.7	38.15	b4 [3|4][1-4]	C35H51N7O7
696.3712	696.3715	-0.0004	-0.5	5.60	b4 -H2O [2|3][1-4]	C35H49N7O8

714.3846	714.3821	0.0025	3.4	2.46	b4 [2|3][1-4]	C35H51N7O9
736.4149	736.4240	-0.0091	-12.3	1.70	b6 -C9H10O -CN2H2 [7|1][1-6]	C35H57N7O10
753.4292	753.4294	-0.0002	-0.3	3.90	b5 [3|4][1-5]	C38H56N8O8
779.4107	779.4087	0.0021	2.7	4.46	b5 -H2O [2|3][1-5]	C39H54N8O9
797.4230	797.4192	0.0038	4.8	4.56	b5 [2|3][1-5]	C39H56N8O10
844.4590	844.4563	0.0026	3.1	10.10	M -C9H10O -NH3 [7|1][1-7]	C40H61N9O11
852.5364	852.5342	0.0022	2.6	7.54	a6 [3|4][1-6]	C44H69N9O8
861.4862	861.4869	-0.0007	-0.8	4.97	a6 -H2O -NH3 [1|2][1-6]	C45H64N8O9
861.4862	861.4829	0.0033	3.8	4.97	M -C9H10O [7|1][1-7]	C40H64N10O11
866.5201	866.5135	0.0067	7.7	6.50	b6 [6|7][1-6]	C44H67N9O9
866.5201	866.5135	0.0067	7.7	6.50	a6 -H2O [7|1][1-6]	C44H67N9O9
880.5255	880.5291	-0.0036	-4.1	7.98	b6 [3|4][1-6]	C45H69N9O9
977.5485	977.5455	0.0030	3.0	20.64	M -H2O [7|1][1-7]	C49H72N10O11
995.5602	995.5560	0.0041	4.2	16.58	M [7|1][1-7]	C49H74N10O12

NMR

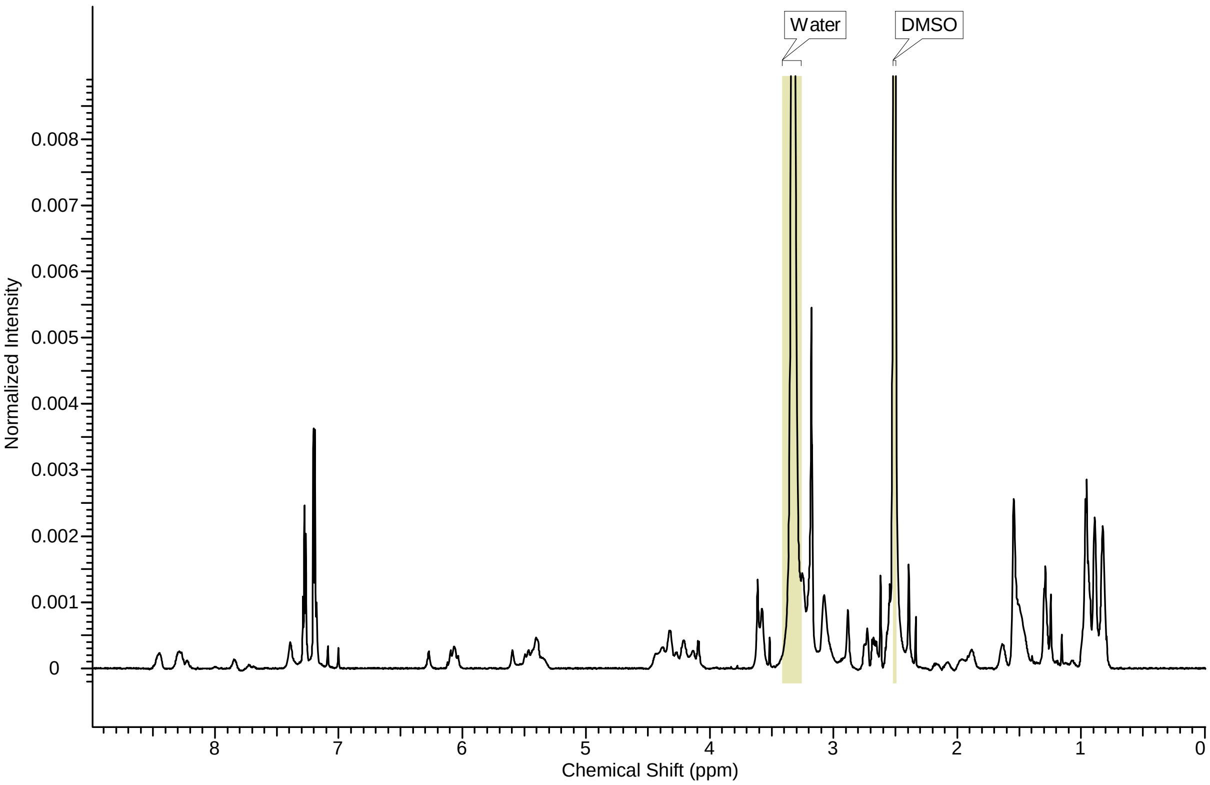

1H-NMR spectrum agrees with the postulated structure.

[D-Asp[3],(*E*)-Dhb[7]]MC-HilR (4)

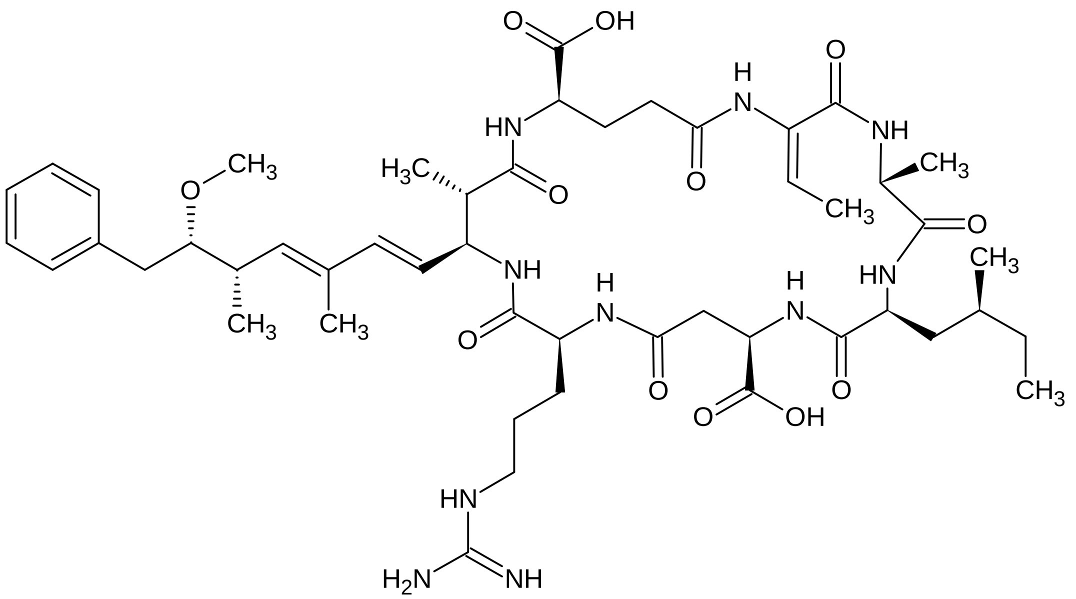

D-Ala | Hil | D-Asp | Arg | Adda | D-Glu | Dhb

Formula (M)	Ion	Meas. m/z	Pred. m/z	Diff (mDa)	Diff (ppm)	Iso Score
C49 H74 N10 O12	[M+H]+	995.5555	995.5560	-0.5	-0.50	86.4

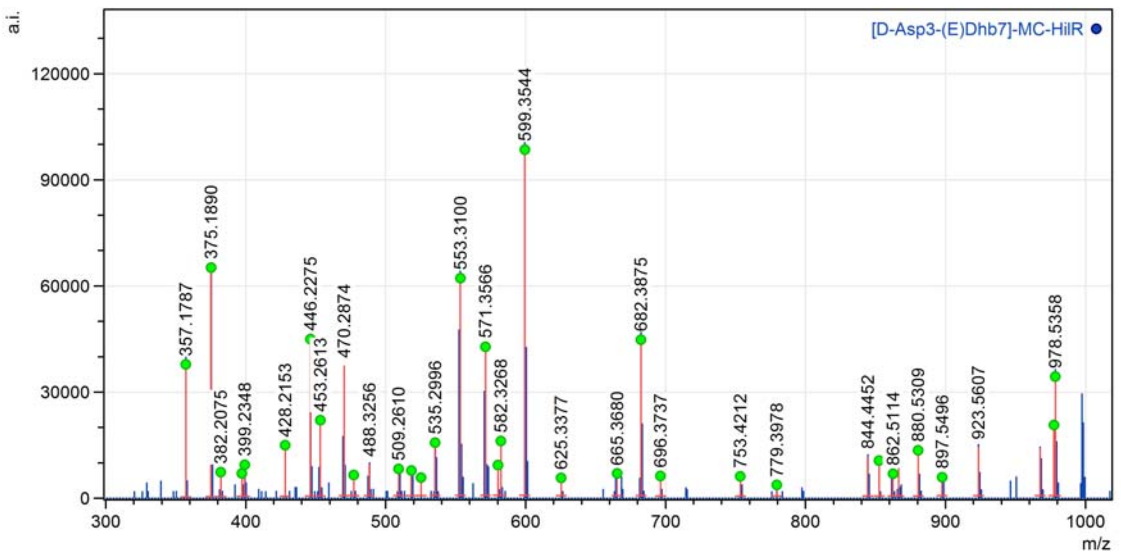

Length	Mo. Mass	Av. Mass	Coverage	Matched Int.
7 (Cyclic)	994.5488	995.1740	100.0 %	87.4 %

Meas. m/z	Calc. m/z	δ (Da)	δ (ppm)	Rel. Int. (%)	Annotation	Formula
357.1787	357.1809	-0.0022	-6.2	38.26	z3 -C9H10O -H2O [7|1][5-7]	C20H24N2O4
375.1890	375.1914	-0.0025	-6.7	66.27	z3 -C9H10O [7|1][5-7]	C20H26N2O5
382.2075	382.2085	-0.0010	-2.5	7.06	z3 [4|5][5-7]	C17H27N5O5
382.2075	382.2085	-0.0010	-2.5	7.06	b3 -NH3 [1|2][1-3]	C17H27N5O5

397.2566	397.2486	0.0081	20.3	6.70	a2 -H2O [4|5][1-2]	C24H32N2O3
399.2348	399.2350	-0.0003	-0.7	9.17	b3 [1|2][1-3]	C17H30N6O5
428.2153	428.2180	-0.0027	-6.4	14.71	z4 -C9H10O -H2O [1|2][4-7]	C23H29N3O5
446.2275	446.2286	-0.0011	-2.5	45.40	z4 -C9H10O [1|2][4-7]	C23H31N3O6
446.2275	446.2286	-0.0011	-2.5	45.40	b4 -C9H10O -NH3 [4|5][1-4]	C23H31N3O6
453.2613	453.2582	0.0031	6.9	21.93	c3 +CO -C9H11O -CN2H2 [2|3][1-3]	C21H34N5O6
477.2400	477.2384	0.0016	3.4	6.03	z3 -CH3OH [7|1][5-7]	C28H32N2O5
477.2400	477.2384	0.0016	3.4	6.03	z3 -CH3OH -CH5N3 [5|6][5-7]	C28H32N2O5
477.2400	477.2384	0.0016	3.4	6.03	b3 -CH3OH -NH3 [4|5][1-3]	C28H32N2O5
477.2400	477.2344	0.0057	11.8	6.03	z5 -CH5N3 [4|5][3-7]	C23H32N4O7
477.2400	477.2344	0.0057	11.8	6.03	b5 -CH5N3 -NH3 [6|7][1-5]	C23H32N4O7
509.2610	509.2646	-0.0036	-7.1	7.70	z3 -CH5N3 [5|6][5-7]	C29H36N2O6
509.2610	509.2646	-0.0036	-7.1	7.70	z3 [7|1][5-7]	C29H36N2O6
509.2610	509.2646	-0.0036	-7.1	7.70	b3 -NH3 [4|5][1-3]	C29H36N2O6
509.2610	509.2646	-0.0036	-7.1	7.70	b3 -CH5N3 -NH3 [2|3][1-3]	C29H36N2O6
518.2738	518.2762	-0.0023	-4.5	7.29	z3 -CH3OH -H2O [5|6][5-7]	C29H35N5O4
518.2738	518.2722	0.0017	3.3	7.29	z5 -H2O [4|5][3-7]	C24H35N7O6
518.2738	518.2722	0.0017	3.3	7.29	b5 -H2O -NH3 [6|7][1-5]	C24H35N7O6
525.3151	525.3184	-0.0033	-6.2	5.17	a3 -CH3OH [2|3][1-3]	C28H40N6O4
525.3151	525.3144	0.0007	1.4	5.17	a5 [6|7][1-5]	C23H40N8O6
535.2996	535.3027	-0.0032	-5.9	15.25	b3 -CH3OH -H2O [2|3][1-3]	C29H38N6O4
535.2996	535.2987	0.0008	1.6	15.25	b5 -H2O [6|7][1-5]	C24H38N8O6
553.3100	553.3133	-0.0033	-6.0	62.79	b3 -CH3OH [2|3][1-3]	C29H40N6O5
553.3100	553.3093	0.0007	1.3	62.79	b5 [6|7][1-5]	C24H40N8O7
571.3566	571.3602	-0.0037	-6.4	43.01	a3 [3|4][1-3]	C30H46N6O5
580.3033	580.3089	-0.0056	-9.7	8.75	b4 -C9H10O [2|3][1-4]	C26H41N7O8
580.3033	580.3017	0.0016	2.7	8.75	b4 -NH3 [4|5][1-4]	C32H41N3O7
580.3033	580.3017	0.0016	2.7	8.75	z4 [1|2][4-7]	C32H41N3O7
582.3268	582.3286	-0.0018	-3.1	15.70	b3 -NH3 [3|4][1-3]	C31H43N5O6
582.3268	582.3286	-0.0018	-3.1	15.70	z3 [6|7][5-7]	C31H43N5O6
599.3544	599.3552	-0.0007	-1.2	100.00	b3 [3|4][1-3]	C31H46N6O6
625.3377	625.3304	0.0073	11.6	5.11	c4 +CO -C9H10O [2|3][1-4]	C27H44N8O9
665.3680	665.3657	0.0023	3.5	6.45	z4 [7|1][4-7]	C35H48N6O7
665.3680	665.3657	0.0023	3.5	6.45	b4 -NH3 [3|4][1-4]	C35H48N6O7
682.3875	682.3923	-0.0048	-7.0	45.18	b4 [3|4][1-4]	C35H51N7O7
696.3737	696.3715	0.0022	3.2	5.76	b4 -H2O [2|3][1-4]	C35H49N7O8
753.4212	753.4294	-0.0082	-10.8	5.73	b5 [3|4][1-5]	C38H56N8O8
779.3978	779.3974	0.0004	0.5	3.20	a6 -CHN3H6 [2|3][1-6]	C40H54N6O10
852.5358	852.5342	0.0016	1.9	10.23	a6 [3|4][1-6]	C44H69N9O8
862.5114	862.5185	-0.0071	-8.3	6.40	b6 -H2O [3|4][1-6]	C45H67N9O8
880.5309	880.5291	0.0018	2.1	13.18	b6 [3|4][1-6]	C45H69N9O9
897.5496	897.5557	-0.0061	-6.8	5.39	c6 [3|4][1-6]	C45H72N10O9
977.5424	977.5455	-0.0031	-3.2	20.47	M -H2O [7|1][1-7]	C49H72N10O11
978.5358	978.5295	0.0063	6.4	34.48	M -NH3 [7|1][1-7]	C49H71N9O12

NMR

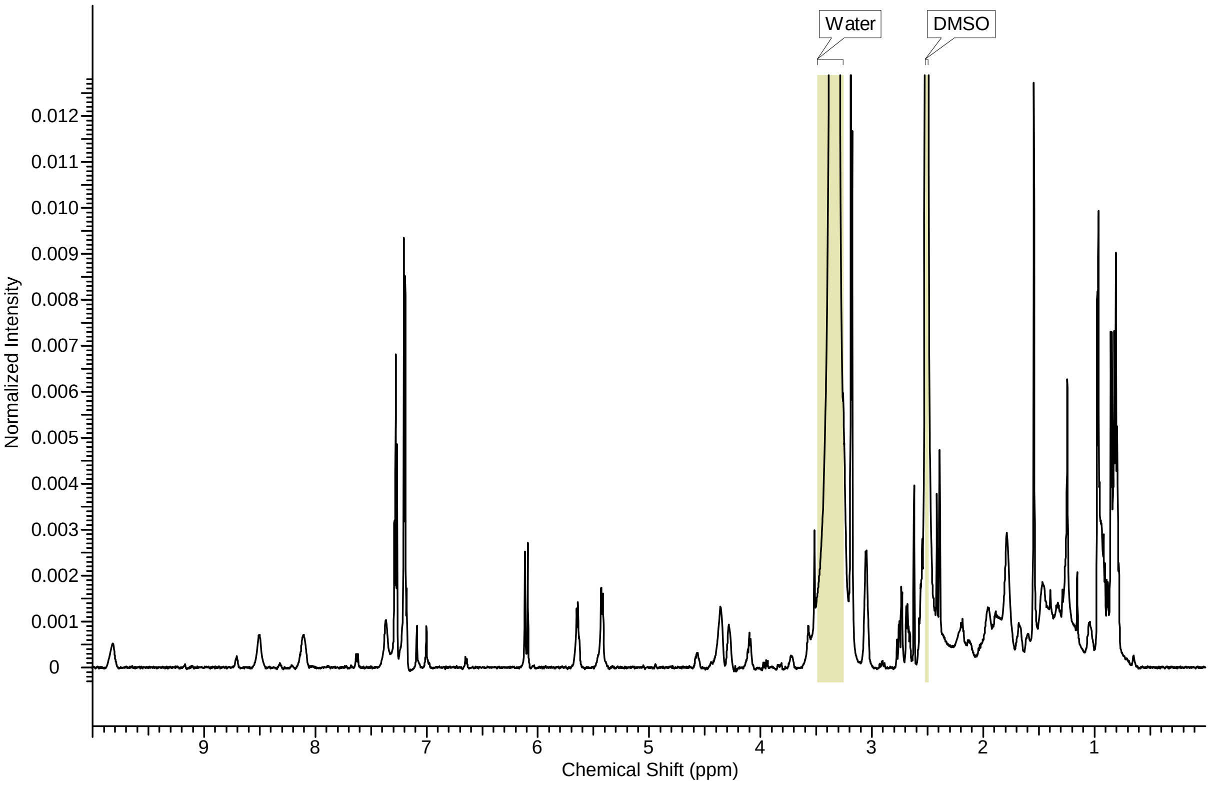

1H-NMR spectrum agrees with the postulated structure.

MC-LY (5)

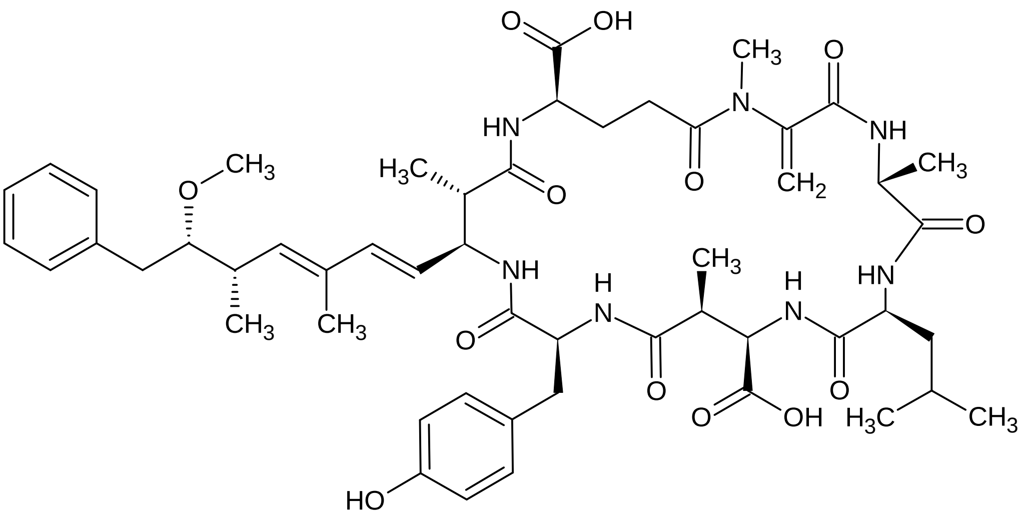

D-Ala | Leu | D-MeAsp | Tyr | Adda | D-Glu | Mdha

Formula (M)	Ion	Meas. m/z	Pred. m/z	Diff (mDa)	Diff (ppm)	Iso Score
C52 H71 N7 O13	[M+H]+	1002.5194	1002.5183	1.1	1.10	100.00

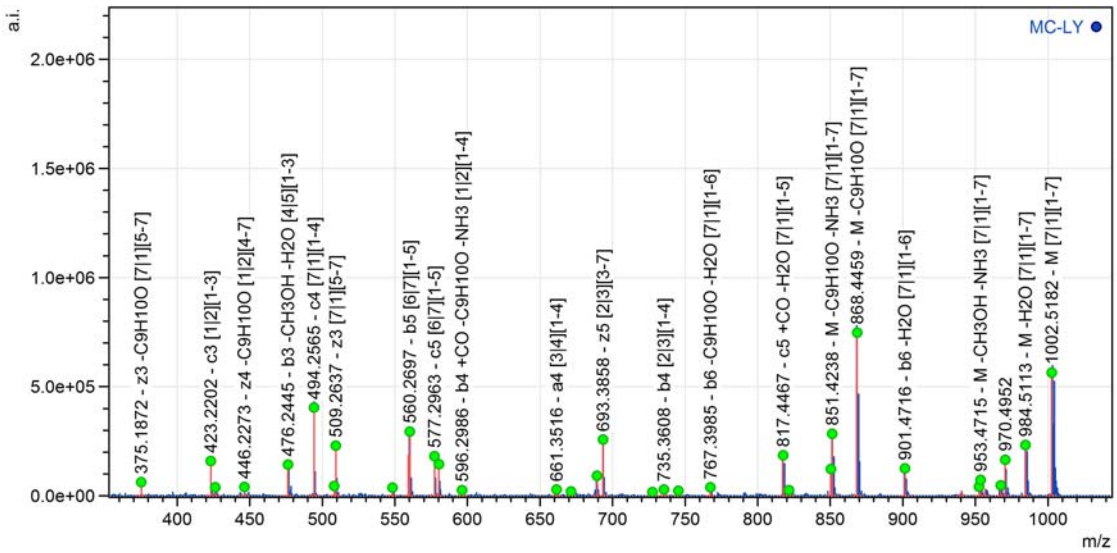

Length	Mo. Mass	Av. Mass	Coverage	Matched Int.
7 (Cyclic)	1001.5110	1002.1615	100.0 %	95.5 %

Meas. m/z	Calc. m/z	δ (Da)	δ (ppm)	Rel. Int. (%)	Annotation	Formula
375.1872	375.1914	-0.0043	-11.3	8.36	z3 -C9H10O [7|1][5-7]	C20H26N2O5
375.1872	375.1914	-0.0043	-11.3	8.36	b3 -C9H10O -NH3 [4|5][1-3]	C20H26N2O5
423.2202	423.2238	-0.0036	-8.4	21.30	c3 [1|2][1-3]	C20H30N4O6
426.2233	426.2275	-0.0042	-9.8	5.21	z2 [6|7][6-7]	C25H31NO5
426.2233	426.2275	-0.0042	-9.8	5.21	b2 -NH3 [4|5][1-2]	C25H31NO5
426.2233	426.2149	0.0084	19.7	5.21	a3 -C9H11O -NH3 [3|4][1-3]	C24H29N2O5
426.2233	426.2149	0.0084	19.7	5.21	a3 -C9H11O -NH3 [2|3][1-3]	C24H29N2O5

446.2273	446.2286	-0.0013	-2.9	5.45	b4 -C9H10O -NH3 [4|5][1-4]	C23H31N3O6
446.2273	446.2286	-0.0013	-2.9	5.45	z4 -C9H10O [1|2][4-7]	C23H31N3O6
476.2445	476.2544	-0.0099	-20.7	19.22	b3 -CH3OH -H2O [4|5][1-3]	C28H33N3O4
494.2565	494.2649	-0.0085	-17.1	54.10	b3 -CH3OH [4|5][1-3]	C28H35N3O5
494.2565	494.2609	-0.0044	-9.0	54.10	c4 [7|1][1-4]	C23H35N5O7
508.2389	508.2402	-0.0013	-2.5	5.99	b5 -H2O [5|6][1-5]	C23H33N5O8
509.2637	509.2646	-0.0009	-1.9	30.70	b3 -NH3 [4|5][1-3]	C29H36N2O6
509.2637	509.2646	-0.0009	-1.9	30.70	z3 [7|1][5-7]	C29H36N2O6
548.2722	548.2755	-0.0033	-6.1	5.01	z4 -CH3OH [1|2][4-7]	C31H37N3O6
548.2722	548.2755	-0.0033	-6.1	5.01	b4 -CH3OH -NH3 [4|5][1-4]	C31H37N3O6
560.2697	560.2715	-0.0018	-3.1	39.35	b5 [6|7][1-5]	C27H37N5O8
577.2963	577.2980	-0.0017	-3.0	24.24	c5 [6|7][1-5]	C27H40N6O8
580.2995	580.3017	-0.0022	-3.9	19.37	z4 [1|2][4-7]	C32H41N3O7
580.2995	580.3017	-0.0022	-3.9	19.37	b4 -NH3 [4|5][1-4]	C32H41N3O7
596.2986	596.2966	0.0019	3.2	3.43	b4 +CO -C9H10O -NH3 [1|2][1-4]	C32H41N3O8
661.3516	661.3596	-0.0080	-12.1	3.88	a4 [3|4][1-4]	C37H48N4O7
671.3059	671.3035	0.0024	3.6	2.60	b6 -H2O [5|6][1-6]	C32H42N6O10
689.3179	689.3141	0.0038	5.5	12.28	b6 [5|6][1-6]	C32H44N6O11
693.3858	693.3858	-0.0000	-0.0	34.39	b5 -NH3 [4|5][1-5]	C38H52N4O8
693.3858	693.3858	-0.0000	-0.0	34.39	z5 [2|3][3-7]	C38H52N4O8
727.3371	727.3338	0.0034	4.6	2.30	b4 +CO -H2O -H2O [2|3][1-4]	C40H46N4O9
735.3608	735.3600	0.0008	1.1	3.80	b4 [2|3][1-4]	C39H50N4O10
745.3436	745.3443	-0.0007	-0.9	2.98	b4 +CO -H2O [2|3][1-4]	C40H48N4O10
767.3985	767.3974	0.0011	1.5	5.14	b6 -C9H10O -H2O [7|1][1-6]	C39H54N6O10
767.3985	767.3974	0.0011	1.5	5.14	b6 +CO -C9H10O [6|7][1-6]	C39H54N6O10
767.3985	767.3974	0.0011	1.5	5.14	b6 +CO -C9H10O [3|4][1-6]	C39H54N6O10
817.4467	817.4495	-0.0027	-3.4	24.85	c5 +CO -H2O [7|1][1-5]	C44H60N6O9
821.4473	821.4444	0.0029	3.5	3.41	b6 -H2O [4|5][1-6]	C43H60N6O10
850.4381	850.4345	0.0035	4.2	16.25	M -C9H10O -H2O [7|1][1-7]	C43H59N7O11
851.4238	851.4185	0.0053	6.2	37.90	M -C9H10O -NH3 [7|1][1-7]	C43H58N6O12
868.4459	868.4451	0.0008	0.9	100.00	M -C9H10O [7|1][1-7]	C43H61N7O12
901.4716	901.4706	0.0010	1.1	16.62	b6 -H2O [7|1][1-6]	C48H64N6O11
901.4716	901.4706	0.0010	1.1	16.62	b6 +CO [3|4][1-6]	C48H64N6O11
901.4716	901.4706	0.0010	1.1	16.62	b6 +CO [6|7][1-6]	C48H64N6O11
952.4794	952.4815	-0.0021	-2.2	5.46	M -CH3OH -H2O [7|1][1-7]	C51H65N7O11
953.4715	953.4655	0.0060	6.3	9.60	M -CH3OH -NH3 [7|1][1-7]	C51H64N6O12
967.4872	967.4811	0.0061	6.3	6.23	M -H2O -NH3 [7|1][1-7]	C52H66N6O12
970.4952	970.4920	0.0031	3.2	22.01	M -CH3OH [7|1][1-7]	C51H67N7O12
984.5113	984.5077	0.0036	3.6	31.07	M -H2O [7|1][1-7]	C52H69N7O12
1002.5182	1002.5183	-0.0000	-0.0	75.33	M [7|1][1-7]	C52H71N7O13

NMR

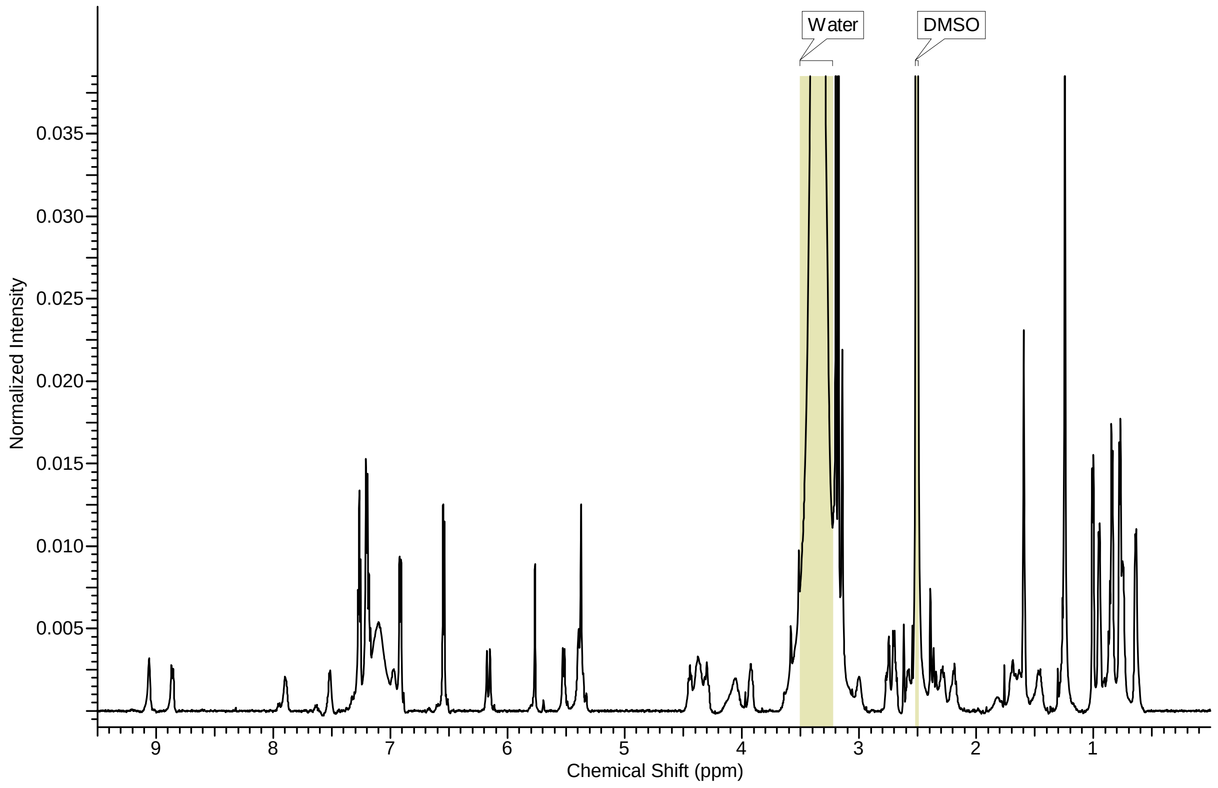

^{1}H-NMR spectrum agrees with the postulated structure.

[D-Asp^3,(*E*)-Dhb^7]MC-LY (6)

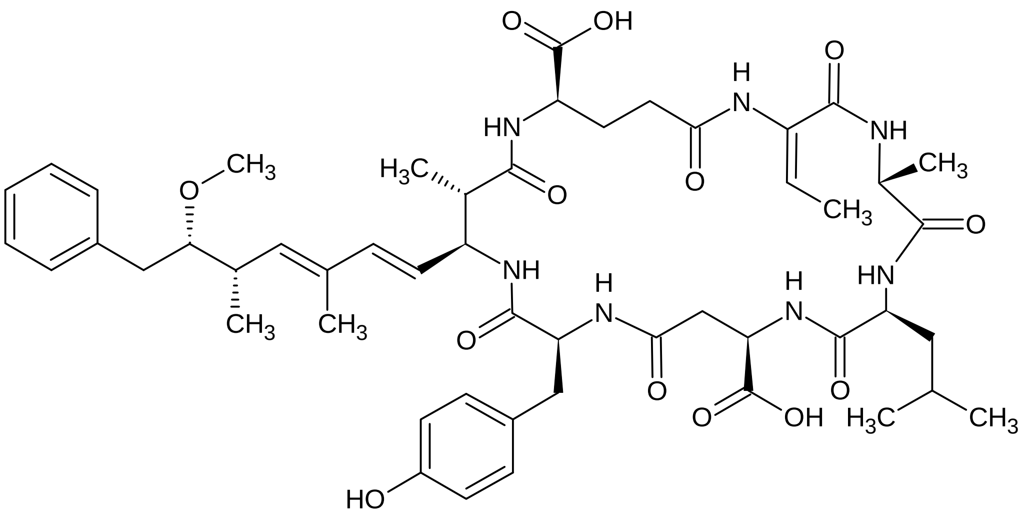

D-Ala | Leu | D-Asp | Tyr | Adda | D-Glu | Dhb

Formula (M)	Ion	Meas. m/z	Pred. m/z	Diff (mDa)	Diff (ppm)	Iso Score
C51 H69 N7 O13	[M+H]+	988.5052	988.5026	2.6	2.63	80.53

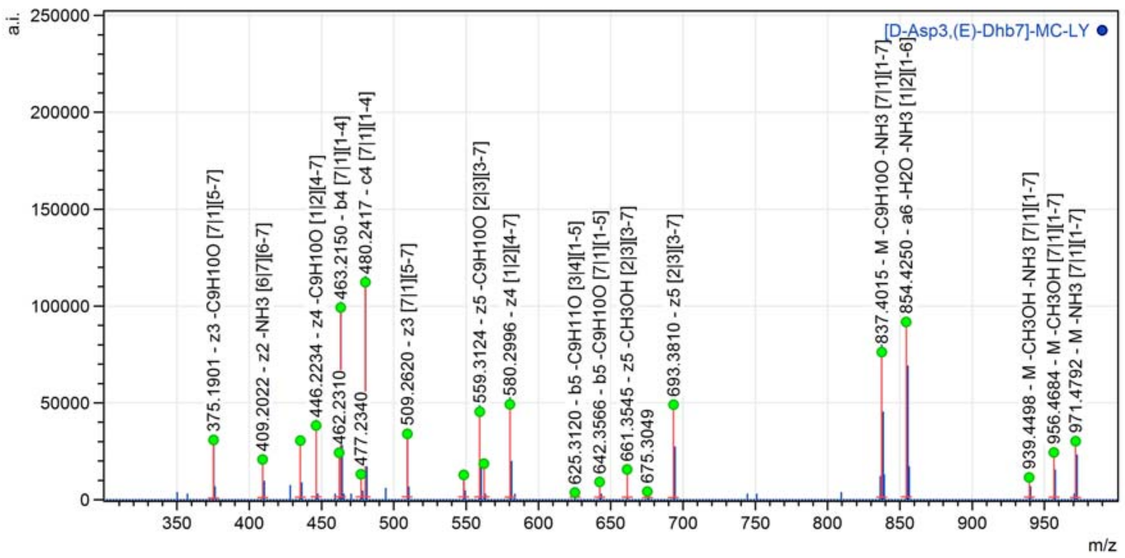

Length	Mo. Mass	Av. Mass	Coverage	Matched Int.
7 (Cyclic)	987.4953	988.1349	100.0 %	100.0 %

Meas. m/z	Calc. m/z	δ (Da)	δ (ppm)	Rel. Int. (%)	Annotation	Formula
375.1901	375.1914	-0.0013	-3.6	27.07	z3 -C9H10O [7|1][5-7]	C20H26N2O5
375.1901	375.1914	-0.0013	-3.6	27.07	b3 -C9H10O -NH3 [4|5][1-3]	C20H26N2O5
409.2022	409.2010	0.0012	3.0	17.69	z2 -NH3 [6|7][6-7]	C25H28O5
435.2201	435.2238	-0.0038	-8.6	26.35	a4 [7|1][1-4]	C21H30N4O6
446.2234	446.2286	-0.0052	-11.6	33.35	z4 -C9H10O [1|2][4-7]	C23H31N3O6
446.2234	446.2286	-0.0052	-11.6	33.35	b4 -C9H10O -NH3 [4|5][1-4]	C23H31N3O6
462.2310	462.2347	-0.0037	-8.1	20.60	c4 -H2O [7|1][1-4]	C22H31N5O6

463.2150	463.2187	-0.0037	-7.9	88.17	b4 [7|1][1-4]	C22H30N4O7
477.2340	477.2384	-0.0044	-9.2	10.62	z3 -CH3OH [7|1][5-7]	C28H32N2O5
477.2340	477.2384	-0.0044	-9.2	10.62	b3 -CH3OH -NH3 [4|5][1-3]	C28H32N2O5
480.2417	480.2453	-0.0036	-7.5	100.00	c4 [7|1][1-4]	C22H33N5O7
509.2620	509.2646	-0.0026	-5.1	29.31	b3 -NH3 [4|5][1-3]	C29H36N2O6
509.2620	509.2646	-0.0026	-5.1	29.31	z3 [7|1][5-7]	C29H36N2O6
548.2773	548.2755	0.0018	3.2	10.30	b4 -CH3OH -NH3 [4|5][1-4]	C31H37N3O6
548.2773	548.2755	0.0018	3.2	10.30	z4 -CH3OH [1|2][4-7]	C31H37N3O6
559.3124	559.3126	-0.0002	-0.4	39.71	b5 -C9H10O -NH3 [4|5][1-5]	C29H42N4O7
559.3124	559.3126	-0.0002	-0.4	39.71	z5 -C9H10O [2|3][3-7]	C29H42N4O7
562.2838	562.2912	-0.0073	-13.0	15.55	b4 -H2O -NH3 [4|5][1-4]	C32H39N3O6
562.2838	562.2912	-0.0073	-13.0	15.55	z4 -H2O [1|2][4-7]	C32H39N3O6
580.2996	580.3017	-0.0021	-3.6	43.14	z4 [1|2][4-7]	C32H41N3O7
580.2996	580.3017	-0.0021	-3.6	43.14	b4 -NH3 [4|5][1-4]	C32H41N3O7
625.3120	625.3106	0.0014	2.2	2.12	b5 -C9H11O [3|4][1-5]	C32H42N5O8
642.3566	642.3538	0.0029	4.5	7.04	a4 -H2O -NH3 [1|2][1-4]	C38H47N3O6
642.3566	642.3497	0.0069	10.7	7.04	b5 -C9H10O [7|1][1-5]	C33H47N5O8
661.3545	661.3596	-0.0051	-7.7	13.00	z5 -CH3OH [2|3][3-7]	C37H48N4O7
661.3545	661.3596	-0.0051	-7.7	13.00	a4 [3|4][1-4]	C37H48N4O7
661.3545	661.3596	-0.0051	-7.7	13.00	b5 -CH3OH -NH3 [4|5][1-5]	C37H48N4O7
675.3049	675.2984	0.0065	9.6	2.75	b6 [5|6][1-6]	C31H42N6O11
693.3810	693.3858	-0.0048	-6.9	43.17	b5 -NH3 [4|5][1-5]	C38H52N4O8
693.3810	693.3858	-0.0048	-6.9	43.17	z5 [2|3][3-7]	C38H52N4O8
693.3810	693.3732	0.0078	11.2	43.17	a6 -C9H11O -NH3 [3|4][1-6]	C37H50N5O8
837.4015	837.4029	-0.0014	-1.7	67.46	M -C9H10O -NH3 [7|1][1-7]	C42H56N6O12
854.4250	854.4335	-0.0084	-9.9	81.46	a6 -H2O -NH3 [1|2][1-6]	C47H59N5O10
854.4250	854.4294	-0.0044	-5.2	81.46	M -C9H10O [7|1][1-7]	C42H59N7O12
939.4498	939.4498	-0.0000	-0.0	9.22	M -CH3OH -NH3 [7|1][1-7]	C50H62N6O12
956.4684	956.4764	-0.0080	-8.4	20.80	M -CH3OH [7|1][1-7]	C50H65N7O12
971.4792	971.4761	0.0031	3.2	26.17	M -NH3 [7|1][1-7]	C51H66N6O13

NMR

See Niedermeyer et al., Nat. Prod. Bioprospect., 2014, accepted

[D-Asp^{3},(*E*)-Dhb^{7}]-MC-LW (9)

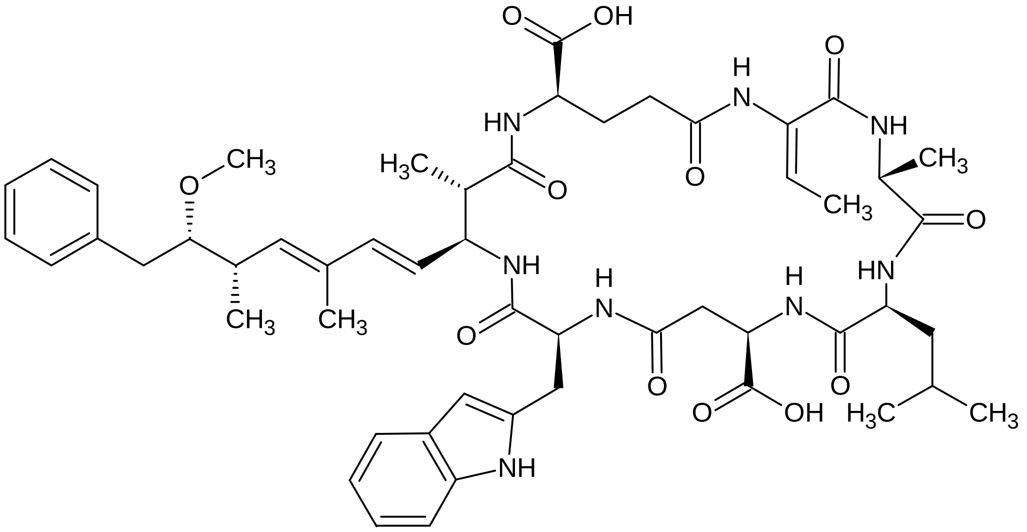

D-Ala | Leu | D-Asp | Trp | Adda | D-Glu | Dhb

Formula (M)	Ion	Meas. m/z	Pred. m/z	Diff (mDa)	Diff (ppm)	Iso Score
C53 H70 N8 O12	[M+H]+	1011.5172	1011.5186	-1.4	-1.38	79.91

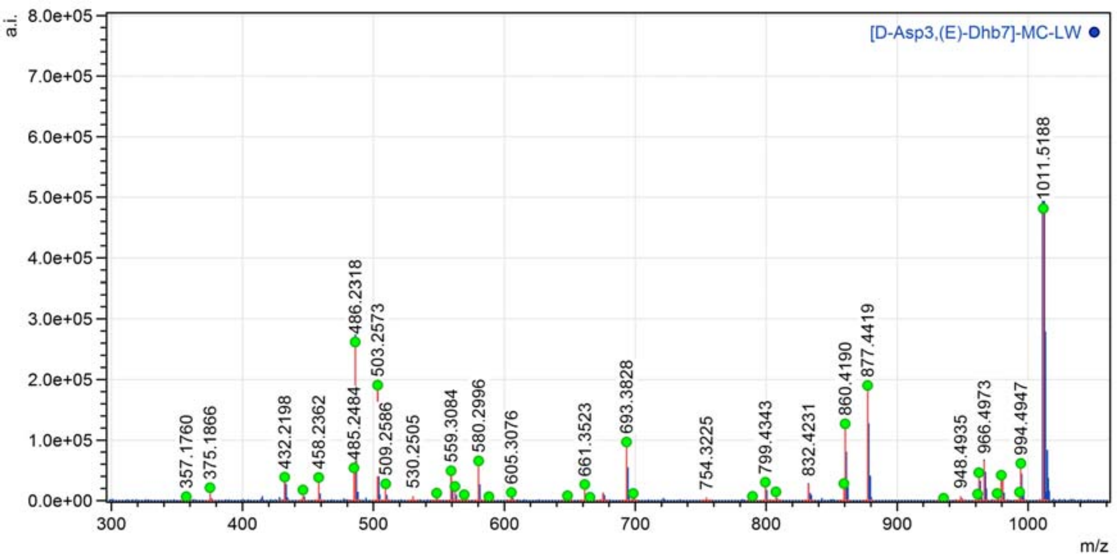

**Length**	**Mo. Mass**	**Av. Mass**	**Coverage**	**Matched Int.**
7 (Cyclic)	1010.5113	1011.1717	100.0 %	90.4 %

Meas. m/z	Calc. m/z	δ (Da)	δ (ppm)	Rel. Int. (%)	Annotation	Formula
357.1760	357.1809	-0.0048	-13.5	1.35	z3 -C9H10O -H2O [7|1][5-7]	C20H24N2O4
375.1866	375.1914	-0.0049	-13.0	4.42	z3 -C9H10O [7|1][5-7]	C20H26N2O5
432.2198	432.2241	-0.0044	-10.1	8.02	c3 [1|2][1-3]	C21H29N5O5
446.2263	446.2286	-0.0022	-5.0	3.63	z4 -C9H10O [1|2][4-7]	C23H31N3O6
458.2362	458.2398	-0.0036	-7.8	7.94	a4 [7|1][1-4]	C23H31N5O5
486.2318	486.2347	-0.0029	-5.9	54.38	b4 [7|1][1-4]	C24H31N5O6

503.2573	503.2613	-0.0040	-7.9	39.55	c4 [7|1][1-4]	C24H34N6O6
509.2586	509.2646	-0.0060	-11.7	5.64	z3 [7|1][5-7]	C29H36N2O6
509.2586	509.2646	-0.0060	-11.7	5.64	b3 -NH3 [4|5][1-3]	C29H36N2O6
548.2727	548.2755	-0.0029	-5.2	2.52	z4 -CH3OH [1|2][4-7]	C31H37N3O6
548.2727	548.2755	-0.0029	-5.2	2.52	b4 -CH3OH -NH3 [4|5][1-4]	C31H37N3O6
559.3084	559.3126	-0.0043	-7.6	10.17	b5 -C9H10O -NH3 [4|5][1-5]	C29H42N4O7
559.3084	559.3126	-0.0043	-7.6	10.17	z5 -C9H10O [2|3][3-7]	C29H42N4O7
562.2890	562.2912	-0.0022	-3.9	4.76	b4 -H2O -NH3 [4|5][1-4]	C32H39N3O6
562.2890	562.2912	-0.0022	-3.9	4.76	z4 -H2O [1|2][4-7]	C32H39N3O6
569.2633	569.2718	-0.0085	-15.0	1.96	b5 [6|7][1-5]	C28H36N6O7
580.2996	580.3017	-0.0022	-3.7	13.54	b4 -NH3 [4|5][1-4]	C32H41N3O7
580.2996	580.3017	-0.0022	-3.7	13.54	z4 [1|2][4-7]	C32H41N3O7
588.2876	588.2817	0.0059	10.0	1.29	b4 +CO -C9H10O -H2O [3|4][1-4]	C32H37N5O6
605.3076	605.3082	-0.0006	-0.9	2.71	c4 +CO -C9H10O -H2O [3|4][1-4]	C32H40N6O6
648.3367	648.3392	-0.0025	-3.9	1.60	b5 -C9H10O -NH3 [7|1][1-5]	C35H45N5O7
648.3367	648.3392	-0.0025	-3.9	1.60	z5 -C9H10O [5|6][3-7]	C35H45N5O7
661.3523	661.3596	-0.0073	-11.0	5.42	b5 -CH3OH -NH3 [4|5][1-5]	C37H48N4O7
661.3523	661.3596	-0.0073	-11.0	5.42	z5 -CH3OH [2|3][3-7]	C37H48N4O7
665.3734	665.3697	0.0037	5.6	1.03	a4 -H2O -NH3 [1|2][1-4]	C40H48N4O5
665.3734	665.3657	0.0077	11.6	1.03	b5 -C9H10O [7|1][1-5]	C35H48N6O7
693.3828	693.3858	-0.0030	-4.3	20.07	z5 [2|3][3-7]	C38H52N4O8
693.3828	693.3858	-0.0030	-4.3	20.07	b5 -NH3 [4|5][1-5]	C38H52N4O8
698.3109	698.3144	-0.0035	-5.1	2.34	b6 [5|6][1-6]	C33H43N7O10
789.4081	789.4056	0.0025	3.2	1.36	b6 +CO -C9H11O [3|4][1-6]	C41H54N7O9
799.4343	799.4389	-0.0046	-5.8	6.15	b5 [7|1][1-5]	C44H58N6O8
807.4200	807.4287	-0.0087	-10.8	2.86	b6 -H2O [4|5][1-6]	C42H58N6O10
859.4343	859.4349	-0.0006	-0.7	5.71	M -C9H10O -H2O [7|1][1-7]	C44H58N8O10
860.4190	860.4189	0.0001	0.1	26.26	M -C9H10O -NH3 [7|1][1-7]	C44H57N7O11
877.4419	877.4495	-0.0076	-8.7	39.41	a6 -H2O -NH3 [1|2][1-6]	C49H60N6O9
877.4419	877.4454	-0.0036	-4.1	39.41	M -C9H10O [7|1][1-7]	C44H60N8O11
935.4651	935.4662	-0.0010	-1.1	0.71	c6 +CO -CH3OH -H2O [1|2][1-6]	C50H62N8O10
961.4832	961.4818	0.0014	1.4	2.09	M -CH3OH -H2O [7|1][1-7]	C52H64N8O10
962.4716	962.4658	0.0058	6.0	9.51	M -CH3OH -NH3 [7|1][1-7]	C52H63N7O11
976.4822	976.4815	0.0007	0.7	2.34	M -H2O -NH3 [7|1][1-7]	C53H65N7O11
979.4930	979.4924	0.0006	0.6	8.60	M -CH3OH [7|1][1-7]	C52H66N8O11
993.5082	993.5080	0.0002	0.2	2.82	M -H2O [7|1][1-7]	C53H68N8O11
994.4947	994.4920	0.0026	2.6	12.67	M -NH3 [7|1][1-7]	C53H67N7O12
1011.5188	1011.5186	0.0002	0.2	100.00	M [7|1][1-7]	C53H70N8O12

NMR

See Niedermeyer et al., Nat. Prod. Bioprospect., 2014, accepted

[D-Asp^{3},(*E*)-Dhb^{7}]MC-RR (11)

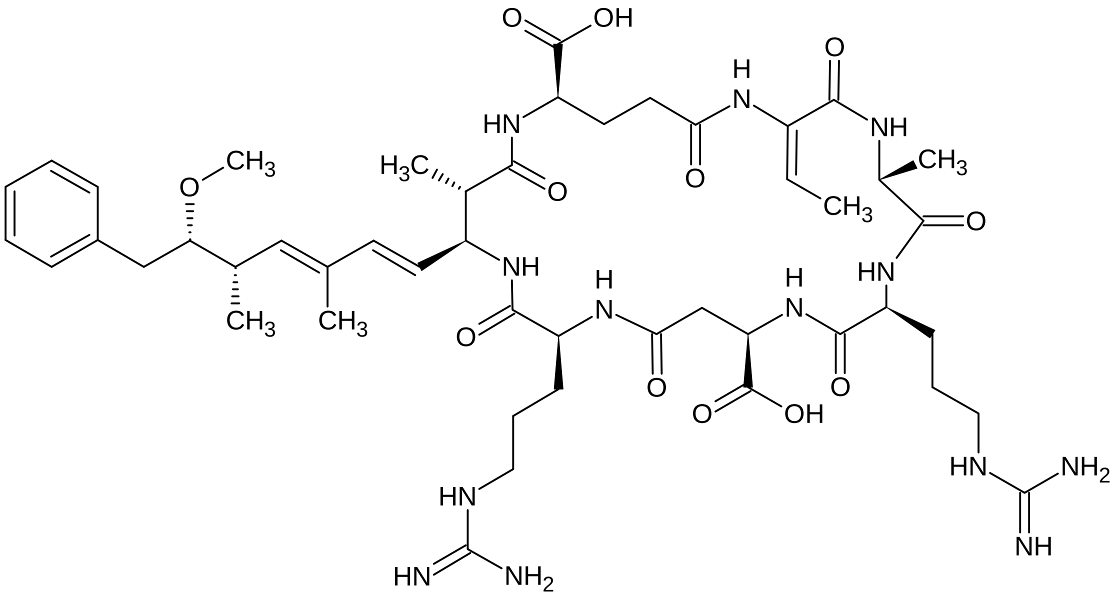

D-Ala | Arg | D-Asp | Arg | Adda | D-Glu | Dhb

Formula (M)	Ion	Meas. m/z	Pred. m/z	Diff (mDa)	Diff (ppm)	Iso Score
C48 H73 N13 O12	[M+2H]2+	512.7833	512.7824	0.9	1.76	53.49

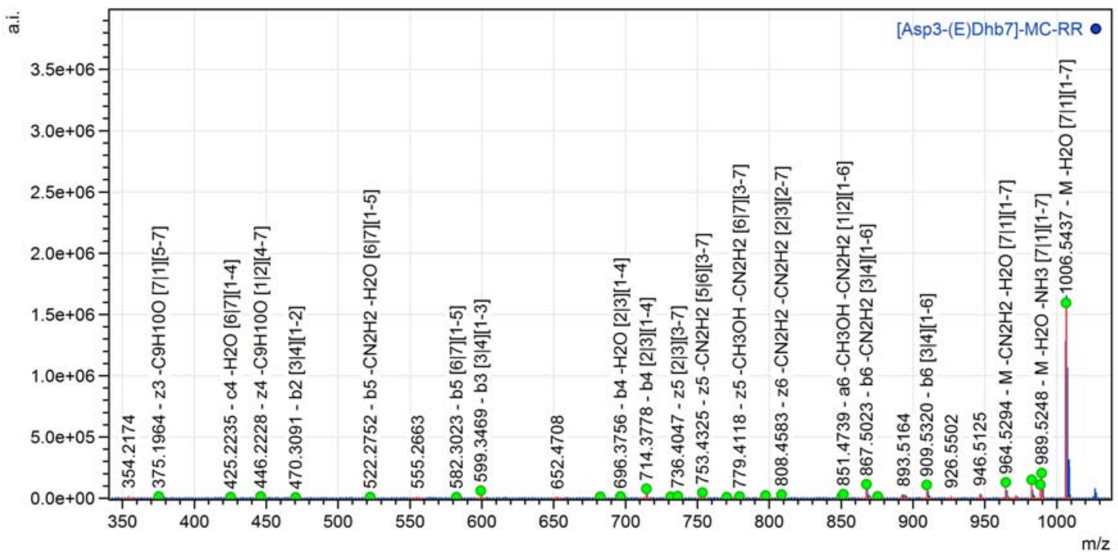

Length	Mo. Mass	Av. Mass	Coverage	Matched Int.
7 (Cyclic)	1023.5502	1024.1755	100.0 %	94.9 %

Meas. m/z	Calc. m/z	δ (Da)	δ (ppm)	Rel. Int. (%)	Annotation	Formula
375.1964	375.1914	0.0049	13.2	0.97	z3 -C9H10O [7|1][5-7]	C20H26N2O5
425.2235	425.2255	-0.0020	-4.8	0.57	c4 -H2O [6|7][1-4]	C17H28N8O5
446.2228	446.2286	-0.0058	-13.0	0.90	z4 -C9H10O [1|2][4-7]	C23H31N3O6
470.3091	470.3126	-0.0034	-7.3	0.47	b2 [3|4][1-2]	C26H39N5O3

522.2752	522.2783	-0.0031	-5.9	0.59	b5 -CN2H2 -H2O [6|7][1-5]	C22H35N9O6
582.3023	582.3107	-0.0084	-14.4	0.61	b5 [6|7][1-5]	C23H39N11O7
599.3469	599.3552	-0.0083	-13.8	3.91	b3 [3|4][1-3]	C31H46N6O6
682.3869	682.3923	-0.0054	-7.9	0.83	b4 [3|4][1-4]	C35H51N7O7
682.3869	682.3923	-0.0054	-7.9	0.83	b4 -CH5N3 [1|2][1-4]	C35H51N7O7
682.3869	682.3923	-0.0054	-7.9	0.83	b4 -CN2H2 -NH3 [1|2][1-4]	C35H51N7O7
682.3869	682.3923	-0.0054	-7.9	0.83	z4 -CN2H2 [5|6][4-7]	C35H51N7O7
696.3756	696.3715	0.0041	5.9	0.88	b4 -H2O [2|3][1-4]	C35H49N7O8
714.3778	714.3821	-0.0043	-6.0	4.90	b4 [2|3][1-4]	C35H51N7O9
731.4083	731.4087	-0.0004	-0.5	0.67	c4 [2|3][1-4]	C35H54N8O9
736.4047	736.4100	-0.0054	-7.3	1.03	b5 -C9H10O [1|2][1-5]	C32H53N11O9
736.4047	736.4028	0.0018	2.5	1.03	z5 -CN2H2 -NH3 [5|6][3-7]	C38H53N7O8
736.4047	736.4028	0.0018	2.5	1.03	z5 -CH5N3 [5|6][3-7]	C38H53N7O8
736.4047	736.4028	0.0018	2.5	1.03	b5 -NH3 [4|5][1-5]	C38H53N7O8
736.4047	736.4028	0.0018	2.5	1.03	b5 -CH5N3 -NH3 [7|1][1-5]	C38H53N7O8
736.4047	736.4028	0.0018	2.5	1.03	z5 [1|2][3-7]	C38H53N7O8
736.4047	736.4028	0.0018	2.5	1.03	z5 [2|3][3-7]	C38H53N7O8
736.4047	736.4028	0.0018	2.5	1.03	b5 -NH3 [3|4][1-5]	C38H53N7O8
753.4325	753.4294	0.0031	4.1	2.94	b5 [4|5][1-5]	C38H56N8O8
753.4325	753.4294	0.0031	4.1	2.94	b5 [3|4][1-5]	C38H56N8O8
753.4325	753.4294	0.0031	4.1	2.94	z5 -CN2H2 [5|6][3-7]	C38H56N8O8
753.4325	753.4294	0.0031	4.1	2.94	b5 -CN2H2 -NH3 [7|1][1-5]	C38H56N8O8
753.4325	753.4294	0.0031	4.1	2.94	b5 -CH5N3 [7|1][1-5]	C38H56N8O8
770.4504	770.4559	-0.0055	-7.2	0.64	b5 -CN2H2 [7|1][1-5]	C38H59N9O8
779.4118	779.4087	0.0032	4.1	0.87	b5 -H2O [2|3][1-5]	C39H54N8O9
779.4118	779.4087	0.0032	4.1	0.87	z5 -CH3OH -CN2H2 [6|7][3-7]	C39H54N8O9
779.4118	779.4087	0.0032	4.1	0.87	b5 -CH3OH -CH5N3 [1|2][1-5]	C39H54N8O9
797.4095	797.4192	-0.0097	-12.1	1.33	b5 [2|3][1-5]	C39H56N8O10
808.4583	808.4604	-0.0020	-2.5	1.88	z6 -CN2H2 -CN2H2 [2|3][2-7]	C42H61N7O9
808.4583	808.4604	-0.0020	-2.5	1.88	b6 -CH5N3 -CN2H2 [3|4][1-6]	C42H61N7O9
850.4457	850.4458	-0.0001	-0.1	0.93	z6 -CH3OH -CN2H2 [6|7][2-7]	C42H59N9O10
850.4457	850.4458	-0.0001	-0.1	0.93	b6 -H2O [4|5][1-6]	C42H59N9O10
850.4457	850.4458	-0.0001	-0.1	0.93	b6 -CH3OH -CH5N3 [7|1][1-6]	C42H59N9O10
850.4457	850.4458	-0.0001	-0.1	0.93	b6 -H2O [2|3][1-6]	C42H59N9O10
851.4739	851.4774	-0.0035	-4.2	2.10	a6 -CH3OH -CN2H2 [1|2][1-6]	C42H62N10O9
867.5023	867.5087	-0.0064	-7.3	7.14	b6 -CN2H2 [3|4][1-6]	C43H66N10O9
875.4906	875.4886	0.0020	2.2	0.83	a6 -CH3OH -H2O [1|2][1-6]	C43H62N12O8
909.5320	909.5305	0.0015	1.6	6.77	b6 [3|4][1-6]	C44H68N12O9
964.5294	964.5251	0.0044	4.5	8.15	M -CN2H2 -H2O [7|1][1-7]	C47H69N11O11
982.5303	982.5356	-0.0053	-5.4	9.40	M -CN2H2 [7|1][1-7]	C47H71N11O12
988.5309	988.5363	-0.0054	-5.4	6.97	M -H2O -H2O [7|1][1-7]	C48H69N13O10
989.5248	989.5203	0.0045	4.5	12.84	M -H2O -NH3 [7|1][1-7]	C48H68N12O11
1006.5437	1006.5469	-0.0032	-3.2	100.00	M -H2O [7|1][1-7]	C48H71N13O11

NMR

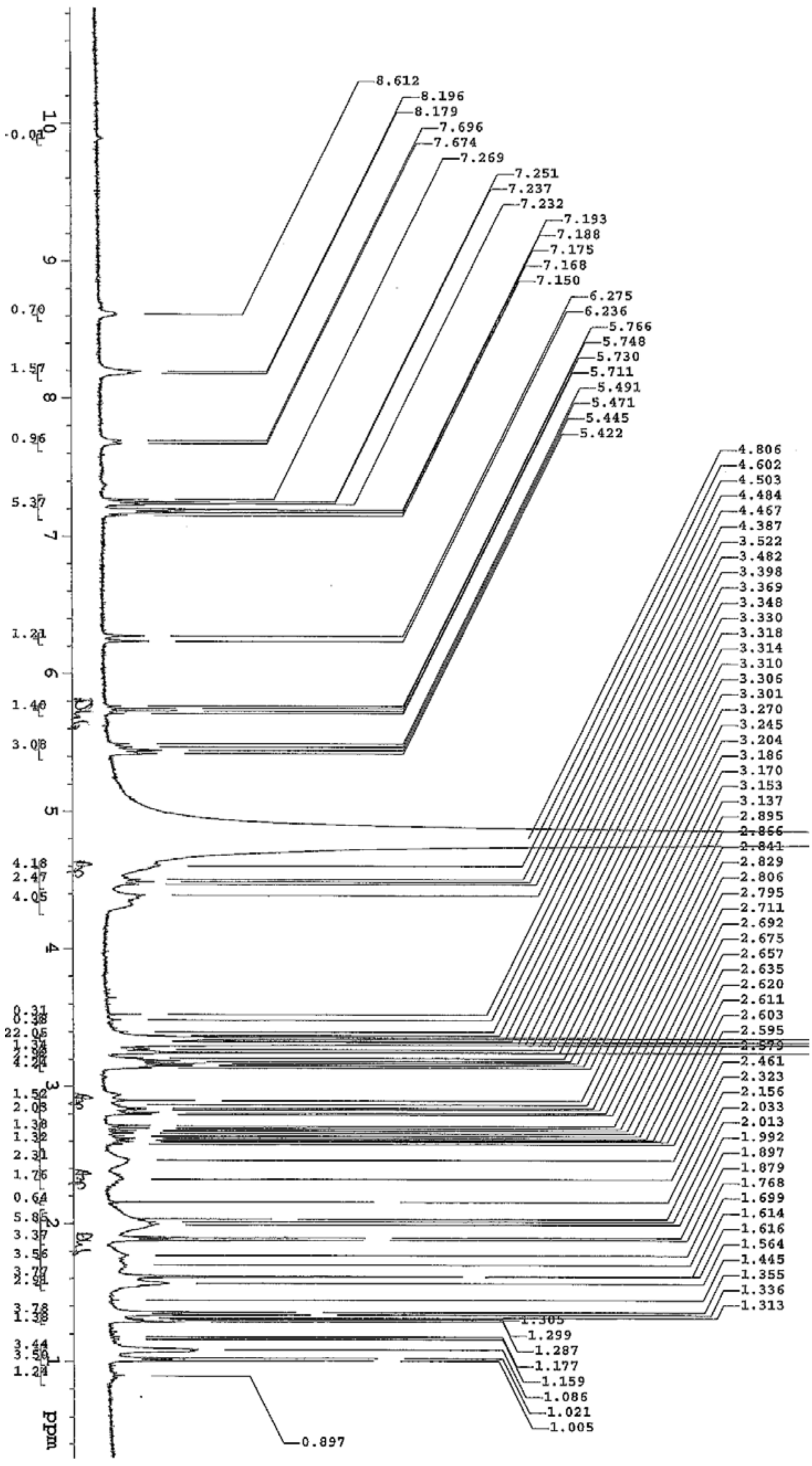
8.612
8.196
8.179
7.696
7.674
7.269
7.251
7.237
7.232
7.193
7.188
7.175
7.168
7.150
6.275
6.236
5.766
5.748
5.730
5.711
5.491
5.471
5.445
5.422
4.806
4.602
4.503
4.484
4.467
4.387
3.522
3.482
3.398
3.369
3.348
3.330
3.318
3.314
3.310
3.306
3.301
3.270
3.245
3.204
3.186
3.170
3.153
3.137
2.895
2.866
2.841
2.829
2.806
2.795
2.711
2.692
2.675
2.657
2.635
2.620
2.611
2.603
2.595
2.461
2.323
2.156
2.033
2.013
1.992
1.897
1.879
1.768
1.699
1.614
1.616
1.564
1.445
1.355
1.336
1.313
1.305
1.299
1.287
1.177
1.159
1.086
1.021
1.005
0.897
10
9
8
7
6
5
4
3
2
1
ppm

MC-RF (12)

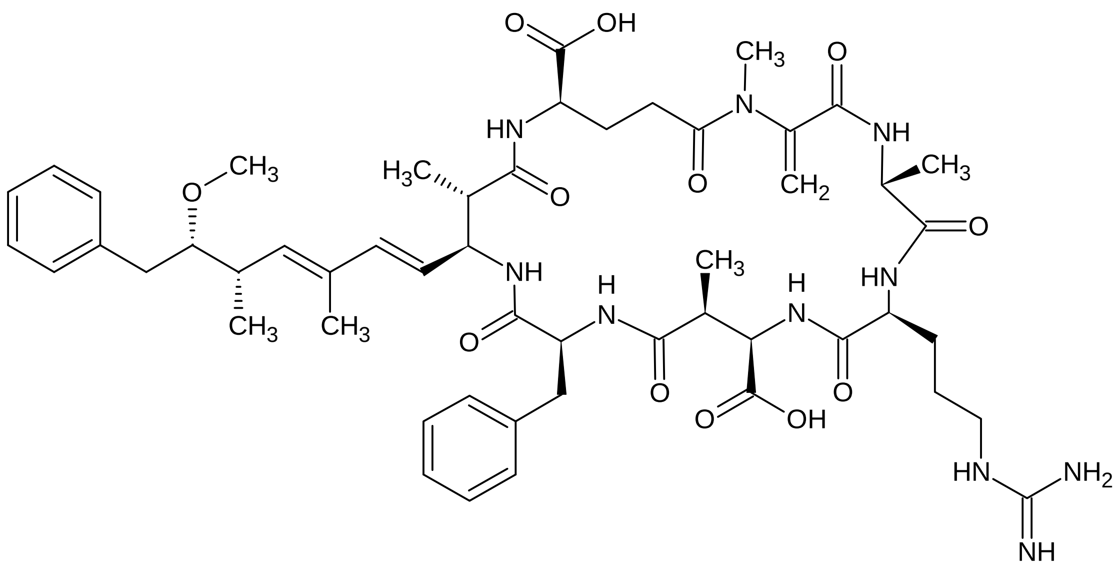

D-Ala | Arg | D-MeAsp | Phe | Adda | D-Glu | Mdha

Formula (M)	Ion	Meas. m/z	Pred. m/z	Diff (mDa)	Diff (ppm)	Iso Score
C52 H72 N10 O12	[M+H]+	1029.5407	1029.5404	0.3	0.29	74.08

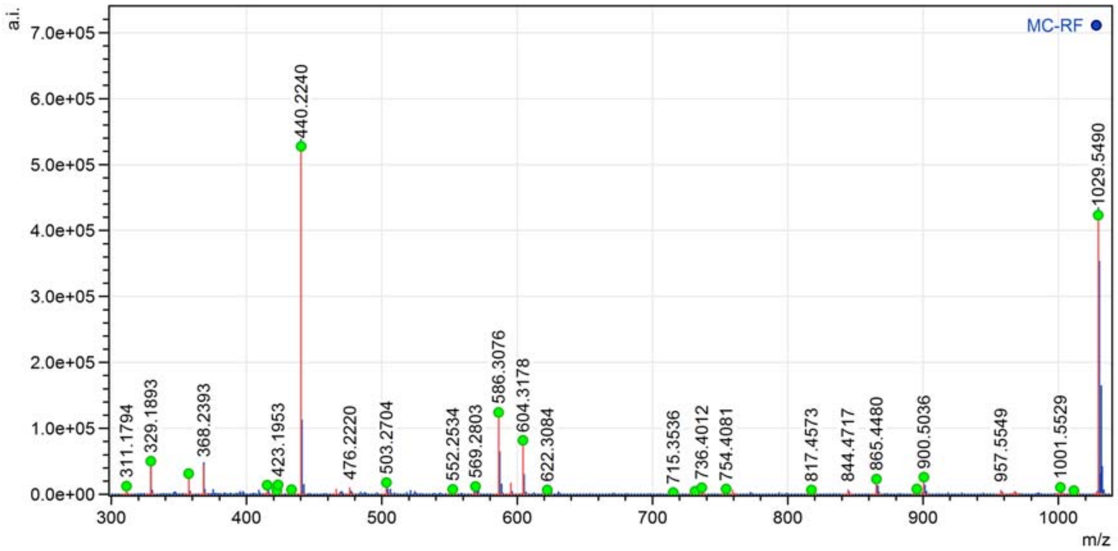

Length	Mo. Mass	Av. Mass	Coverage	Matched Int.
7 (Cyclic)	1028.5331	1029.1903	100.0 %	93.3 %

Meas. m/z	Calc. m/z	δ (Da)	δ (ppm)	Rel. Int. (%)	Annotation	Formula
311.1794	311.1826	-0.0033	-10.5	2.37	b3 [6|7][1-3]	C13H22N6O3
311.1794	311.1826	-0.0033	-10.5	2.37	a3 -H2O [7|1][1-3]	C13H22N6O3
329.1893	329.1932	-0.0039	-11.9	9.53	a3 [7|1][1-3]	C13H24N6O4
357.1849	357.1881	-0.0032	-9.0	5.95	b3 [7|1][1-3]	C14H24N6O5
415.2086	415.2088	-0.0003	-0.7	2.57	b3 -H2O [1|2][1-3]	C20H26N6O4

422.2149	422.2146	0.0003	0.7	1.17	b4 -H2O [5|6][1-4]	C18H27N7O5
422.2149	422.2146	0.0003	0.7	1.17	b4 -H2O [6|7][1-4]	C18H27N7O5
423.1953	423.1987	-0.0033	-7.8	2.70	z4 [2|3][4-7]	C18H26N6O6
423.1953	423.1987	-0.0033	-7.8	2.70	b4 -NH3 [6|7][1-4]	C18H26N6O6
423.1953	423.1987	-0.0033	-7.8	2.70	b4 -NH3 [5|6][1-4]	C18H26N6O6
423.1953	423.1987	-0.0033	-7.8	2.70	z4 [3|4][4-7]	C18H26N6O6
433.2224	433.2194	0.0030	6.9	1.33	b3 [1|2][1-3]	C20H28N6O5
440.2240	440.2252	-0.0012	-2.7	100.00	b4 [5|6][1-4]	C18H29N7O6
440.2240	440.2252	-0.0012	-2.7	100.00	b4 [6|7][1-4]	C18H29N7O6
503.2704	503.2725	-0.0021	-4.1	3.30	c4 -H2O [7|1][1-4]	C23H34N8O5
552.2534	552.2565	-0.0031	-5.6	1.37	b5 -H2O -NH3 [6|7][1-5]	C27H33N7O6
552.2534	552.2565	-0.0031	-5.6	1.37	z5 -H2O [4|5][3-7]	C27H33N7O6
569.2803	569.2831	-0.0028	-4.9	2.23	b5 -H2O [6|7][1-5]	C27H36N8O6
586.3076	586.3096	-0.0020	-3.3	23.51	c5 -H2O [6|7][1-5]	C27H39N9O6
604.3178	604.3202	-0.0024	-3.9	15.49	c5 [6|7][1-5]	C27H41N9O7
622.3084	622.2997	0.0087	14.0	1.23	z5 -C9H11O -CO [7|1][3-7]	C33H41N4O8
622.3084	622.2997	0.0087	14.0	1.23	a5 -C9H11O -NH3 [2|3][1-5]	C33H41N4O8
715.3536	715.3522	0.0014	1.9	0.55	c6 -H2O [5|6][1-6]	C32H46N10O9
731.3750	731.3723	0.0027	3.8	0.84	b6 -C9H10O -NH3 [4|5][1-6]	C34H50N8O10
731.3750	731.3723	0.0027	3.8	0.84	z6 -C9H10O [3|4][2-7]	C34H50N8O10
736.4012	736.4028	-0.0016	-2.2	1.91	z5 [2|3][3-7]	C38H53N7O8
736.4012	736.4028	-0.0016	-2.2	1.91	b5 -NH3 [4|5][1-5]	C38H53N7O8
754.4081	754.4134	-0.0053	-7.1	1.52	a6 -C9H10O -CN2H2 [1|2][1-6]	C38H55N7O9
817.4573	817.4607	-0.0034	-4.1	1.22	b5 [7|1][1-5]	C43H60N8O8
817.4573	817.4495	0.0078	9.6	1.22	a6 -CO [2|3][1-6]	C44H60N6O9
865.4480	865.4454	0.0026	3.0	4.39	z6 [3|4][2-7]	C43H60N8O11
865.4480	865.4454	0.0026	3.0	4.39	b6 -NH3 [4|5][1-6]	C43H60N8O11
895.4691	895.4713	-0.0022	-2.4	1.49	a6 -H2O -NH3 [1|2][1-6]	C48H62N8O9
895.4691	895.4713	-0.0022	-2.4	1.49	z6 -CO -H2O [7|1][2-7]	C48H62N8O9
895.4691	895.4713	-0.0022	-2.4	1.49	z6 -H2O -CO [7|1][2-7]	C48H62N8O9
895.4691	895.4672	0.0018	2.0	1.49	M -C9H10O [7|1][1-7]	C43H62N10O11
900.5036	900.4978	0.0058	6.5	4.87	b6 -CO -H2O [7|1][1-6]	C47H65N9O9
900.5036	900.4978	0.0058	6.5	4.87	a6 -H2O [7|1][1-6]	C47H65N9O9
900.5036	900.4978	0.0058	6.5	4.87	b6 [6|7][1-6]	C47H65N9O9
900.5036	900.4978	0.0058	6.5	4.87	b6 -H2O -CO [7|1][1-6]	C47H65N9O9
900.5036	900.4978	0.0058	6.5	4.87	b6 [3|4][1-6]	C47H65N9O9
1001.5529	1001.5455	0.0075	7.4	1.93	M -CO [7|1][1-7]	C51H72N10O11
1011.5256	1011.5298	-0.0042	-4.1	1.04	M -H2O [7|1][1-7]	C52H70N10O11
1029.5490	1029.5404	0.0086	8.4	80.24	M [7|1][1-7]	C52H72N10O12

NMR

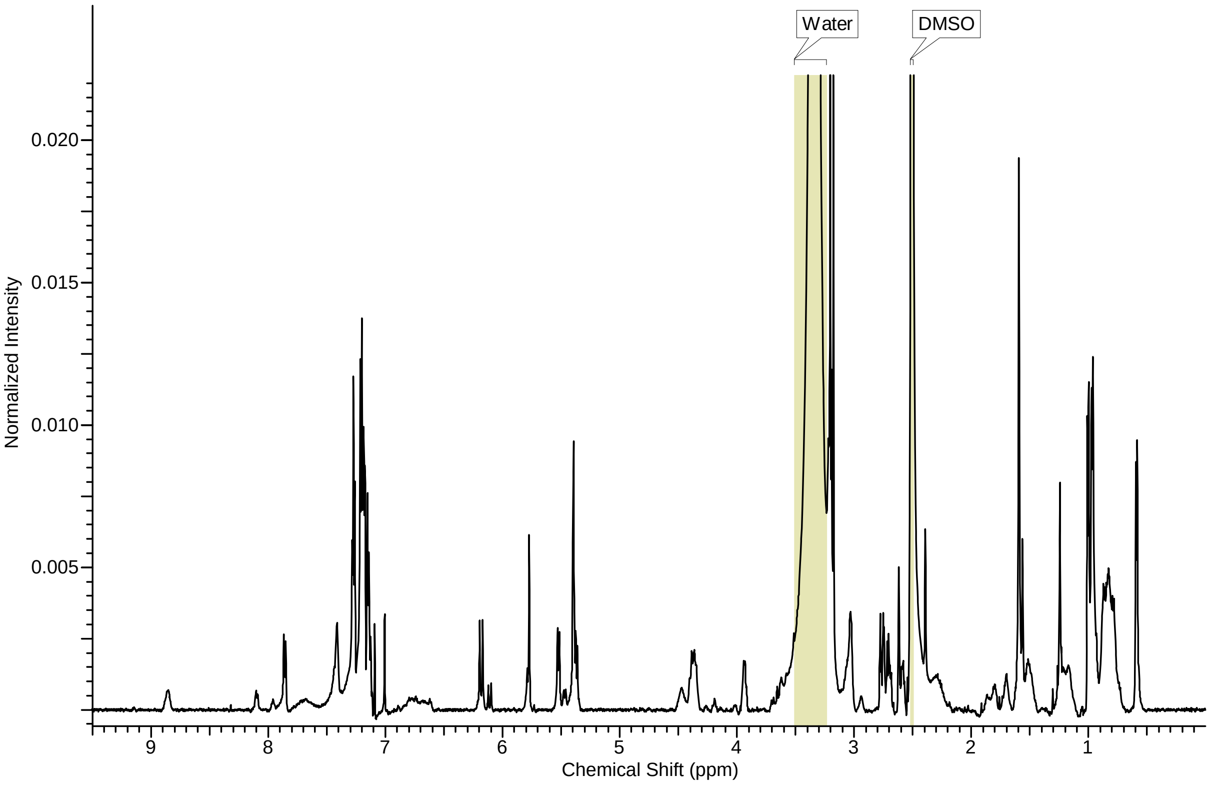

^{1}H-NMR spectrum agrees with the postulated structure.

[D-Asp[3]]MC-YR (14)

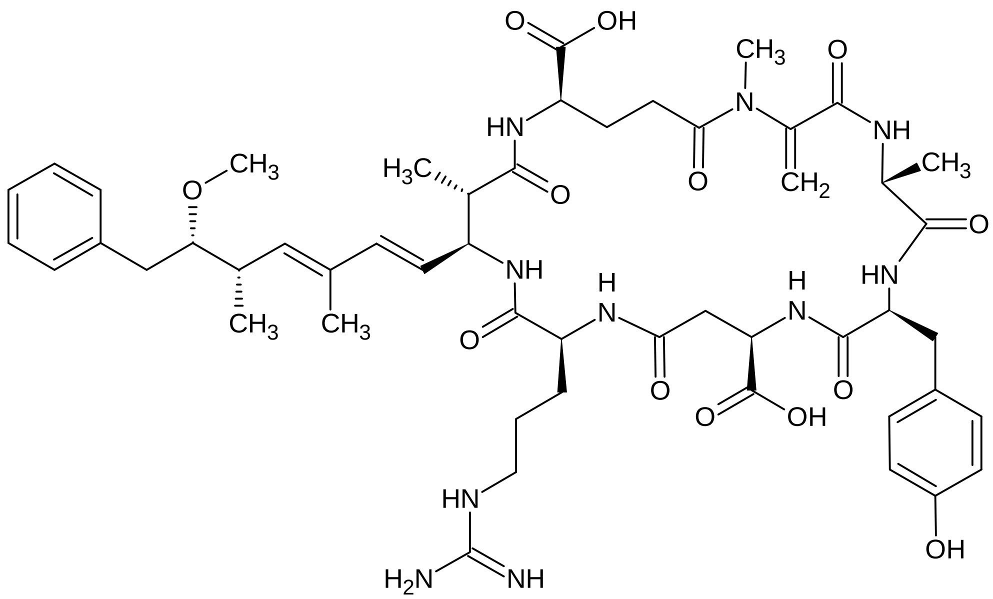

D-Ala | Tyr | D-Asp | Arg | Adda | D-Glu | Mdha

Formula (M)	Ion	Meas. m/z	Pred. m/z	Diff (mDa)	Diff (ppm)	Iso Score
C51 H70 N10 O13	[M+H]+	1031.5189	1031.5197	-0.8	-0.78	100.00

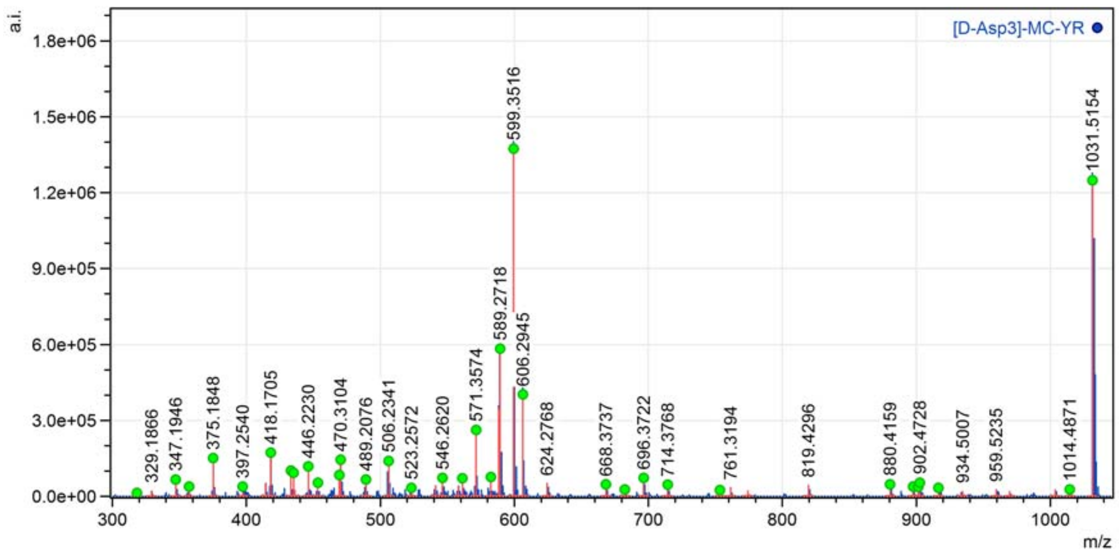

Length	Mo. Mass	Av. Mass	Coverage	Matched Int.
7 (Cyclic)	1030.5124	1031.1631	100.0 %	94.2 %

Meas. m/z	Calc. m/z	δ (Da)	δ (ppm)	Rel. Int. (%)	Annotation	Formula
318.1432	318.1448	-0.0016	-5.0	0.91	b3 [6|7][1-3]	C16H19N3O4
347.1946	347.1965	-0.0019	-5.5	4.77	a3 -C9H10O -NH3 [4|5][1-3]	C19H26N2O4
357.1586	357.1571	0.0015	4.3	2.70	z3 -C9H11O -NH3 [7|1][5-7]	C20H22NO5
375.1848	375.1914	-0.0066	-17.6	10.86	z3 -C9H10O [7|1][5-7]	C20H26N2O5

397.2540	397.2486	0.0055	13.7	2.66	a2 -H2O [4|5][1-2]	C24H32N2O3
418.1705	418.1721	-0.0016	-3.9	12.46	z3 [4|5][5-7]	C19H23N5O6
418.1705	418.1721	-0.0016	-3.9	12.46	b3 -NH3 [1|2][1-3]	C19H23N5O6
433.1673	433.1718	-0.0045	-10.3	7.26	b4 [6|7][1-4]	C20H24N4O7
435.1964	435.1987	-0.0023	-5.3	6.64	b3 [1|2][1-3]	C19H26N6O6
446.2230	446.2286	-0.0055	-12.4	8.44	z4 -C9H10O [1|2][4-7]	C23H31N3O6
446.2230	446.2286	-0.0055	-12.4	8.44	b4 -C9H10O -NH3 [4|5][1-4]	C23H31N3O6
453.2838	453.2860	-0.0022	-4.9	3.74	z2 [5|6][6-7]	C26H36N4O3
453.2838	453.2860	-0.0022	-4.9	3.74	b2 -NH3 [3|4][1-2]	C26H36N4O3
469.3241	469.3286	-0.0045	-9.5	5.94	c2 -H2O [3|4][1-2]	C26H40N6O2
470.3104	470.3126	-0.0022	-4.7	10.36	b2 [3|4][1-2]	C26H39N5O3
489.2076	489.2092	-0.0017	-3.4	4.68	z4 [4|5][4-7]	C22H28N6O7
489.2076	489.2092	-0.0017	-3.4	4.68	b4 -NH3 [7|1][1-4]	C22H28N6O7
506.2341	506.2358	-0.0016	-3.2	10.04	b4 [7|1][1-4]	C22H31N7O7
523.2572	523.2623	-0.0051	-9.7	2.22	c4 [7|1][1-4]	C22H34N8O7
546.2620	546.2558	0.0062	11.3	5.10	z4 -C9H10O -NH3 [6|7][4-7]	C26H35N5O8
561.2733	561.2780	-0.0046	-8.3	5.05	a5 [6|7][1-5]	C25H36N8O7
571.3574	571.3602	-0.0028	-4.9	18.91	a3 [3|4][1-3]	C30H46N6O5
582.3207	582.3286	-0.0079	-13.5	5.40	z3 [6|7][5-7]	C31H43N5O6
582.3207	582.3286	-0.0079	-13.5	5.40	b3 -NH3 [3|4][1-3]	C31H43N5O6
589.2718	589.2729	-0.0011	-1.8	42.35	b5 [6|7][1-5]	C26H36N8O8
599.3516	599.3552	-0.0036	-6.0	100.00	b3 [3|4][1-3]	C31H46N6O6
606.2945	606.2994	-0.0049	-8.1	29.14	c5 [6|7][1-5]	C26H39N9O8
668.3737	668.3766	-0.0029	-4.3	3.22	a4 -H2O [2|3][1-4]	C34H49N7O7
682.3965	682.3923	0.0042	6.1	1.82	b4 [3|4][1-4]	C35H51N7O7
696.3722	696.3756	-0.0033	-4.8	5.14	a5 -H2O -H2O [4|5][1-5]	C40H49N5O6
696.3722	696.3715	0.0007	1.0	5.14	b4 -H2O [2|3][1-4]	C35H49N7O8
714.3768	714.3861	-0.0094	-13.1	3.13	a5 -H2O [4|5][1-5]	C40H51N5O7
714.3768	714.3821	-0.0053	-7.5	3.13	b4 [2|3][1-4]	C35H51N7O9
714.3768	714.3695	0.0072	10.1	3.13	a5 -C9H11O [1|2][1-5]	C34H49N8O9
753.4238	753.4294	-0.0056	-7.4	1.59	b5 [3|4][1-5]	C38H56N8O8
753.4238	753.4168	0.0070	9.3	1.59	a6 -C9H11O [3|4][1-6]	C37H54N9O8
880.4159	880.4199	-0.0041	-4.6	3.29	M -C9H10O -NH3 [7|1][1-7]	C42H57N9O12
897.4444	897.4505	-0.0061	-6.8	2.65	a6 -H2O -NH3 [1|2][1-6]	C47H60N8O10
897.4444	897.4465	-0.0021	-2.3	2.65	M -C9H10O [7|1][1-7]	C42H60N10O12
901.4850	901.4931	-0.0080	-8.9	2.56	c6 -CH3OH [3|4][1-6]	C46H64N10O9
901.4850	901.4931	-0.0080	-8.9	2.56	c6 -H2O [6|7][1-6]	C46H64N10O9
902.4728	902.4771	-0.0043	-4.8	3.76	b6 [6|7][1-6]	C46H63N9O10
902.4728	902.4771	-0.0043	-4.8	3.76	a6 -H2O [7|1][1-6]	C46H63N9O10
902.4728	902.4658	0.0069	7.7	3.76	b6 +CO -CN2H2 [3|4][1-6]	C47H63N7O11
916.4861	916.4927	-0.0066	-7.2	2.26	b6 [3|4][1-6]	C47H65N9O10
1014.4871	1014.4931	-0.0060	-5.9	1.84	M -NH3 [7|1][1-7]	C51H67N9O13
1031.5154	1031.5197	-0.0042	-4.1	90.96	M [7|1][1-7]	C51H70N10O13

NMR

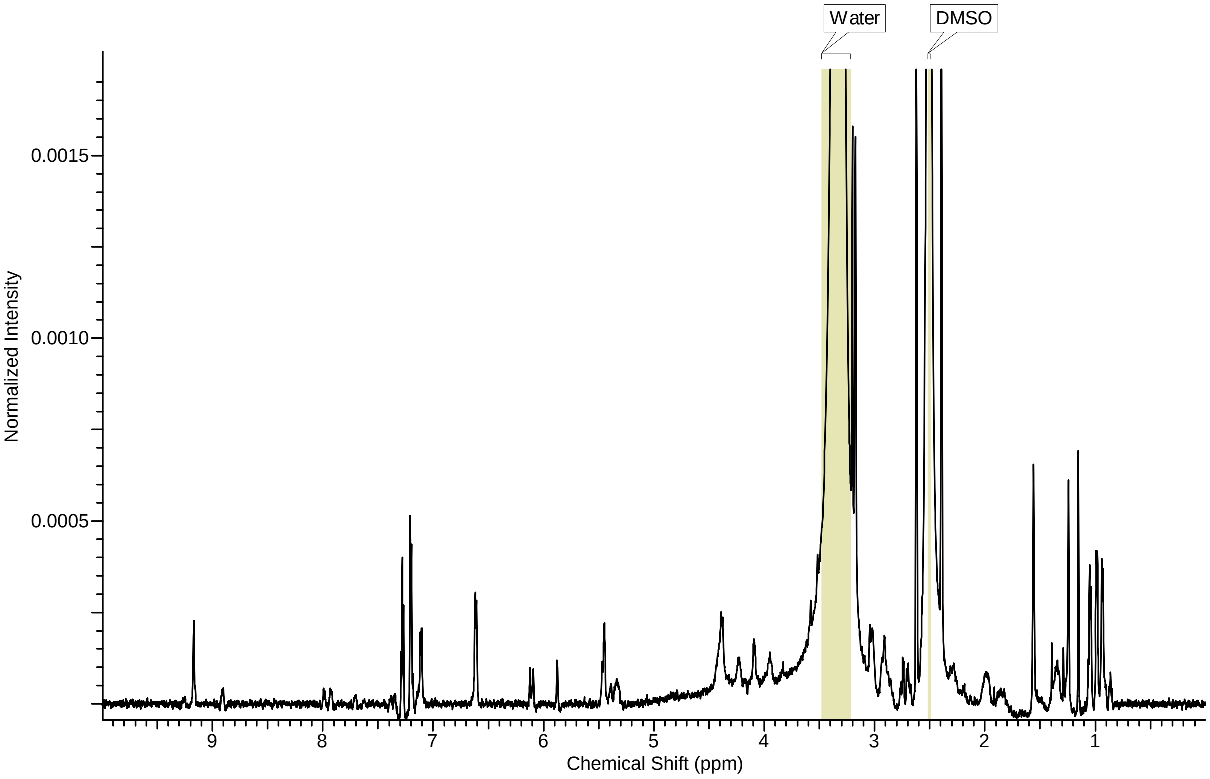

^{1}H-NMR spectrum agrees with the postulated structure.

[D-Asp³,(*E*)-Dhb⁷]MC-YR (15)

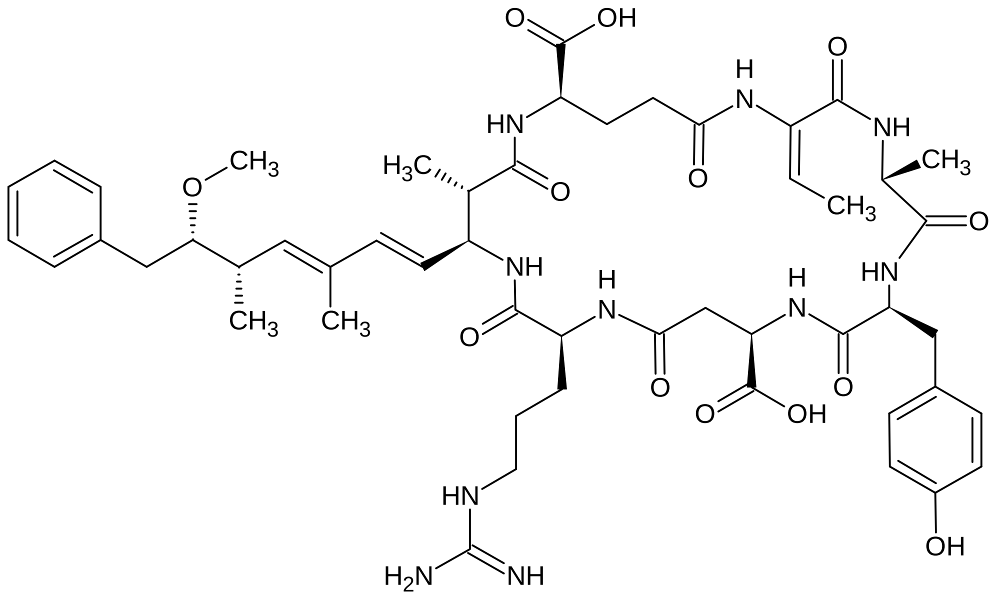

D-Ala | Tyr | D-Asp | Arg | Adda | D-Glu | Dhb

Formula (M)	Ion	Meas. m/z	Pred. m/z	Diff (mDa)	Diff (ppm)	Iso Score
C51 H70 N10 O13	[M+H]+	1031.5196	1031.5197	-0.1	-0.10	100.00

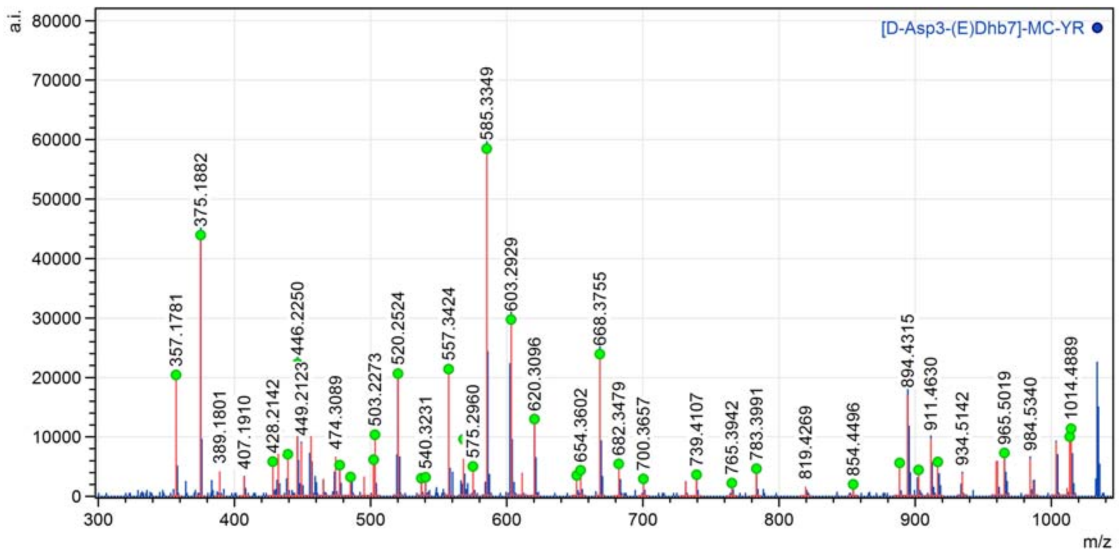

Length	Mo. Mass	Av. Mass	Coverage	Matched Int.
7 (Cyclic)	1030.5124	1031.1631	100.0 %	78.4 %

Meas. m/z	Calc. m/z	δ (Da)	δ (ppm)	Rel. Int. (%)	Annotation	Formula
357.1781	357.1809	-0.0027	-7.7	34.95	z3 -C9H10O -H2O [7|1][5-7]	C20H24N2O4
375.1882	375.1914	-0.0032	-8.7	75.31	z3 -C9H10O [7|1][5-7]	C20H26N2O5
428.2142	428.2180	-0.0038	-8.8	9.83	z4 -C9H10O -H2O [1|2][4-7]	C23H29N3O5
432.1876	432.1878	-0.0002	-0.4	18.63	c4 -H2O [6|7][1-4]	C20H25N5O6

439.2707	439.2789	-0.0082	-18.6	11.96	c3 -C9H11O -CN2H2 [3|4][1-3]	C21H36N5O5
446.2250	446.2286	-0.0036	-8.0	38.11	z4 -C9H10O [1|2][4-7]	C23H31N3O6
446.2250	446.2286	-0.0036	-8.0	38.11	b4 -C9H10O -NH3 [4|5][1-4]	C23H31N3O6
477.2425	477.2384	0.0041	8.5	8.70	b3 -CH3OH -NH3 [4|5][1-3]	C28H32N2O5
477.2425	477.2384	0.0041	8.5	8.70	z3 -CH3OH [7|1][5-7]	C28H32N2O5
485.2112	485.2031	0.0081	16.8	5.26	a5 -CH5N3 -NH3 [6|7][1-5]	C24H28N4O7
502.2363	502.2296	0.0067	13.3	10.30	a5 -CH5N3 [6|7][1-5]	C24H31N5O7
503.2273	503.2262	0.0011	2.1	17.43	z4 -C9H11O -CH5N3 [6|7][4-7]	C25H32N3O8
520.2524	520.2528	-0.0004	-0.8	35.09	z4 -C9H11O -CN2H2 [6|7][4-7]	C25H35N4O8
520.2524	520.2528	-0.0004	-0.8	35.09	b4 -C9H11O -CH5N3 [2|3][1-4]	C25H35N4O8
537.2870	537.2793	0.0077	14.3	4.90	b4 -C9H11O -CN2H2 [2|3][1-4]	C25H38N5O8
540.3231	540.3180	0.0050	9.3	5.12	a3 -NH3 [2|3][1-3]	C29H41N5O5
557.3424	557.3446	-0.0022	-3.9	36.36	a3 [2|3][1-3]	C29H44N6O5
557.3424	557.3334	0.0090	16.2	36.36	b3 -CN2H2 [3|4][1-3]	C30H44N4O6
568.3052	568.3130	-0.0077	-13.6	16.16	b3 -NH3 [2|3][1-3]	C30H41N5O6
568.3052	568.3130	-0.0077	-13.6	16.16	z3 [5|6][5-7]	C30H41N5O6
568.3052	568.3004	0.0048	8.5	16.16	a4 -C9H11O -NH3 [1|2][1-4]	C29H39N6O6
575.2960	575.2950	0.0010	1.8	8.25	a5 -C9H11O -CH5N3 [2|3][1-5]	C28H40N5O8
585.3349	585.3395	-0.0046	-7.8	100.00	b3 [2|3][1-3]	C30H44N6O6
585.3349	585.3269	0.0080	13.7	100.00	a4 -C9H11O [1|2][1-4]	C29H42N7O6
603.2929	603.2899	0.0030	5.0	50.74	b5 -C9H11O -CH5N3 [2|3][1-5]	C29H40N5O9
603.2929	603.2899	0.0030	5.0	50.74	z5 -C9H11O -CN2H2 [7|1][3-7]	C29H40N5O9
620.3096	620.3164	-0.0068	-11.0	21.95	b5 -C9H11O -CN2H2 [2|3][1-5]	C29H43N6O9
651.3441	651.3501	-0.0060	-9.2	5.76	a4 -H2O -NH3 [2|3][1-4]	C34H46N6O7
654.3602	654.3610	-0.0008	-1.2	7.25	a4 -CH3OH [2|3][1-4]	C33H47N7O7
668.3755	668.3766	-0.0012	-1.7	40.85	a4 -H2O [2|3][1-4]	C34H49N7O7
682.3479	682.3559	-0.0080	-11.7	9.16	b4 -CH3OH [2|3][1-4]	C34H47N7O8
700.3657	700.3705	-0.0048	-6.9	4.85	a5 -CH3OH [4|5][1-5]	C39H49N5O7
700.3657	700.3705	-0.0048	-6.9	4.85	a5 -CH3OH -CH5N3 [7|1][1-5]	C39H49N5O7
739.4107	739.4012	0.0095	12.9	6.09	a6 -C9H11O [6|7][1-6]	C36H52N9O8
765.3942	765.3930	0.0012	1.5	3.69	b6 -C9H10O -NH3 [3|4][1-6]	C38H52N8O9
765.3942	765.3930	0.0012	1.5	3.69	z6 -C9H10O [2|3][2-7]	C38H52N8O9
765.3942	765.3930	0.0012	1.5	3.69	b5 -CH3OH [2|3][1-5]	C38H52N8O9
783.3991	783.4076	-0.0085	-10.9	7.80	a6 -CH3OH -CH5N3 [6|7][1-6]	C43H54N6O8
854.4496	854.4447	0.0049	5.7	3.21	c6 -CHN3H6 -H2O [3|4][1-6]	C46H59N7O9
854.4496	854.4447	0.0049	5.7	3.21	a6 -NH3 -NH3 [3|4][1-6]	C46H59N7O9
888.5000	888.4978	0.0022	2.5	9.37	a6 [3|4][1-6]	C46H65N9O9
902.4816	902.4771	0.0045	5.0	7.32	a6 -H2O [7|1][1-6]	C46H63N9O10
902.4816	902.4771	0.0045	5.0	7.32	b6 [6|7][1-6]	C46H63N9O10
916.4943	916.4927	0.0015	1.7	9.57	b6 [3|4][1-6]	C47H65N9O10
965.5019	965.5091	-0.0071	-7.4	12.12	c6 [7|1][1-6]	C47H68N10O12
1013.5012	1013.5091	-0.0079	-7.8	16.79	M -H2O [7|1][1-7]	C51H68N10O12
1014.4889	1014.4931	-0.0042	-4.1	19.16	M -NH3 [7|1][1-7]	C51H67N9O13

NMR

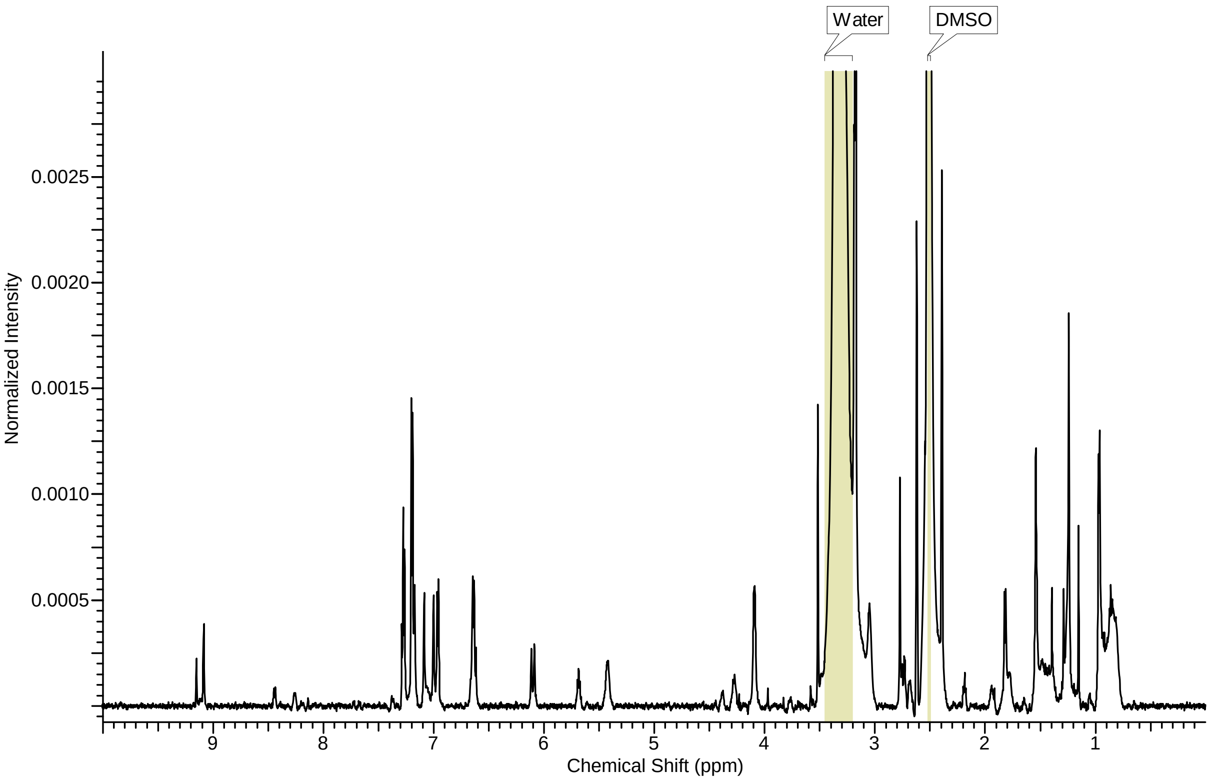

1H-NMR spectrum agrees with the postulated structure. This compound has not been described in the literature before.

MC-RY (16)

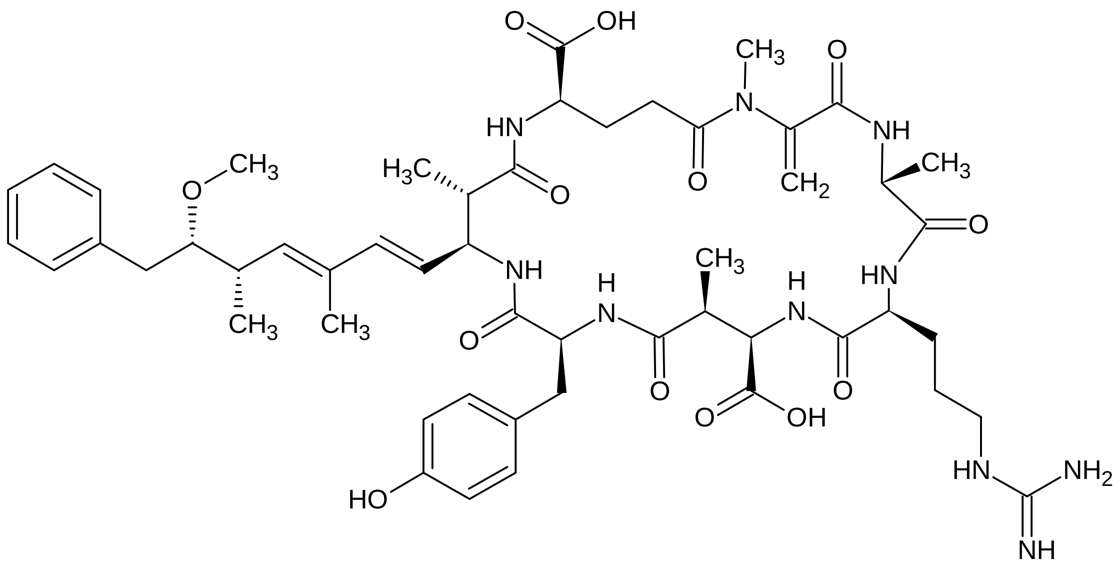

D-Ala | Arg | D-MeAsp | Tyr | Adda | D-Glu | Mdha

Formula (M)	Ion	Meas. m/z	Pred. m/z	Diff (mDa)	Diff (ppm)	Iso Score
C52 H72 N10 O13	[M+H]+	1045.5368	1045.5353	1.5	1.43	100.00

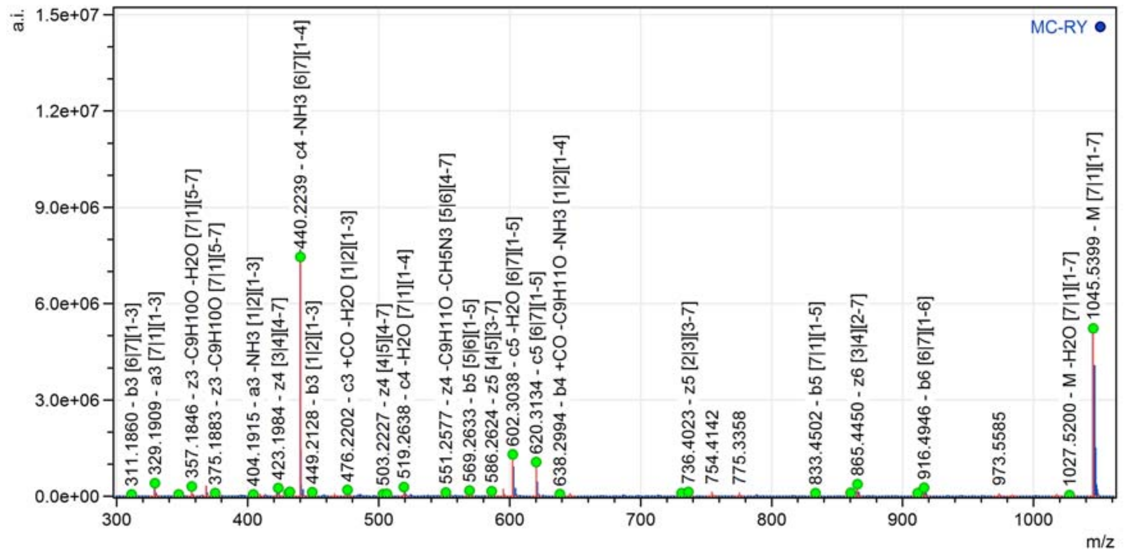

Length	Mo. Mass	Av. Mass	Coverage	Matched Int.
7 (Cyclic)	1044.5280	1045.1897	100.0 %	93.5 %

Meas. m/z	Calc. m/z	δ (Da)	δ (ppm)	Rel. Int. (%)	Annotation	Formula
311.1860	311.1826	0.0034	11.0	0.75	b3 [6|7][1-3]	C13H22N6O3
311.1860	311.1826	0.0034	11.0	0.75	a3 -H2O [7|1][1-3]	C13H22N6O3
329.1909	329.1932	-0.0023	-6.9	5.44	a3 [7|1][1-3]	C13H24N6O4
347.1905	347.1965	-0.0060	-17.4	0.71	a3 -C9H10O -NH3 [4|5][1-3]	C19H26N2O4
357.1846	357.1881	-0.0035	-9.9	4.10	b3 [7|1][1-3]	C14H24N6O5

357.1846	357.1809	0.0037	10.3	4.10	z3 -C9H10O -H2O [7|1][5-7]	C20H24N2O4
375.1883	375.1914	-0.0031	-8.3	1.28	z3 -C9H10O [7|1][5-7]	C20H26N2O5
404.1915	404.1928	-0.0013	-3.2	0.69	a3 -NH3 [1|2][1-3]	C19H25N5O5
423.1984	423.1987	-0.0003	-0.7	3.38	b4 -NH3 [5|6][1-4]	C18H26N6O6
423.1984	423.1987	-0.0003	-0.7	3.38	z4 [3|4][4-7]	C18H26N6O6
423.1984	423.1987	-0.0003	-0.7	3.38	b4 -NH3 [6|7][1-4]	C18H26N6O6
423.1984	423.1987	-0.0003	-0.7	3.38	z4 [2|3][4-7]	C18H26N6O6
431.2048	431.2037	0.0011	2.4	1.46	b3 -H2O [1|2][1-3]	C20H26N6O5
432.1889	432.1878	0.0011	2.5	1.81	b3 -NH3 [1|2][1-3]	C20H25N5O6
432.1889	432.1878	0.0011	2.5	1.81	z3 [4|5][5-7]	C20H25N5O6
440.2239	440.2252	-0.0013	-3.1	100.00	b4 [5|6][1-4]	C18H29N7O6
440.2239	440.2252	-0.0013	-3.1	100.00	b4 [6|7][1-4]	C18H29N7O6
440.2239	440.2252	-0.0013	-3.1	100.00	c4 -NH3 [6|7][1-4]	C18H29N7O6
449.2128	449.2143	-0.0015	-3.4	1.63	b3 [1|2][1-3]	C20H28N6O6
476.2202	476.2252	-0.0050	-10.6	2.61	c3 +CO -H2O [1|2][1-3]	C21H29N7O6
503.2227	503.2249	-0.0021	-4.2	0.88	b4 -NH3 [7|1][1-4]	C23H30N6O7
503.2227	503.2249	-0.0021	-4.2	0.88	z4 [4|5][4-7]	C23H30N6O7
506.2309	506.2358	-0.0049	-9.7	0.96	a5 -H2O -NH3 [5|6][1-5]	C22H31N7O7
506.2309	506.2245	0.0063	12.5	0.96	c4 +CO -CH5N3 [7|1][1-4]	C23H31N5O8
506.2309	506.2245	0.0063	12.5	0.96	b4 +CO -CN2H2 [7|1][1-4]	C23H31N5O8
519.2638	519.2674	-0.0036	-6.9	3.83	c4 -H2O [7|1][1-4]	C23H34N8O6
551.2577	551.2626	-0.0049	-8.9	1.61	z4 -C9H11O -CH5N3 [5|6][4-7]	C30H36N3O7
551.2577	551.2572	0.0004	0.8	1.61	b5 -H2O [5|6][1-5]	C23H34N8O8
569.2633	569.2678	-0.0045	-7.9	2.34	b5 [5|6][1-5]	C23H36N8O9
586.2624	586.2620	0.0004	0.7	2.02	z5 [4|5][3-7]	C27H35N7O8
586.2624	586.2620	0.0004	0.7	2.02	b5 -NH3 [6|7][1-5]	C27H35N7O8
602.3038	602.3059	-0.0020	-3.4	17.48	c5 +CO -C9H11O -CHN3H6 [4|5][1-5]	C29H41N6O8
602.3038	602.3045	-0.0007	-1.2	17.48	c5 -H2O [6|7][1-5]	C27H39N9O7
620.3134	620.3151	-0.0017	-2.7	14.23	c5 [6|7][1-5]	C27H41N9O8
638.2994	638.3059	-0.0065	-10.1	0.85	b4 +CO -C9H11O -NH3 [1|2][1-4]	C32H41N6O8
638.2994	638.2946	0.0048	7.5	0.85	a5 -C9H11O -NH3 [2|3][1-5]	C33H41N4O9
731.3616	731.3651	-0.0035	-4.8	1.31	b4 +CO -CH5N3 [1|2][1-4]	C40H50N4O9
736.4023	736.4028	-0.0005	-0.7	1.75	b5 -NH3 [4|5][1-5]	C38H53N7O8
736.4023	736.4028	-0.0005	-0.7	1.75	z5 [2|3][3-7]	C38H53N7O8
833.4502	833.4556	-0.0054	-6.5	1.22	b5 [7|1][1-5]	C43H60N8O9
860.4649	860.4665	-0.0016	-1.8	1.38	c5 +CO -H2O [7|1][1-5]	C44H61N9O9
860.4649	860.4553	0.0097	11.2	1.38	a6 -CH3OH -CN2H2 [7|1][1-6]	C45H61N7O10
865.4450	865.4454	-0.0005	-0.5	4.99	z6 [3|4][2-7]	C43H60N8O11
865.4450	865.4454	-0.0005	-0.5	4.99	b6 -NH3 [4|5][1-6]	C43H60N8O11
911.4654	911.4662	-0.0008	-0.9	1.29	a6 -H2O -NH3 [1|2][1-6]	C48H62N8O10
911.4654	911.4621	0.0032	3.5	1.29	M -C9H10O [7|1][1-7]	C43H62N10O12
916.4946	916.4927	0.0019	2.1	3.53	b6 [3|4][1-6]	C47H65N9O10
916.4946	916.4927	0.0019	2.1	3.53	a6 -H2O [7|1][1-6]	C47H65N9O10
916.4946	916.4927	0.0019	2.1	3.53	b6 [6|7][1-6]	C47H65N9O10
1027.5200	1027.5247	-0.0047	-4.6	0.46	M -H2O [7|1][1-7]	C52H70N10O12
1045.5399	1045.5353	0.0045	4.3	70.10	M [7|1][1-7]	C52H72N10O13

NMR

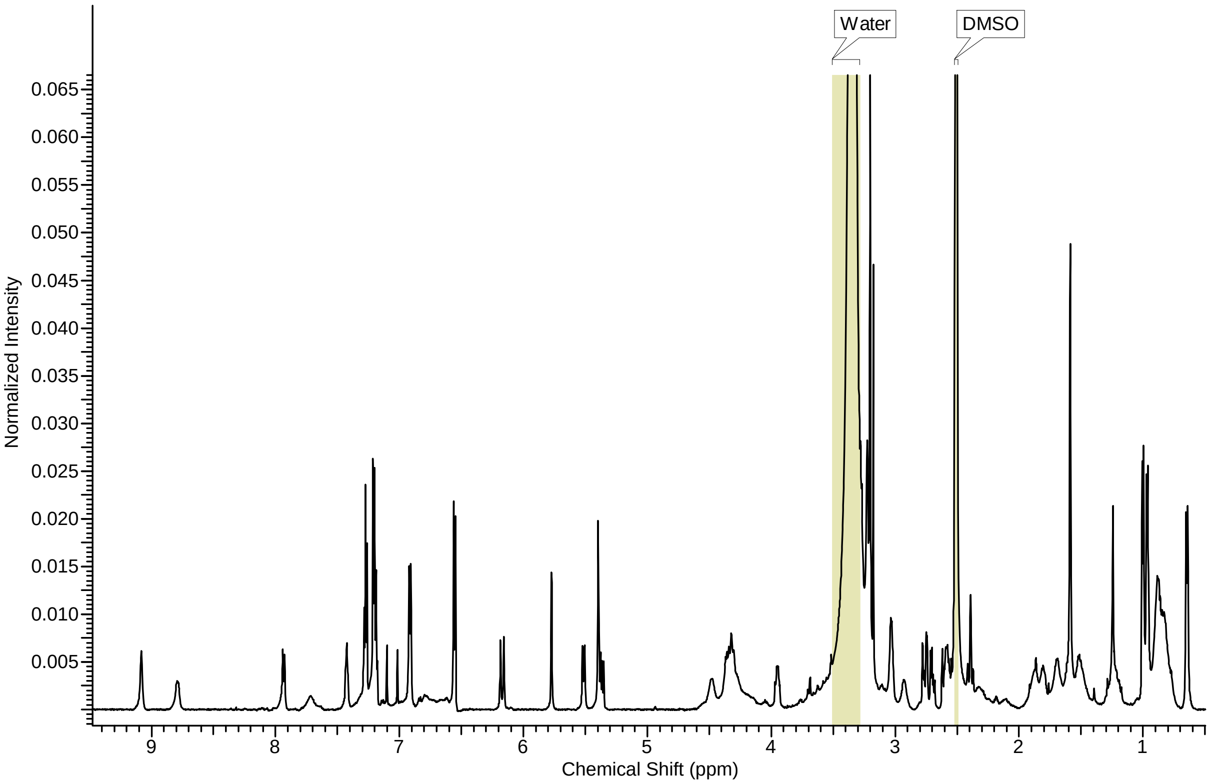

^{1}H-NMR spectrum agrees with the postulated structure and is comparable to the spectrum described for Microcystin-RY by Miles et al., Toxicon, 2013, 21-31

MC-YY (17)

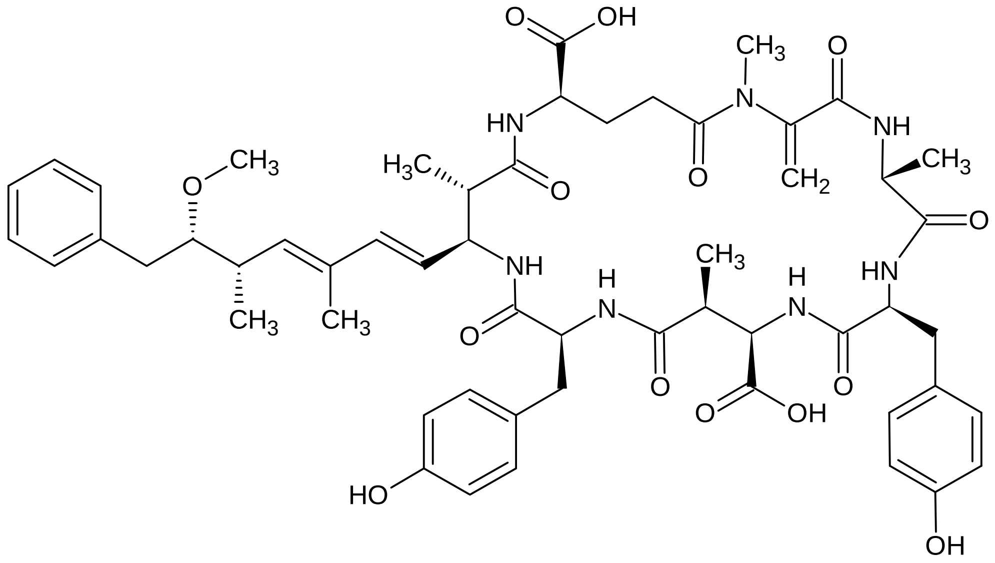

D-Ala | Tyr | D-MeAsp | Tyr | Adda | D-Glu | Mdha

Formula (M)	Ion	Meas. m/z	Pred. m/z	Diff (mDa)	Diff (ppm)	Iso Score
C55 H69 N7 O14	[M+H]+	1052.5000	1052.4975	2.5	2.38	97.48

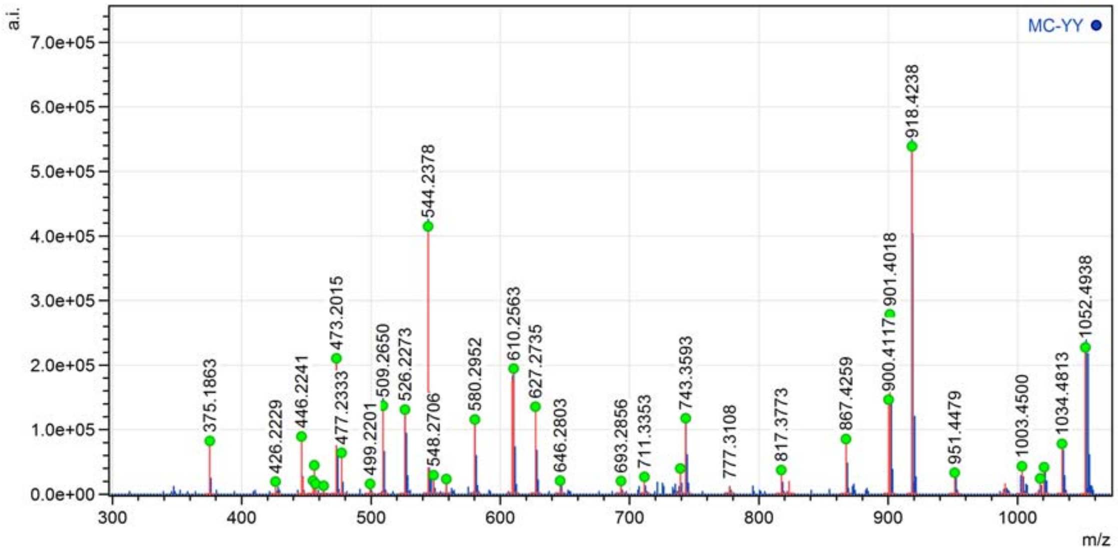

Length	Mo. Mass	Av. Mass	Coverage	Matched Int.
7 (Cyclic)	1051.4902	1052.1773	100.0 %	93.6 %

Meas. m/z	Calc. m/z	δ (Da)	δ (ppm)	Rel. Int. (%)	Annotation	Formula
375.1863	375.1914	-0.0051	-13.7	15.28	z3 -C9H10O [7|1][5-7]	C20H26N2O5
426.2229	426.2275	-0.0046	-10.7	3.44	b2 -NH3 [4|5][1-2]	C25H31NO5
426.2229	426.2149	0.0080	18.8	3.44	a3 -C9H11O -NH3 [2|3][1-3]	C24H29N2O5
446.2241	446.2286	-0.0045	-10.1	16.48	z4 -C9H10O [1|2][4-7]	C23H31N3O6
455.1862	455.1925	-0.0063	-13.9	3.70	c3 -H2O [1|2][1-3]	C23H26N4O6
456.1739	456.1765	-0.0026	-5.7	8.08	b3 [1|2][1-3]	C23H25N3O7
457.1814	457.1718	0.0096	21.0	2.78	b4 +CO -H2O [5|6][1-4]	C22H24N4O7
457.1814	457.1718	0.0096	21.0	2.78	b4 +CO -H2O [6|7][1-4]	C22H24N4O7
463.2585	463.2591	-0.0007	-1.5	2.21	a3 -H2O -NH3 [4|5][1-3]	C28H34N2O4
463.2585	463.2551	0.0033	7.2	2.21	b4 -C9H10O [4|5][1-4]	C23H34N4O6
473.2015	473.2031	-0.0016	-3.3	38.95	c3 [1|2][1-3]	C23H28N4O7
477.2333	477.2384	-0.0051	-10.6	11.65	b3 -CH3OH -NH3 [4|5][1-3]	C28H32N2O5
499.2201	499.2187	0.0013	2.7	2.71	a4 [7|1][1-4]	C25H30N4O7
509.2650	509.2646	0.0003	0.7	25.21	b3 -NH3 [4|5][1-3]	C29H36N2O6
526.2273	526.2296	-0.0023	-4.4	24.07	c4 -H2O [7|1][1-4]	C26H31N5O7
544.2378	544.2402	-0.0023	-4.3	76.87	c4 [7|1][1-4]	C26H33N5O8
548.2706	548.2755	-0.0049	-8.9	5.20	b4 -CH3OH -NH3 [4|5][1-4]	C31H37N3O6
548.2706	548.2755	-0.0049	-8.9	5.20	z4 -CH3OH [1|2][4-7]	C31H37N3O6
558.2160	558.2195	-0.0035	-6.2	4.05	b5 -H2O [5|6][1-5]	C26H31N5O9
580.2952	580.3017	-0.0065	-11.2	21.18	z4 [1|2][4-7]	C32H41N3O7
580.2952	580.3017	-0.0065	-11.2	21.18	b4 -NH3 [4|5][1-4]	C32H41N3O7
610.2563	610.2508	0.0055	9.1	35.85	b5 [6|7][1-5]	C30H35N5O9
627.2735	627.2773	-0.0038	-6.1	24.89	c5 [6|7][1-5]	C30H38N6O9
646.2803	646.2759	0.0044	6.8	3.57	b4 +CO -C9H10O -NH3 [1|2][1-4]	C35H39N3O9
693.2856	693.2879	-0.0023	-3.3	3.47	a6 -H2O [5|6][1-6]	C34H40N6O10
711.3353	711.3388	-0.0035	-5.0	4.69	b5 -CH3OH -NH3 [3|4][1-5]	C40H46N4O8
711.3353	711.3388	-0.0035	-5.0	4.69	b5 -CH3OH -NH3 [4|5][1-5]	C40H46N4O8
711.3353	711.3388	-0.0035	-5.0	4.69	z5 -CH3OH [2|3][3-7]	C40H46N4O8
711.3353	711.3388	-0.0035	-5.0	4.69	z5 -CH3OH [1|2][3-7]	C40H46N4O8
739.2904	739.2933	-0.0030	-4.0	7.12	b6 [5|6][1-6]	C35H42N6O12
743.3593	743.3651	-0.0058	-7.8	21.60	z5 [1|2][3-7]	C41H50N4O9
743.3593	743.3651	-0.0058	-7.8	21.60	b5 -NH3 [4|5][1-5]	C41H50N4O9
743.3593	743.3651	-0.0058	-7.8	21.60	b5 -NH3 [3|4][1-5]	C41H50N4O9
743.3593	743.3651	-0.0058	-7.8	21.60	z5 [2|3][3-7]	C41H50N4O9
743.3593	743.3525	0.0068	9.1	21.60	a6 -C9H11O -NH3 [6|7][1-6]	C40H48N5O9
743.3593	743.3525	0.0068	9.1	21.60	a6 -C9H11O -NH3 [3|4][1-6]	C40H48N5O9
817.3773	817.3767	0.0006	0.8	6.66	b6 +CO -C9H10O [6|7][1-6]	C42H52N6O11
817.3773	817.3767	0.0006	0.8	6.66	b6 +CO -C9H10O [3|4][1-6]	C42H52N6O11
817.3773	817.3767	0.0006	0.8	6.66	b6 -C9H10O -H2O [7|1][1-6]	C42H52N6O11
867.4259	867.4287	-0.0029	-3.3	15.63	c5 +CO -H2O [7|1][1-5]	C47H58N6O10
900.4117	900.4138	-0.0021	-2.3	26.94	M -C9H10O -H2O [7|1][1-7]	C46H57N7O12
901.4018	901.3978	0.0040	4.4	51.50	M -C9H10O -NH3 [7|1][1-7]	C46H56N6O13
918.4238	918.4244	-0.0005	-0.6	100.00	M -C9H10O [7|1][1-7]	C46H59N7O13

951.4479	951.4498	-0.0019	-2.0	5.94	b6 -H2O [7|1][1-6]	C51H62N6O12
951.4479	951.4498	-0.0019	-2.0	5.94	b6 +CO [6|7][1-6]	C51H62N6O12
951.4479	951.4498	-0.0019	-2.0	5.94	b6 +CO [3|4][1-6]	C51H62N6O12
1003.4500	1003.4448	0.0053	5.3	7.81	M -CH3OH -NH3 [7|1][1-7]	C54H62N6O13
1017.4564	1017.4604	-0.0040	-3.9	4.37	M -H2O -NH3 [7|1][1-7]	C55H64N6O13
1020.4725	1020.4713	0.0012	1.2	7.51	M -CH3OH [7|1][1-7]	C54H65N7O13
1034.4813	1034.4870	-0.0057	-5.5	14.29	M -H2O [7|1][1-7]	C55H67N7O13
1052.4938	1052.4975	-0.0038	-3.6	42.14	M [7|1][1-7]	C55H69N7O14

NMR

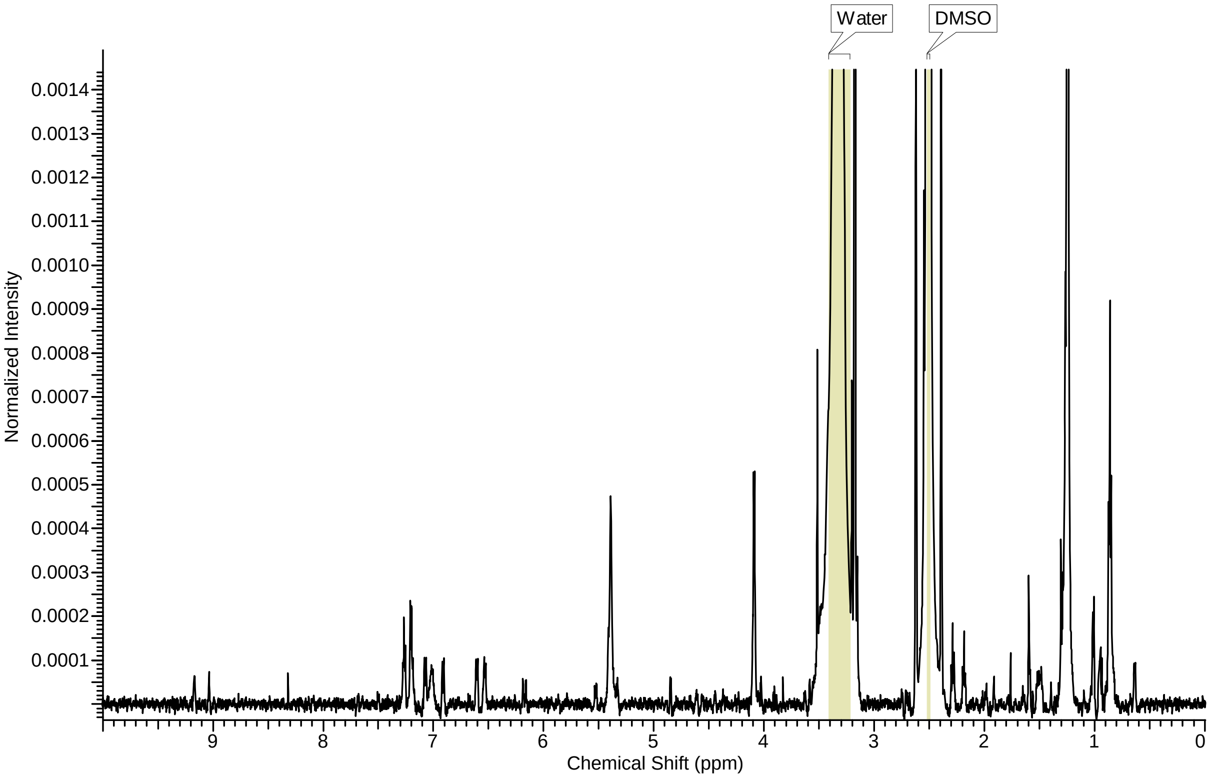

^{1}H-NMR spectrum agrees with the postulated structure.

[D-Asp[3],(*E*)-Dhb[7]]MC-HtyY (18)

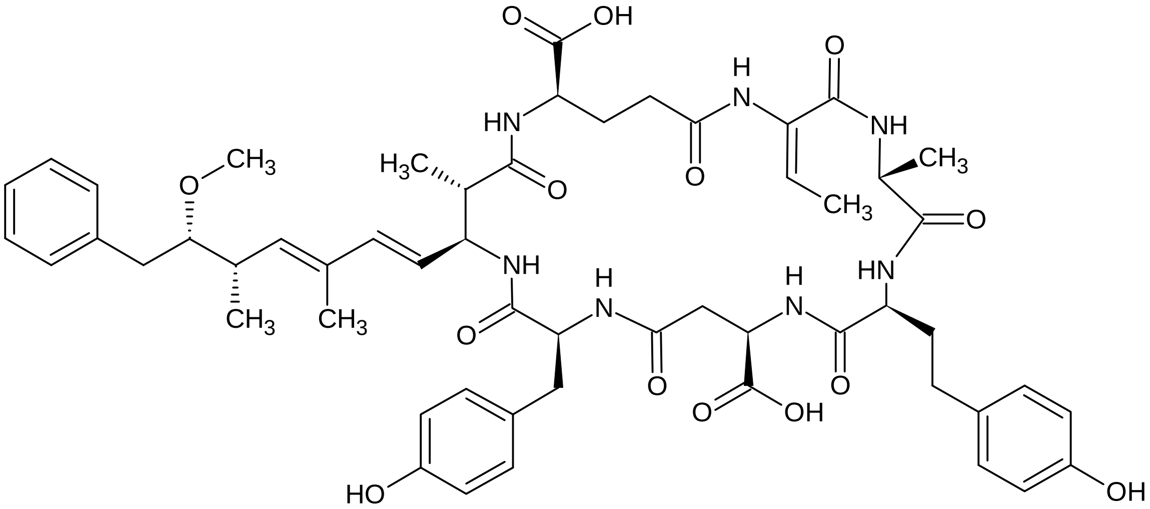

D-Ala | L-Hty | D-Asp | L-Tyr | Adda | D-Glu | Dhb

Formula (M)	Ion	Meas. m/z	Pred. m/z	Diff (mDa)	Diff (ppm)	Iso Score
C55 H69 N7 O14	[M+H]+	1052.4981	1052.4975	0.6	0.57	99.02

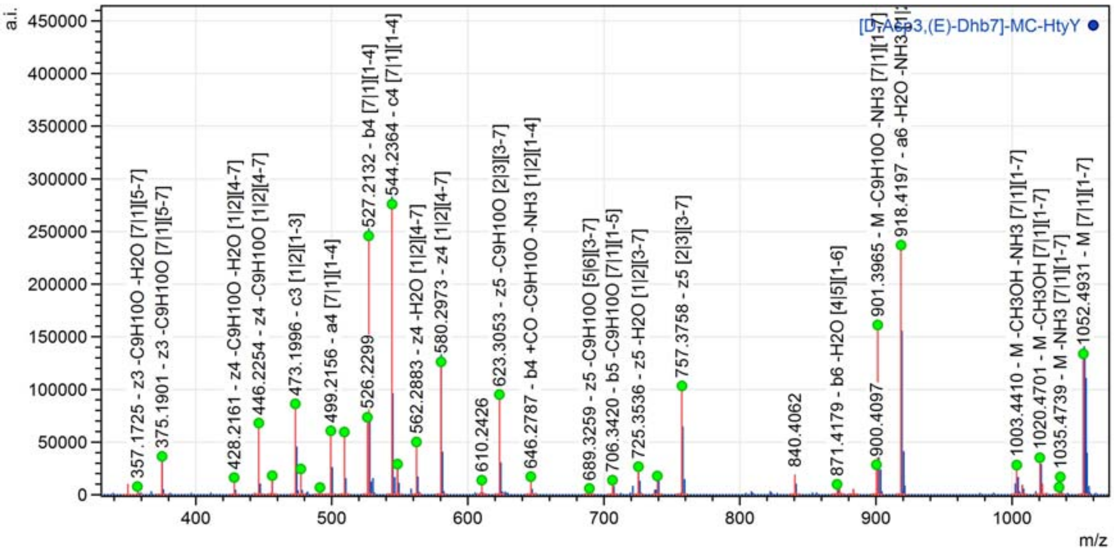

Length	Mo. Mass	Av. Mass	Coverage	Matched Int.
7 (Cyclic)	1051.4902	1052.1773	100.0 %	97.2 %

Meas. m/z	Calc. m/z	δ (Da)	δ (ppm)	Rel. Int. (%)	Annotation	Formula
357.1725	357.1809	-0.0084	-23.5	2.78	z3 -C9H10O -H2O [7|1][5-7]	C20H24N2O4
375.1901	375.1914	-0.0014	-3.7	13.17	b3 -C9H10O -NH3 [4|5][1-3]	C20H26N2O5
375.1901	375.1914	-0.0014	-3.7	13.17	z3 -C9H10O [7|1][5-7]	C20H26N2O5
428.2161	428.2220	-0.0059	-13.8	5.72	b2 -CH3OH -NH3 [3|4][1-2]	C28H29NO3
428.2161	428.2220	-0.0059	-13.8	5.72	z2 -CH3OH [5|6][6-7]	C28H29NO3
428.2161	428.2180	-0.0019	-4.4	5.72	z4 -C9H10O -H2O [1|2][4-7]	C23H29N3O5
446.2254	446.2286	-0.0032	-7.1	24.55	z4 -C9H10O [1|2][4-7]	C23H31N3O6
446.2254	446.2286	-0.0032	-7.1	24.55	b4 -C9H10O -NH3 [4|5][1-4]	C23H31N3O6
456.1733	456.1765	-0.0032	-7.0	6.43	b3 [1|2][1-3]	C23H25N3O7
473.1996	473.2031	-0.0035	-7.4	31.09	c3 [1|2][1-3]	C23H28N4O7
477.2341	477.2384	-0.0043	-9.0	8.72	z3 -CH3OH [7|1][5-7]	C28H32N2O5
477.2341	477.2384	-0.0043	-9.0	8.72	b3 -CH3OH -NH3 [4|5][1-3]	C28H32N2O5
491.2585	491.2540	0.0045	9.2	2.24	b3 -H2O -NH3 [4|5][1-3]	C29H34N2O5
491.2585	491.2540	0.0045	9.2	2.24	z3 -H2O [7|1][5-7]	C29H34N2O5
499.2156	499.2187	-0.0031	-6.2	21.86	a4 [7|1][1-4]	C25H30N4O7
509.2622	509.2646	-0.0025	-4.8	21.43	z3 [7|1][5-7]	C29H36N2O6
509.2622	509.2646	-0.0025	-4.8	21.43	b3 -NH3 [4|5][1-3]	C29H36N2O6
526.2299	526.2296	0.0003	0.5	26.50	c4 -H2O [7|1][1-4]	C26H31N5O7
526.2299	526.2224	0.0075	14.2	26.50	z3 -CH3OH -NH3 [5|6][5-7]	C32H31NO6
527.2132	527.2136	-0.0005	-0.9	89.09	b4 [7|1][1-4]	C26H30N4O8
544.2364	544.2402	-0.0037	-6.9	100.00	c4 [7|1][1-4]	C26H33N5O8
548.2738	548.2755	-0.0017	-3.1	10.37	z4 -CH3OH [1|2][4-7]	C31H37N3O6
548.2738	548.2755	-0.0017	-3.1	10.37	b4 -CH3OH -NH3 [4|5][1-4]	C31H37N3O6
562.2883	562.2912	-0.0029	-5.1	17.98	b4 -H2O -NH3 [4|5][1-4]	C32H39N3O6
562.2883	562.2912	-0.0029	-5.1	17.98	z4 -H2O [1|2][4-7]	C32H39N3O6
580.2973	580.3017	-0.0044	-7.6	45.60	z4 [1|2][4-7]	C32H41N3O7
580.2973	580.3017	-0.0044	-7.6	45.60	b4 -NH3 [4|5][1-4]	C32H41N3O7
610.2426	610.2508	-0.0081	-13.3	4.83	b5 [6|7][1-5]	C30H35N5O9
623.3053	623.3075	-0.0022	-3.5	34.35	z5 -C9H10O [2|3][3-7]	C33H42N4O8
623.3053	623.3075	-0.0022	-3.5	34.35	b5 -C9H10O -NH3 [4|5][1-5]	C33H42N4O8
646.2787	646.2759	0.0028	4.4	5.98	b4 +CO -C9H10O -NH3 [1|2][1-4]	C35H39N3O9
689.3259	689.3181	0.0078	11.3	1.91	b4 -CH3OH [2|3][1-4]	C37H44N4O9
689.3259	689.3181	0.0078	11.3	1.91	b5 -C9H10O -NH3 [7|1][1-5]	C37H44N4O9
689.3259	689.3181	0.0078	11.3	1.91	z5 -C9H10O [5|6][3-7]	C37H44N4O9
706.3420	706.3487	-0.0067	-9.4	4.84	a4 -H2O -NH3 [1|2][1-4]	C42H47N3O7
706.3420	706.3447	-0.0026	-3.7	4.84	b5 -C9H10O [7|1][1-5]	C37H47N5O9
725.3536	725.3545	-0.0009	-1.2	9.51	b5 -CH3OH -NH3 [4|5][1-5]	C41H48N4O8
725.3536	725.3545	-0.0009	-1.2	9.51	z5 -CH3OH [2|3][3-7]	C41H48N4O8
725.3536	725.3545	-0.0009	-1.2	9.51	b5 -H2O -NH3 [3|4][1-5]	C41H48N4O8
725.3536	725.3545	-0.0009	-1.2	9.51	z5 -H2O [1|2][3-7]	C41H48N4O8
739.3591	739.3535	0.0055	7.4	6.24	c6 -C9H11O -H2O [2|3][1-6]	C36H48N7O10
757.3758	757.3807	-0.0049	-6.5	37.38	z5 [2|3][3-7]	C42H52N4O9
757.3758	757.3807	-0.0049	-6.5	37.38	b5 -NH3 [4|5][1-5]	C42H52N4O9
757.3758	757.3681	0.0077	10.1	37.38	a6 -C9H11O -NH3 [3|4][1-6]	C41H50N5O9

871.4179	871.4236	-0.0057	-6.5	3.40	b6 -H2O [4|5][1-6]	C46H58N6O11
900.4097	900.4138	-0.0041	-4.5	10.11	M -C9H10O -H2O [7|1][1-7]	C46H57N7O12
901.3965	901.3978	-0.0013	-1.5	58.32	M -C9H10O -NH3 [7|1][1-7]	C46H56N6O13
918.4197	918.4284	-0.0087	-9.4	85.84	a6 -H2O -NH3 [1|2][1-6]	C51H59N5O11
918.4197	918.4244	-0.0046	-5.1	85.84	M -C9H10O [7|1][1-7]	C46H59N7O13
1003.4410	1003.4448	-0.0038	-3.8	9.99	M -CH3OH -NH3 [7|1][1-7]	C54H62N6O13
1020.4701	1020.4713	-0.0012	-1.2	12.56	M -CH3OH [7|1][1-7]	C54H65N7O13
1034.4905	1034.4870	0.0036	3.4	2.38	M -H2O [7|1][1-7]	C55H67N7O13
1035.4739	1035.4710	0.0029	2.8	5.93	M -NH3 [7|1][1-7]	C55H66N6O14
1052.4931	1052.4975	-0.0044	-4.2	48.39	M [7|1][1-7]	C55H69N7O14

NMR

See Christiansen et al., J. Nat. Prod. 2008, 1881-1886

[D-Asp^3,(*E*)-Dhb^7]MC-HtyHty (19)

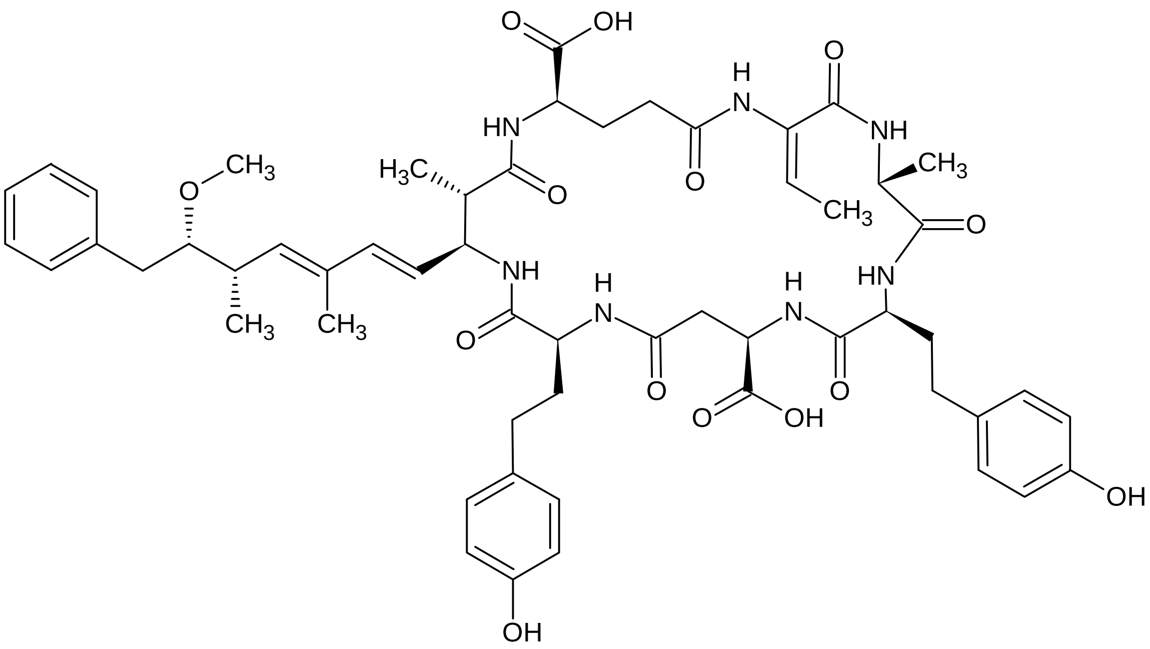

D-Ala | Hty | D-Asp | Hty | Adda | D-Glu | Dhb

Formula (M)	Ion	Meas. m/z	Pred. m/z	Diff (mDa)	Diff (ppm)	Iso Score
C56 H71 N7 O14	[M+H]+	1066.5109	1066.5132	-2.3	-2.16	75.81

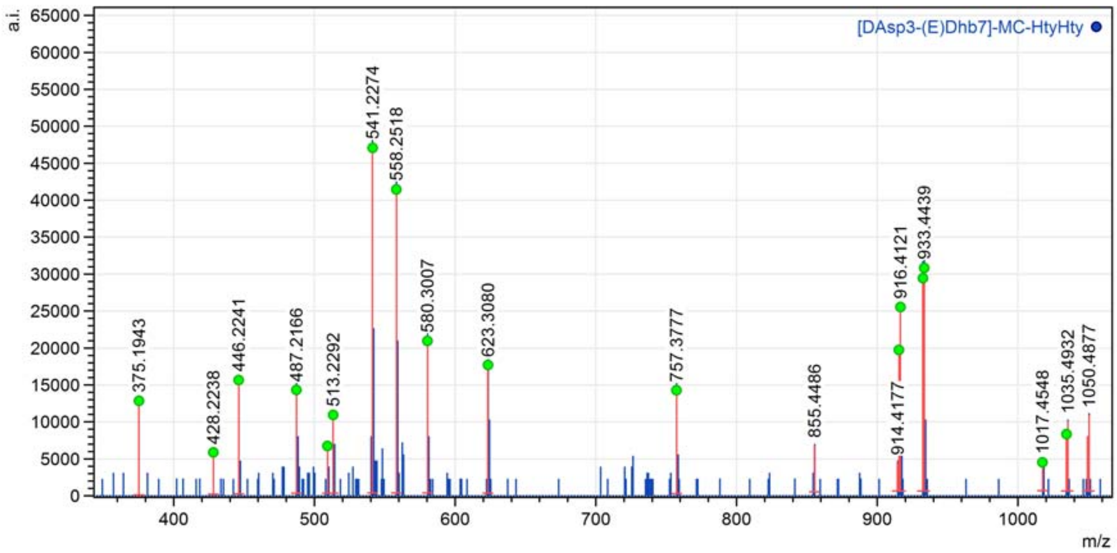

Length	Mo. Mass	Av. Mass	Coverage	Matched Int.
7 (Cyclic)	1065.5059	1066.2039	100.0 %	89.7 %

Meas. m/z	Calc. m/z	δ (Da)	δ (ppm)	Rel. Int. (%)	Annotation	Formula
375.1943	375.1914	0.0028	7.5	27.27	z3 -C9H10O [7|1][5-7]	C20H26N2O5
428.2238	428.2180	0.0058	13.4	12.05	z4 -C9H10O -H2O [1|2][4-7]	C23H29N3O5

446.2241	446.2286	-0.0044	-10.0	33.01	z4 -C9H10O [1|2][4-7]	C23H31N3O6
487.2166	487.2187	-0.0022	-4.4	29.92	c3 [1|2][1-3]	C24H30N4O7
509.2614	509.2646	-0.0032	-6.3	13.65	z3 [7|1][5-7]	C29H36N2O6
509.2614	509.2646	-0.0032	-6.3	13.65	b3 -NH3 [4|5][1-3]	C29H36N2O6
513.2292	513.2344	-0.0052	-10.1	22.63	b4 -CO [7|1][1-4]	C26H32N4O7
513.2292	513.2344	-0.0052	-10.1	22.63	a4 [7|1][1-4]	C26H32N4O7
541.2274	541.2293	-0.0019	-3.5	100.00	b4 [7|1][1-4]	C27H32N4O8
558.2518	558.2558	-0.0040	-7.2	87.94	c4 [7|1][1-4]	C27H35N5O8
580.3007	580.3017	-0.0010	-1.8	44.00	z4 [1|2][4-7]	C32H41N3O7
580.3007	580.3017	-0.0010	-1.8	44.00	b4 -NH3 [4|5][1-4]	C32H41N3O7
623.3080	623.3075	0.0005	0.8	37.15	b5 -C9H10O -NH3 [3|4][1-5]	C33H42N4O8
623.3080	623.3075	0.0005	0.8	37.15	z5 -C9H10O [2|3][3-7]	C33H42N4O8
623.3080	623.3075	0.0005	0.8	37.15	z5 -C9H10O [1|2][3-7]	C33H42N4O8
623.3080	623.3075	0.0005	0.8	37.15	b5 -C9H10O -NH3 [4|5][1-5]	C33H42N4O8
757.3777	757.3807	-0.0030	-4.0	29.93	b5 -NH3 [3|4][1-5]	C42H52N4O9
757.3777	757.3807	-0.0030	-4.0	29.93	b5 -NH3 [4|5][1-5]	C42H52N4O9
757.3777	757.3807	-0.0030	-4.0	29.93	z5 [2|3][3-7]	C42H52N4O9
757.3777	757.3807	-0.0030	-4.0	29.93	z5 [1|2][3-7]	C42H52N4O9
757.3777	757.3681	0.0096	12.6	29.93	z6 -C9H11O -CO [5|6][2-7]	C41H50N5O9
757.3777	757.3681	0.0096	12.6	29.93	a6 -C9H11O -NH3 [6|7][1-6]	C41H50N5O9
915.4151	915.4135	0.0016	1.7	40.87	M -C9H10O -NH3 [7|1][1-7]	C47H58N6O13
916.4121	916.4127	-0.0007	-0.7	53.27	z6 -CH3OH -H2O [6|7][2-7]	C51H57N5O11
932.4364	932.4440	-0.0076	-8.1	61.58	a6 -H2O -NH3 [1|2][1-6]	C52H61N5O11
932.4364	932.4400	-0.0036	-3.8	61.58	M -C9H10O [7|1][1-7]	C47H61N7O13
933.4439	933.4393	0.0046	4.9	64.59	b6 -CH3OH -H2O [7|1][1-6]	C51H60N6O11
1017.4548	1017.4604	-0.0056	-5.5	8.22	M -CH3OH -NH3 [7|1][1-7]	C55H64N6O13
1034.4855	1034.4870	-0.0015	-1.5	16.44	M -CH3OH [7|1][1-7]	C55H67N7O13

NMR

See Christiansen et al., J. Nat. Prod. 2008, 1881-1886

[D-Asp^{3},(*E*)-Dhb^{7}]MC-HtyW (20)

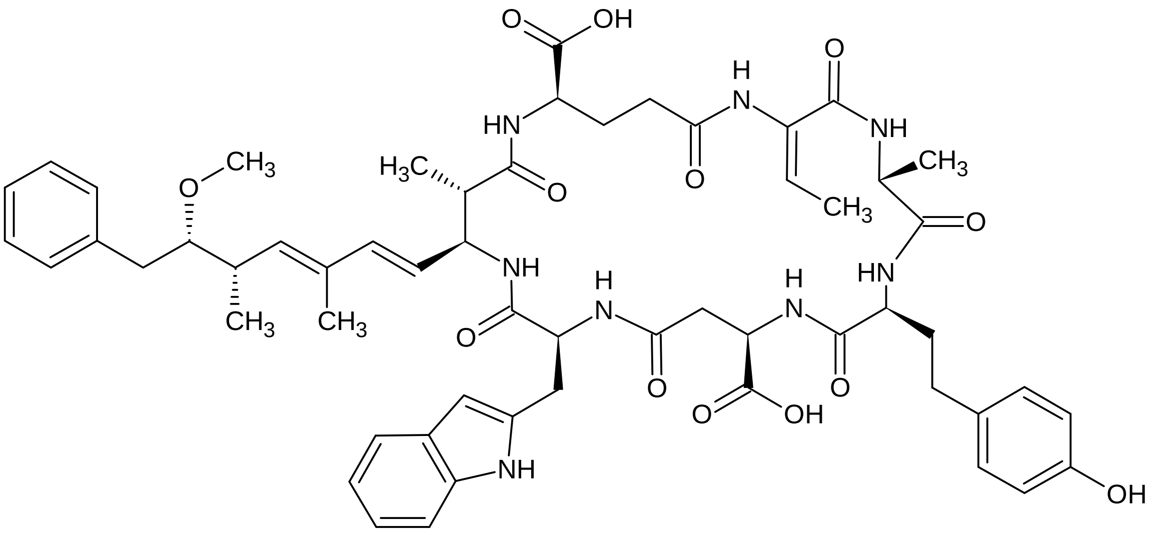

D-Ala | Hty | D-Asp | Trp | Adda | D-Glu | Dhb

Formula (M)	Ion	Meas. m/z	Pred. m/z	Diff (mDa)	Diff (ppm)	Iso Score
C57 H70 N8 O13	[M+H]+	1075.5133	1075.5135 -	0.2 -	0.19	69.04

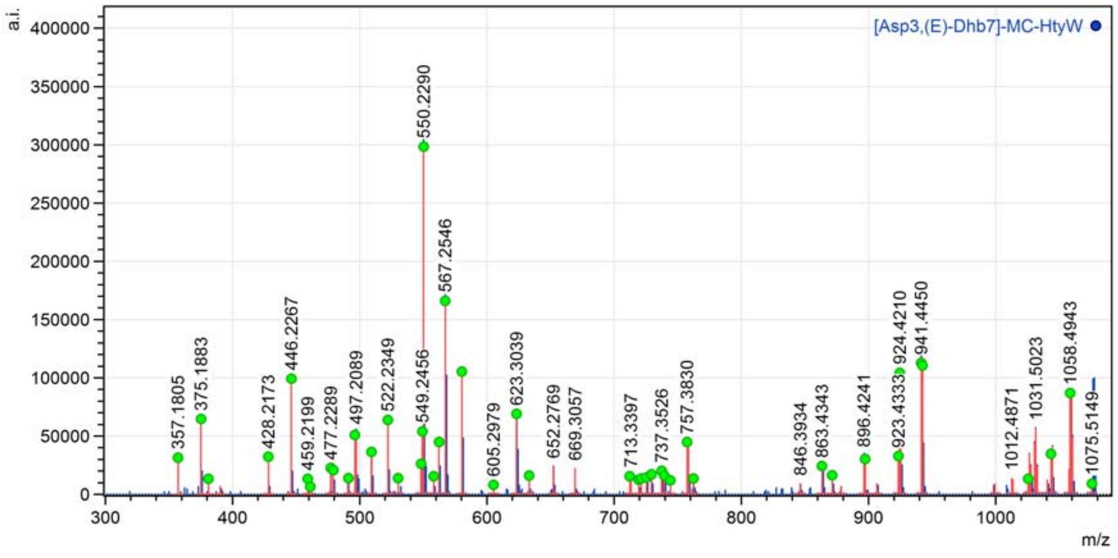

Length	Mo. Mass	Av. Mass	Coverage	Matched Int.
7 (Cyclic)	1074.5062	1075.2140	100.0 %	77.3 %

Meas. m/z	Calc. m/z	δ (Da)	δ (ppm)	Rel. Int. (%)	Annotation	Formula
357.1805	357.1809	-0.0004	-1.1	10.56	z3 -C9H10O -H2O [7|1][5-7]	C20H24N2O4
375.1883	375.1914	-0.0031	-8.4	21.68	z3 -C9H10O [7|1][5-7]	C20H26N2O5
381.1675	381.1769	-0.0094	-24.6	4.41	c3 [7|1][1-3]	C17H24N4O6
428.2173	428.2180	-0.0007	-1.7	10.70	z4 -C9H10O -H2O [1|2][4-7]	C23H29N3O5
446.2267	446.2286	-0.0019	-4.2	33.20	z4 -C9H10O [1|2][4-7]	C23H31N3O6
459.2199	459.2278	-0.0080	-17.3	4.29	z3 -CH3OH -H2O [7|1][5-7]	C28H30N2O4
459.2199	459.2153	0.0046	10.1	4.29	z3 -C9H11O -H2O [6|7][5-7]	C27H28N3O4
461.1987	461.2031	-0.0043	-9.4	1.98	b4 [5|6][1-4]	C22H28N4O7
477.2289	477.2384	-0.0095	-19.8	7.36	b3 -CH3OH -NH3 [4|5][1-3]	C28H32N2O5
477.2289	477.2258	0.0031	6.5	7.36	z3 -C9H11O [6|7][5-7]	C27H30N3O5
479.1925	479.1925	0.0000	0.1	6.73	b3 [1|2][1-3]	C25H26N4O6
491.2515	491.2540	-0.0025	-5.1	4.35	b3 -H2O -NH3 [4|5][1-3]	C29H34N2O5
496.2198	496.2191	0.0007	1.4	16.87	c3 [1|2][1-3]	C25H29N5O6
509.2641	509.2646	-0.0005	-1.0	11.92	b3 -NH3 [4|5][1-3]	C29H36N2O6
522.2349	522.2347	0.0002	0.3	21.13	a4 [7|1][1-4]	C27H31N5O6
530.2561	530.2649	-0.0089	-16.8	4.36	z4 -CH3OH -H2O [1|2][4-7]	C31H35N3O5
548.2772	548.2755	0.0017	3.1	8.45	b4 -CH3OH -NH3 [4|5][1-4]	C31H37N3O6
549.2456	549.2384	0.0072	13.1	17.85	z3 -CH3OH -NH3 [5|6][5-7]	C34H32N2O5
550.2290	550.2296	-0.0007	-1.2	100.00	b4 [7|1][1-4]	C28H31N5O7
558.2212	558.2195	0.0017	3.1	4.88	b5 -H2O [5|6][1-5]	C26H31N5O9
562.2896	562.2912	-0.0016	-2.8	14.80	b4 -H2O -NH3 [4|5][1-4]	C32H39N3O6
567.2546	567.2562	-0.0016	-2.8	55.54	c4 [7|1][1-4]	C28H34N6O7
580.2989	580.3017	-0.0029	-4.9	35.08	b4 -NH3 [4|5][1-4]	C32H41N3O7
605.2979	605.2970	0.0009	1.6	2.46	z5 -C9H10O -H2O [2|3][3-7]	C33H40N4O7
623.3039	623.3075	-0.0036	-5.8	22.97	z5 -C9H10O [2|3][3-7]	C33H42N4O8
633.2742	633.2667	0.0074	11.8	5.12	b5 [6|7][1-5]	C32H36N6O8
712.3425	712.3341	0.0085	11.9	4.93	b4 -CH3OH [2|3][1-4]	C39H45N5O8
712.3425	712.3341	0.0085	11.9	4.93	z5 -C9H10O [5|6][3-7]	C39H45N5O8
719.3325	719.3399	-0.0074	-10.3	3.93	a6 -C9H10O -NH3 [2|3][1-6]	C37H46N6O9
721.3600	721.3596	0.0005	0.7	4.31	z5 -H2O -H2O [2|3][3-7]	C42H48N4O7
725.3582	725.3545	0.0037	5.2	4.60	z5 -CH3OH [2|3][3-7]	C41H48N4O8
729.3588	729.3647	-0.0058	-8.0	5.50	a4 -H2O -NH3 [1|2][1-4]	C44H48N4O6
729.3588	729.3606	-0.0018	-2.5	5.50	b5 -C9H10O [7|1][1-5]	C39H48N6O8
737.3526	737.3505	0.0021	2.9	6.56	b6 -C9H10O -H2O [4|5][1-6]	C37H48N6O10
739.3658	739.3701	-0.0044	-5.9	5.19	b5 -H2O -NH3 [4|5][1-5]	C42H50N4O8
744.3032	744.2988	0.0044	5.9	3.79	b6 -H2O [5|6][1-6]	C37H41N7O10
757.3830	757.3807	0.0023	3.1	14.84	b5 -NH3 [4|5][1-5]	C42H52N4O9
762.3153	762.3093	0.0059	7.8	4.32	b6 [5|6][1-6]	C37H43N7O11
863.4343	863.4338	0.0005	0.6	7.89	b5 [7|1][1-5]	C48H58N6O9
871.4274	871.4236	0.0038	4.4	5.26	b6 -H2O [4|5][1-6]	C46H58N6O11

897.4226	897.4182	0.0045	5.0	9.88	b6 -CH3OH -NH3 [6|7][1-6]	C51H56N6O9
923.4333	923.4298	0.0035	3.8	10.67	M -C9H10O -H2O [7|1][1-7]	C48H58N8O11
924.4210	924.4138	0.0073	7.8	34.76	M -C9H10O -NH3 [7|1][1-7]	C48H57N7O12
925.4215	925.4131	0.0085	9.1	31.95	z6 -CH3OH -H2O [6|7][2-7]	C52H56N6O10
941.4450	941.4403	0.0046	4.9	37.54	M -C9H10O [7|1][1-7]	C48H60N8O12
942.4466	942.4396	0.0069	7.4	36.78	b6 +CO -CH3OH [6|7][1-6]	C52H59N7O10
942.4466	942.4396	0.0069	7.4	36.78	b6 -CH3OH -H2O [7|1][1-6]	C52H59N7O10
1025.4828	1025.4767	0.0061	5.9	4.03	M -CH3OH -H2O [7|1][1-7]	C56H64N8O11
1043.4912	1043.4873	0.0039	3.7	11.25	M -CH3OH [7|1][1-7]	C56H66N8O12
1058.4943	1058.4870	0.0074	7.0	28.80	M -NH3 [7|1][1-7]	C57H67N7O13

NMR

See Niedermeyer et al., Nat. Prod. Bioprospect., 2014, accepted

[D-Asp^3,MSer^7]MC-YHar (21)

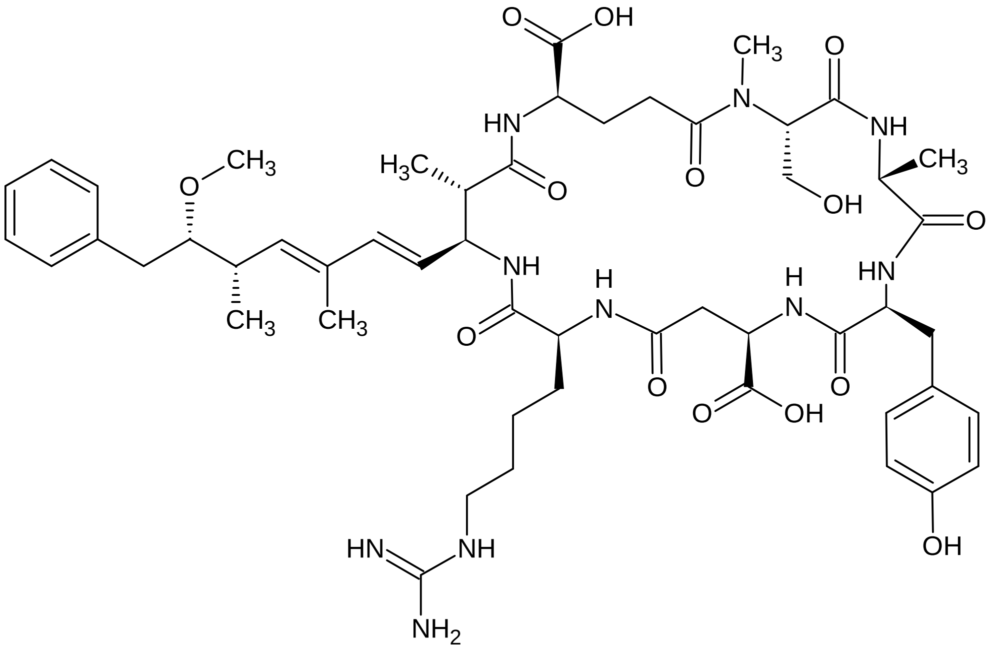

D-Ala | Tyr | D-Asp | Har | Adda | D-Glu | Mser

Formula (M)	Ion	Meas. m/z	Pred. m/z	Diff (mDa)	Diff (ppm)	Iso Score
C52 H74 N10 O14	[M+H]+	1063.5482	1063.5459	2.3	2.16	91.89

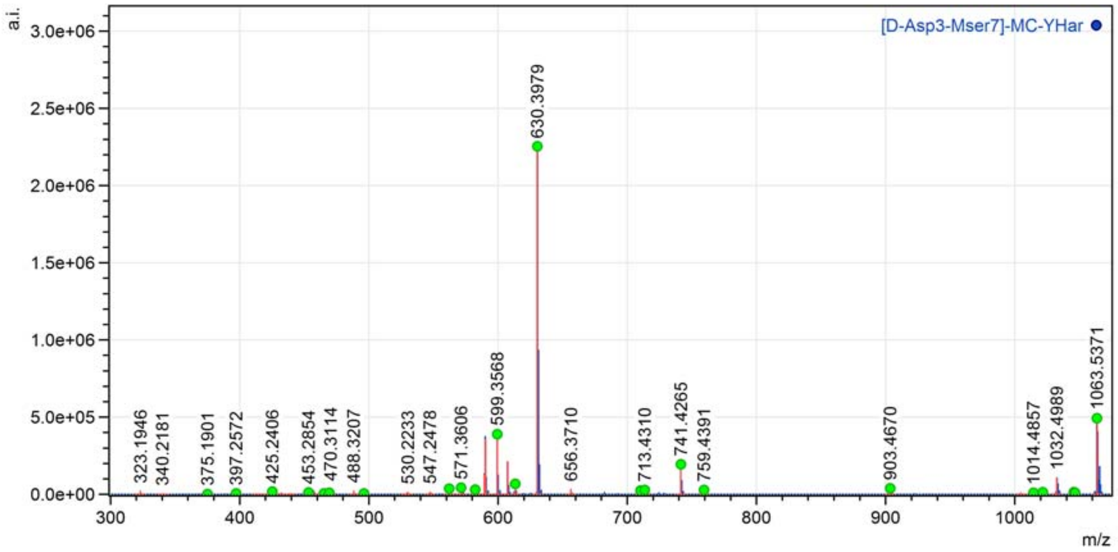

Length	Mo. Mass	Av. Mass	Coverage	Matched Int.
7 (Cyclic)	1062.5386	1063.2050	100.0 %	79.3 %

Meas. m/z	Calc. m/z	δ (Da)	δ (ppm)	Rel. Int. (%)	Annotation	Formula
375.1901	375.1914	-0.0014	-3.7	0.07	z3 -C9H10O -H2O [7|1][5-7]	C20H26N2O5
397.2572	397.2486	0.0086	21.7	0.24	a2 -H2O [4|5][1-2]	C24H32N2O3

425.2406	425.2435	-0.0029	-6.8	0.79	b2 -H2O [4|5][1-2]	C25H32N2O4
453.2854	453.2946	-0.0092	-20.3	0.55	c3 -C9H11O -CN2H2 [3|4][1-3]	C22H38N5O5
465.2769	465.2820	-0.0051	-11.0	0.27	b3 -C9H10O [2|3][1-3]	C22H36N6O5
469.3284	469.3286	-0.0001	-0.3	0.45	c2 -CH3OH [3|4][1-2]	C26H40N6O2
496.3198	496.3242	-0.0044	-8.9	0.23	c3 -C9H10O [3|4][1-3]	C23H41N7O5
562.2628	562.2633	-0.0005	-0.9	1.62	b4 +CO -C9H11O -CH5N3 [2|3][1-4]	C27H37N4O9
571.3606	571.3602	0.0003	0.6	1.85	a3 [2|3][1-3]	C30H46N6O5
582.3321	582.3286	0.0035	6.0	1.30	z3 [5|6][5-7]	C31H43N5O6
582.3321	582.3286	0.0035	6.0	1.30	b3 -NH3 [2|3][1-3]	C31H43N5O6
599.3568	599.3552	0.0017	2.8	17.31	b3 [2|3][1-3]	C31H46N6O6
613.3713	613.3708	0.0005	0.7	3.00	b3 [3|4][1-3]	C32H48N6O6
630.3979	630.3974	0.0006	0.9	100.00	c3 [3|4][1-3]	C32H51N7O6
710.3849	710.3872	-0.0023	-3.2	1.06	b4 -H2O [2|3][1-4]	C36H51N7O8
713.4310	713.4345	-0.0035	-4.9	1.28	c4 -H2O [3|4][1-4]	C36H56N8O7
741.4265	741.4182	0.0083	11.2	8.64	a5 -CN2H2 -H2O [2|3][1-5]	C38H56N6O9
759.4391	759.4400	-0.0009	-1.2	1.27	c4 +CO [3|4][1-4]	C37H58N8O9
903.4670	903.4571	0.0100	11.0	1.67	c6 +CO -C9H10O [1|2][1-6]	C41H62N10O13
1014.4857	1014.4931	-0.0074	-7.3	0.36	M -CH3OH -NH3 [7|1][1-7]	C51H67N9O13
1021.5280	1021.5241	0.0040	3.9	0.60	M -CN2H2 [7|1][1-7]	C51H72N8O14
1045.5299	1045.5353	-0.0054	-5.2	0.58	M -H2O [7|1][1-7]	C52H72N10O13
1046.5286	1046.5193	0.0093	8.9	0.42	M -NH3 [7|1][1-7]	C52H71N9O14
1063.5371	1063.5459	-0.0088	-8.2	21.88	M [7|1][1-7]	C52H74N10O14

NMR

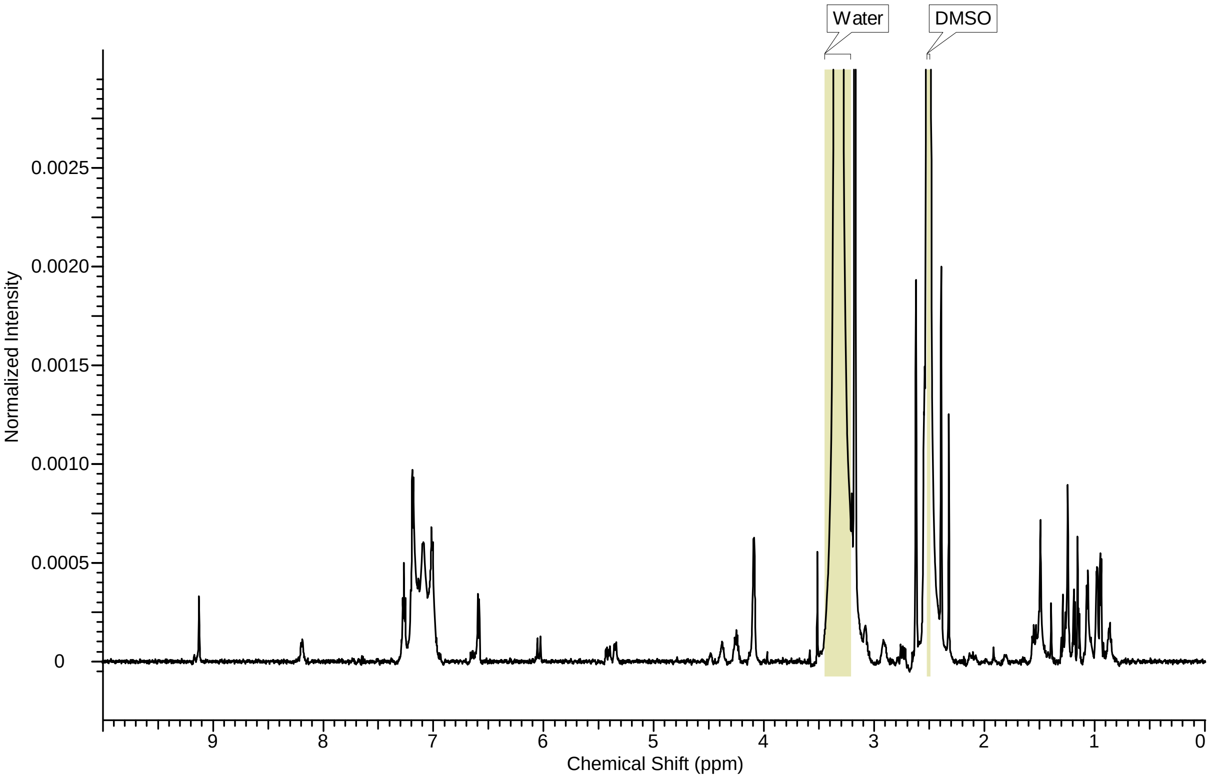

1H-NMR spectrum agrees with the postulated structure. Ser is the biogenetic precursor of dehydroalanine, which is more widely found in *Microcystis* microcystins.

[D-Glu(OMe)[6]]MC-YR (22)

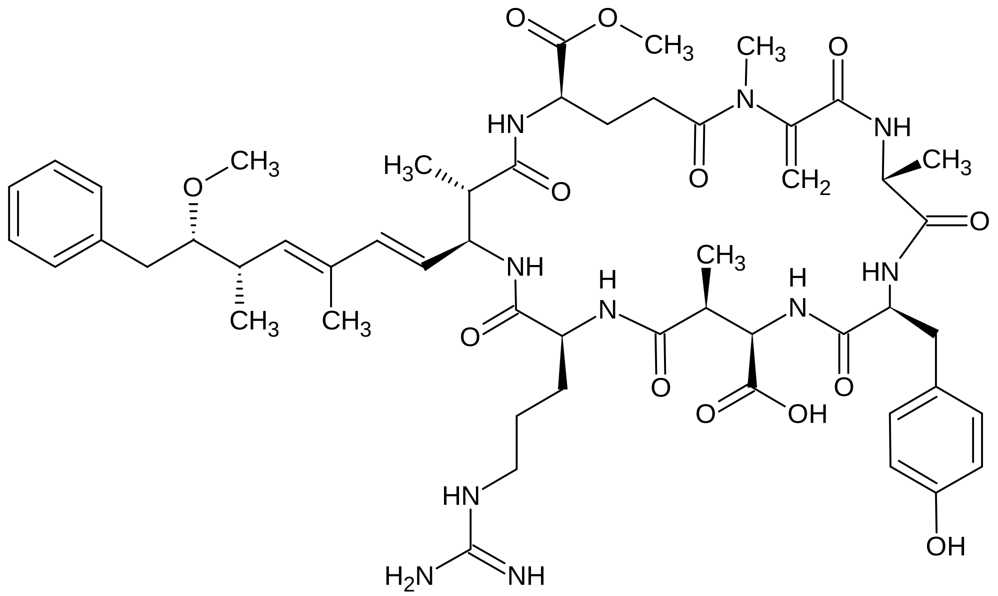

D-Ala | Tyr | D-MeAsp | Arg | Adda | D-Glu(OMe) | Mdha

Formula (M)	Ion	Meas. m/z	Pred. m/z	Diff (mDa)	Diff (ppm)	Iso Score
C53 H74 N10 O13	[M+H]+	1059.5526	1059.5510	1.6	1.51	100.00

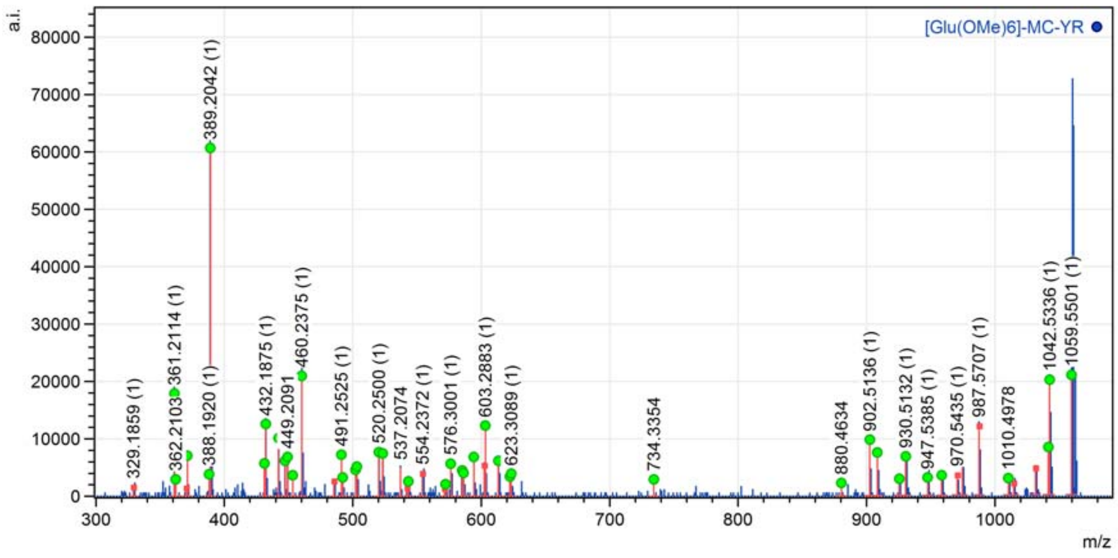

Length	Mo. Mass	Av. Mass	Coverage	Matched Int.
7 (Cyclic)	1058.5437	1059.2163	100.0 %	85.4 %

Meas. m/z	Calc. m/z	δ (Da)	δ (ppm)	Rel. Int. (%)	Annotation	Formula
361.2114	361.2122	-0.0007	-2.1	29.48	a3 -C9H10O -NH3 [4|5][1-3]	C20H28N2O4
362.2103	362.2115	-0.0012	-3.3	4.88	z2 -CH3OH -CH5N3 [5|6][6-7]	C24H27NO2
371.1696	371.1727	-0.0031	-8.3	11.66	z3 -C9H11O -NH3 [7|1][5-7]	C21H24NO5
388.1920	388.1993	-0.0072	-18.6	6.36	z3 -C9H11O [7|1][5-7]	C21H27N2O5

388.1920	388.1993	-0.0072	-18.6	6.36	b3 -C9H11O -NH3 [4|5][1-3]	C21H27N2O5
389.2042	389.2071	-0.0029	-7.6	100.00	z3 -C9H10O [7|1][5-7]	C21H28N2O5
431.2065	431.2037	0.0027	6.4	9.47	b3 -H2O [1|2][1-3]	C20H26N6O5
432.1875	432.1878	-0.0002	-0.5	20.76	b3 -NH3 [1|2][1-3]	C20H25N5O6
432.1875	432.1878	-0.0002	-0.5	20.76	z3 [4|5][5-7]	C20H25N5O6
442.2310	442.2336	-0.0026	-5.9	16.77	z4 -C9H10O -H2O [1|2][4-7]	C24H31N3O5
447.1871	447.1874	-0.0004	-0.8	10.16	b4 [6|7][1-4]	C21H26N4O7
449.2091	449.2143	-0.0052	-11.6	11.32	b3 [1|2][1-3]	C20H28N6O6
453.2794	453.2860	-0.0066	-14.6	6.05	b2 -NH3 [3|4][1-2]	C26H36N4O3
453.2794	453.2860	-0.0066	-14.6	6.05	z2 [5|6][6-7]	C26H36N4O3
460.2375	460.2442	-0.0067	-14.5	34.63	z4 -C9H10O [1|2][4-7]	C24H33N3O6
460.2375	460.2442	-0.0067	-14.5	34.63	b4 -C9H10O -NH3 [4|5][1-4]	C24H33N3O6
491.2525	491.2540	-0.0016	-3.2	11.95	z3 -CH3OH [7|1][5-7]	C29H34N2O5
491.2525	491.2540	-0.0016	-3.2	11.95	b3 -CH3OH -NH3 [4|5][1-3]	C29H34N2O5
492.2548	492.2565	-0.0017	-3.5	5.44	a4 [7|1][1-4]	C22H33N7O6
502.2389	502.2409	-0.0020	-3.9	7.62	b4 -H2O [7|1][1-4]	C23H31N7O6
503.2279	503.2249	0.0030	5.9	8.52	b4 -NH3 [7|1][1-4]	C23H30N6O7
503.2279	503.2249	0.0030	5.9	8.52	z4 [4|5][4-7]	C23H30N6O7
520.2500	520.2514	-0.0014	-2.7	12.68	b4 [7|1][1-4]	C23H33N7O7
523.2777	523.2803	-0.0026	-4.9	12.28	z3 [7|1][5-7]	C30H38N2O6
523.2777	523.2803	-0.0026	-4.9	12.28	b3 -NH3 [4|5][1-3]	C30H38N2O6
543.2551	543.2562	-0.0011	-2.0	4.28	b5 -CN2H2 -H2O [6|7][1-5]	C26H34N6O7
572.2371	572.2351	0.0020	3.4	3.44	b5 -H2O [5|6][1-5]	C27H33N5O9
576.3001	576.3068	-0.0067	-11.7	9.40	z4 -H2O [1|2][4-7]	C33H41N3O6
576.3001	576.3068	-0.0067	-11.7	9.40	b4 -H2O -NH3 [4|5][1-4]	C33H41N3O6
585.2837	585.2780	0.0057	9.7	7.42	b5 -H2O [6|7][1-5]	C27H36N8O7
586.2661	586.2620	0.0041	7.0	6.67	z5 [4|5][3-7]	C27H35N7O8
586.2661	586.2620	0.0041	7.0	6.67	b5 -NH3 [6|7][1-5]	C27H35N7O8
594.3128	594.3174	-0.0046	-7.7	11.29	b4 -NH3 [4|5][1-4]	C33H43N3O7
594.3128	594.3174	-0.0046	-7.7	11.29	z4 [1|2][4-7]	C33H43N3O7
594.3128	594.3048	0.0080	13.4	11.29	a5 -C9H11O -NH3 [4|5][1-5]	C32H41N4O7
603.2883	603.2885	-0.0003	-0.5	20.35	b5 [6|7][1-5]	C27H38N8O8
613.3634	613.3708	-0.0074	-12.0	10.17	b3 [3|4][1-3]	C32H48N6O6
622.2960	622.2997	-0.0037	-5.9	5.67	z5 -C9H11O [2|3][3-7]	C33H41N4O8
622.2960	622.2997	-0.0037	-5.9	5.67	b5 -C9H11O -NH3 [4|5][1-5]	C33H41N4O8
622.2960	622.2997	-0.0037	-5.9	5.67	z5 -C9H11O -CH5N3 [5|6][3-7]	C33H41N4O8
623.3089	623.3075	0.0014	2.2	6.47	z5 -C9H10O [2|3][3-7]	C33H42N4O8
623.3089	623.3075	0.0014	2.2	6.47	b5 -C9H10O -NH3 [4|5][1-5]	C33H42N4O8
734.3354	734.3396	-0.0041	-5.6	4.88	z6 -C9H10O -H2O [3|4][2-7]	C38H47N5O10
880.4634	880.4716	-0.0082	-9.3	3.65	b6 -CH3OH -H2O [3|4][1-6]	C47H61N9O8
902.5136	902.5135	0.0001	0.2	16.05	a6 [3|4][1-6]	C47H67N9O9
908.4530	908.4512	0.0017	1.9	12.34	M -C9H10O -NH3 [7|1][1-7]	C44H61N9O12
925.4764	925.4818	-0.0054	-5.8	4.81	a6 -H2O -NH3 [1|2][1-6]	C49H64N8O10
925.4764	925.4778	-0.0014	-1.5	4.81	M -C9H10O [7|1][1-7]	C44H64N10O12
930.5132	930.5084	0.0048	5.2	11.20	b6 [3|4][1-6]	C48H67N9O10
930.5132	930.5084	0.0048	5.2	11.20	a6 -H2O [7|1][1-6]	C48H67N9O10
947.5385	947.5349	0.0036	3.8	5.07	c6 [3|4][1-6]	C48H70N10O10

958.5024	958.5033	-0.0009	-0.9	5.71	b6 -H2O [7|1][1-6]	C49H67N9O11
1010.4978	1010.4982	-0.0004	-0.4	4.80	M -CH3OH -NH3 [7|1][1-7]	C52H67N9O12
1041.5312	1041.5404	-0.0091	-8.8	13.73	M -H2O [7|1][1-7]	C53H72N10O12
1042.5336	1042.5244	0.0092	8.8	33.07	M -NH3 [7|1][1-7]	C53H71N9O13
1059.5501	1059.5510	-0.0008	-0.8	34.46	M [7|1][1-7]	C53H74N10O13

NMR

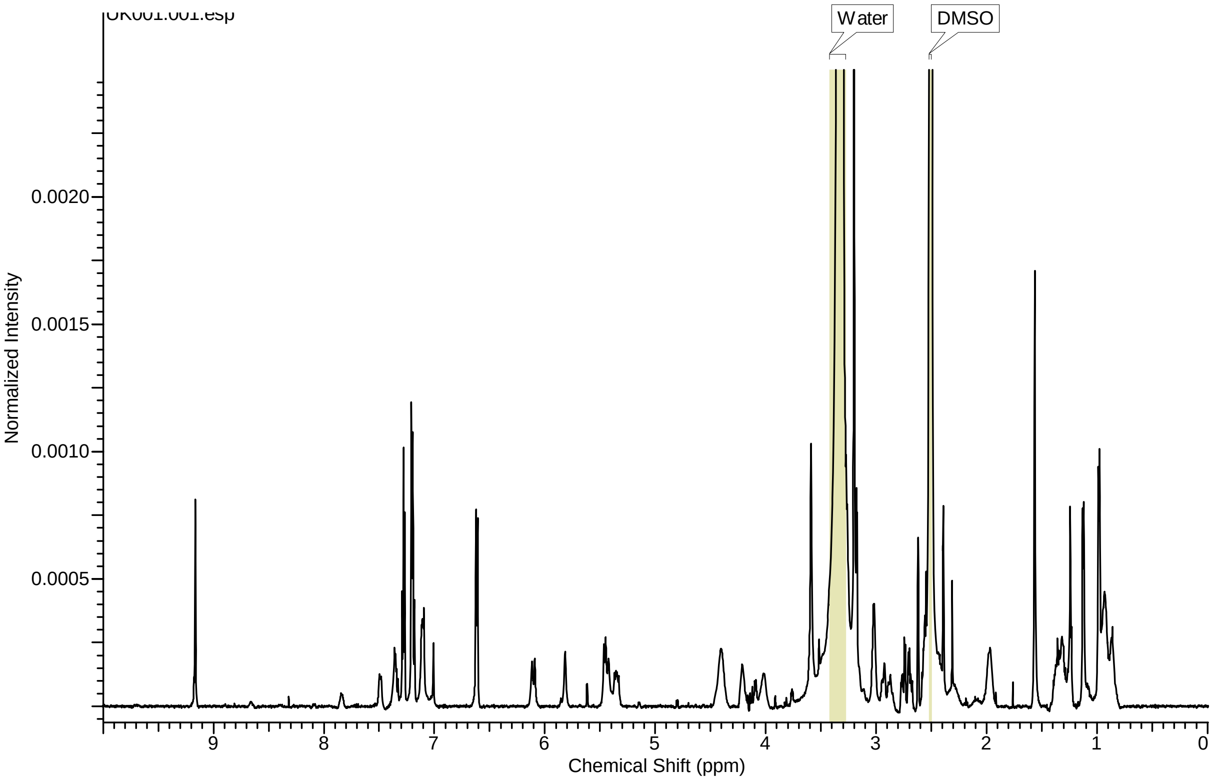

1H-NMR spectrum agrees with the postulated structure. This compound has not been described in the literature before.

Nodularin (23)

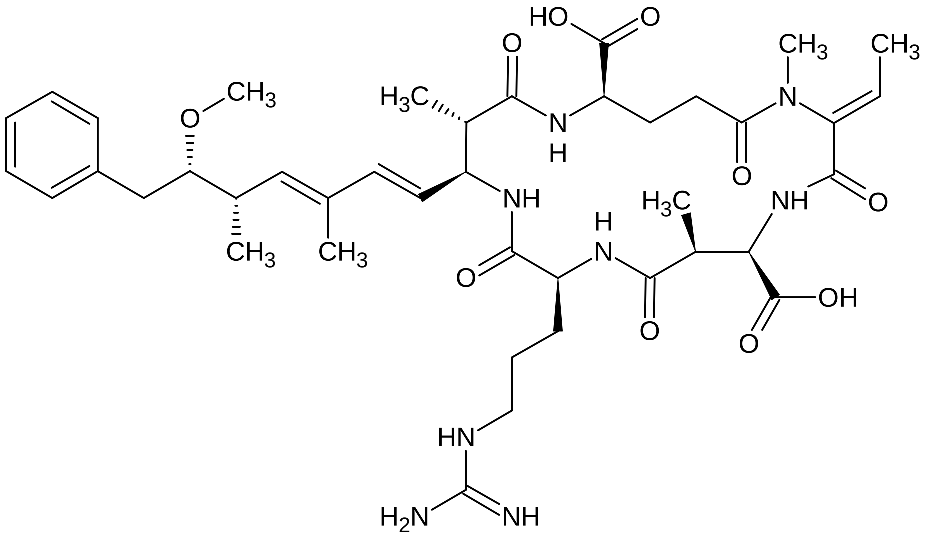

D-MeAsp | Arg | Adda | D-Glu | Mdhb

Formula (M)	Ion	Meas. m/z	Pred. m/z	Diff (mDa)	Diff (ppm)	Iso Score
C41 H60 N8 O10	[M+H]+	825.4541	825.4505	3.6	4.36	89.42

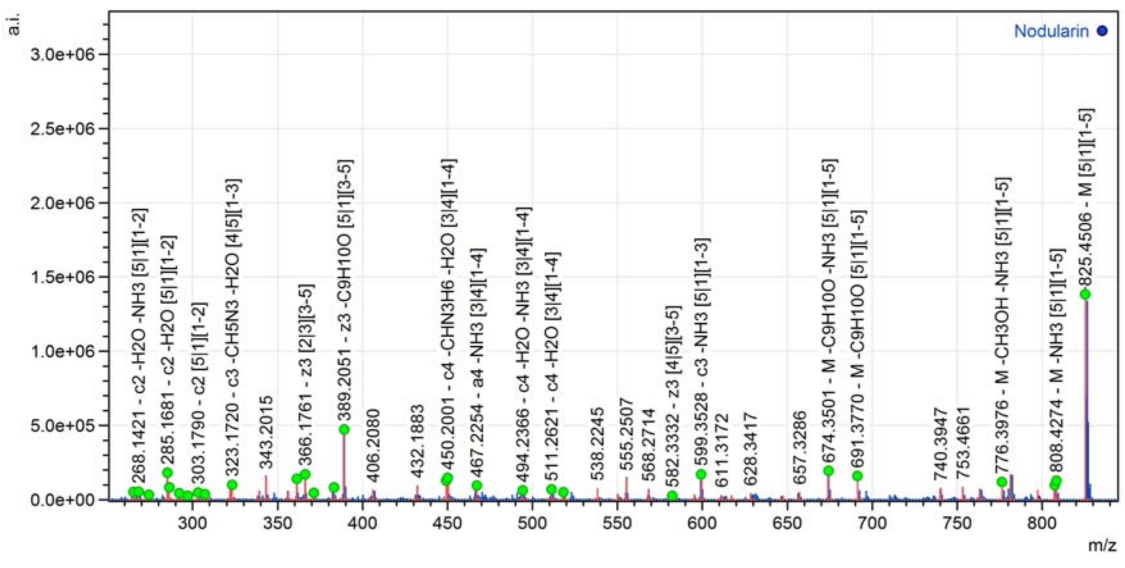

Length	Mo. Mass	Av. Mass	Coverage	Matched Int.
5 (Cyclic)	824.4432	824.9646	100.0 %	72.8 %

Meas. m/z	Calc. m/z	δ (Da)	δ (ppm)	Rel. Int. (%)	Annotation	Formula
265.1598	265.1587	0.0011	4.3	3.91	z1 -CH3OH [3|4][5-5]	C19H20O
268.1421	268.1404	0.0017	6.2	3.91	b2 -H2O [5|1][1-2]	C11H17N5O3
274.1440	274.1438	0.0002	0.7	2.38	z2 -C9H10O -H2O [4|5][4-5]	C16H19NO3
285.1681	285.1670	0.0011	3.9	13.16	c2 -H2O [5|1][1-2]	C11H20N6O3
286.1529	286.1510	0.0019	6.7	5.95	b2 [5|1][1-2]	C11H19N5O4
292.1500	292.1543	-0.0043	-14.7	3.25	z2 -C9H10O [4|5][4-5]	C16H21NO4

292.1500	292.1543	-0.0043	-14.7	3.25	b2 -C9H10O -NH3 [2|3][1-2]	C16H21NO4
297.1912	297.1849	0.0063	21.1	1.91	z1 [3|4][5-5]	C20H24O2
303.1790	303.1775	0.0015	4.9	3.53	c2 [5|1][1-2]	C11H22N6O4
307.1277	307.1288	-0.0012	-3.9	2.80	b3 -CH5N3 -NH3 [4|5][1-3]	C15H18N2O5
307.1277	307.1288	-0.0012	-3.9	2.80	z3 -CH5N3 [2|3][3-5]	C15H18N2O5
323.1720	323.1714	0.0006	1.9	7.21	b3 -CN2H2 -H2O [4|5][1-3]	C15H22N4O4
361.2099	361.2122	-0.0023	-6.3	10.18	a3 -C9H10O -NH3 [2|3][1-3]	C20H28N2O4
366.1761	366.1772	-0.0011	-3.0	12.44	z3 [2|3][3-5]	C16H23N5O5
366.1761	366.1772	-0.0011	-3.0	12.44	b3 -NH3 [4|5][1-3]	C16H23N5O5
371.1945	371.1965	-0.0020	-5.5	3.37	z3 -C9H10O -H2O [5|1][3-5]	C21H26N2O4
383.2016	383.2037	-0.0022	-5.6	5.97	b3 [4|5][1-3]	C16H26N6O5
389.2051	389.2071	-0.0020	-5.1	34.19	z3 -C9H10O -CH5N3 [4|5][3-5]	C21H28N2O5
389.2051	389.2071	-0.0020	-5.1	34.19	b3 -C9H10O -NH3 [2|3][1-3]	C21H28N2O5
389.2051	389.2071	-0.0020	-5.1	34.19	z3 -C9H10O [5|1][3-5]	C21H28N2O5
389.2051	389.2071	-0.0020	-5.1	34.19	z3 -C9H10O -CH5N3 [3|4][3-5]	C21H28N2O5
449.2136	449.2143	-0.0008	-1.7	9.21	a4 -H2O -NH3 [3|4][1-4]	C20H28N6O6
450.2001	450.1983	0.0018	3.9	10.69	a4 -NH3 -NH3 [3|4][1-4]	C20H27N5O7
467.2254	467.2249	0.0005	1.2	7.01	a4 -NH3 [3|4][1-4]	C20H30N6O7
494.2366	494.2358	0.0008	1.6	4.55	b4 -H2O [3|4][1-4]	C21H31N7O7
511.2621	511.2623	-0.0002	-0.4	5.04	c4 -H2O [3|4][1-4]	C21H34N8O7
518.2465	518.2497	-0.0032	-6.2	3.75	z4 -C9H10O -CH5N3 [4|5][2-5]	C26H35N3O8
518.2465	518.2497	-0.0032	-6.2	3.75	b4 -C9H10O -NH3 [2|3][1-4]	C26H35N3O8
518.2465	518.2497	-0.0032	-6.2	3.75	z4 -C9H10O [1|2][2-5]	C26H35N3O8
582.3332	582.3286	0.0046	7.9	1.92	z3 [4|5][3-5]	C31H43N5O6
582.3332	582.3286	0.0046	7.9	1.92	z3 [3|4][3-5]	C31H43N5O6
582.3332	582.3286	0.0046	7.9	1.92	b3 -NH3 [1|2][1-3]	C31H43N5O6
582.3332	582.3286	0.0046	7.9	1.92	b3 -NH3 [5|1][1-3]	C31H43N5O6
599.3528	599.3552	-0.0023	-3.9	12.45	b3 [5|1][1-3]	C31H46N6O6
599.3528	599.3552	-0.0023	-3.9	12.45	b3 [1|2][1-3]	C31H46N6O6
674.3501	674.3508	-0.0007	-1.1	14.06	M -C9H10O -NH3 [5|1][1-5]	C32H47N7O9
691.3770	691.3774	-0.0003	-0.5	11.66	M -C9H10O [5|1][1-5]	C32H50N8O9
776.3976	776.3978	-0.0002	-0.2	8.71	M -CH3OH -NH3 [5|1][1-5]	C40H53N7O9
807.4413	807.4400	0.0014	1.7	7.07	M -H2O [5|1][1-5]	C41H58N8O9
808.4274	808.4240	0.0034	4.2	9.25	M -NH3 [5|1][1-5]	C41H57N7O10
825.4506	825.4505	0.0001	0.1	100.00	M [5|1][1-5]	C41H60N8O10

NMR

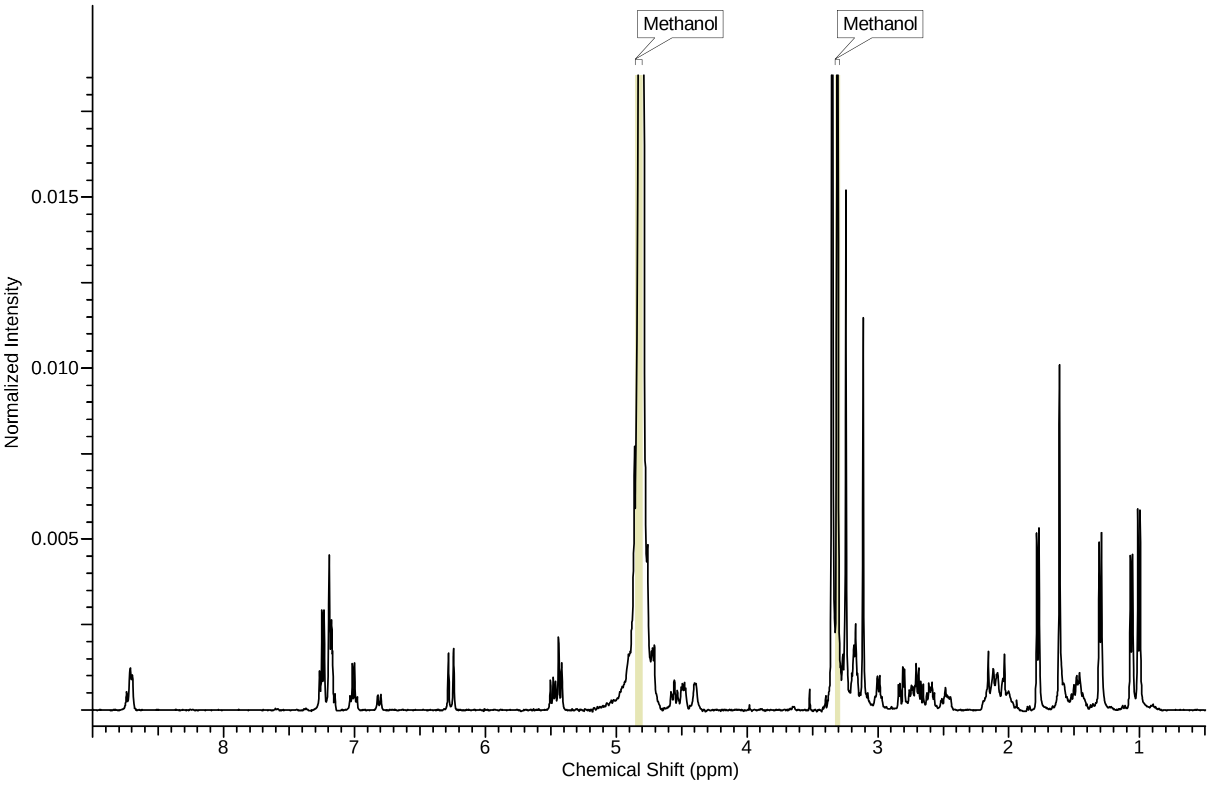

^{1}H-NMR spectrum agrees with the postulated structure and is comparable to the spectrum described for Nodularin by Namikoshi et al., J. Org. Chem. 1994, 2349-2357
